# Zinc-Catalyzed Cyclization of Alkynyl Derivatives: Substrate Scope and Mechanistic Insights

**DOI:** 10.1021/acs.inorgchem.4c00832

**Published:** 2024-07-16

**Authors:** Marc Martínez de Sarasa Buchaca, Miguel A. Gaona, Luis F. Sánchez-Barba, Andrés Garcés, Ana M. Rodríguez, Antonio Rodríguez-Diéguez, Felipe de la Cruz-Martínez, José A. Castro-Osma, Agustín Lara-Sánchez

**Affiliations:** † Universidad de Castilla-La Mancha, Departamento de Química Inorgánica, Orgánica y Bioquímica-Centro de Innovación en Química Avanzada (ORFEO−CINQA), Facultad de Ciencias y Tecnologías Químicas, Instituto Regional de Investigación Científica Aplicada-IRICA, Ciudad Real 13071, Spain; ‡ Departamento de Biología y Geología, Física y Química Inorgánica, Universidad Rey Juan Carlos, Móstoles 28933, Spain; § Departamento de Química Inorgánica, Facultad de Ciencias, Universidad de Granada, Granada 18071, Spain; ∥ Departamento de Química Inorgánica, Orgánica y Bioquímica-Centro de Innovación en Química Avanzada (ORFEO−CINQA), Facultad de Farmacia, Universidad de Castilla-La Mancha, Albacete 02071, Spain

## Abstract

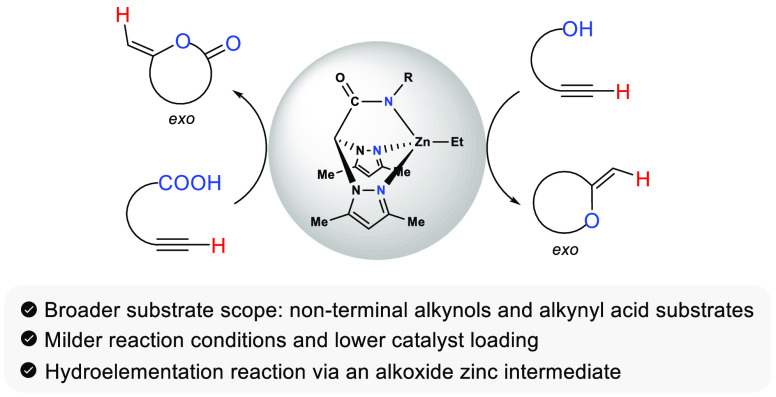

Novel alkyl zinc complexes supported by acetamidate/thioacetamidate heteroscorpionate ligands have been successfully synthesized and characterized. These complexes exhibited different coordination modes depending on the electronic and steric effects of the acetamidate/thioacetamidate moiety. Their catalytic activity has been tested toward the hydroelementation reactions of alkynyl alcohol/acid substrates, affording the corresponding enol ether/unsaturated lactone products under mild reaction conditions. Kinetic studies have been performed and confirmed that reactions are first-order in [catalyst] and zero-order in [alkynyl substrate]. DFT calculations supported a reaction mechanism through the formation of the catalytically active species, an alkoxide-zinc intermediate, by a protonolysis reaction of the Zn–alkyl bond with the alcohol group of the substrate. Based on the experimental and theoretical results, a catalytic cycle has been proposed.

## Abstract 2

Zinc-catalyzed hydroelementation of alkynyl alcohols and alkynyl acids.

## Introduction

Oxygen-containing heterocycles have garnered considerable interest from both the scientific and synthetic communities due to their wide presence in natural products and bioactive scaffolds. Because of this, diverse strategies have been developed for their synthesis such as reductive etherification,^1^ Oxa-Michael addition,^2^ transition metal-catalyzed π-allyl cation-based transformation,^3^ Ag-catalyzed nucleophilic substitution,^4^ hetero Diels–Alder, and hydroalkoxylation/carboxylation of alkene derivatives.^5^

In recent years, the hydrofunctionalization of alkynes has been a very interesting and effective route for the design and synthesis of functionalized heterocycles with a complete atom-economical and step-economical strategy under mild reaction conditions.^6^ In this regard, hydroalkoxylation of alkynes has provided reliable routes to produce oxygen-bearing heterocycles.^7^ Initially, hydroalkoxylation of alkenes was widely explored for the synthesis of saturated heterocycles.^8^ However, using alkyne substrates instead of alkenes offers wider synthetic possibilities due to the possibility of accessing and isolating the reactive “enol ether” intermediates, which have been reported to participate in different cascade processes.^9^ According to the Baldwin’s rules, the hydroalkoxylation reaction can potentially lead to the formation of the *exo*- and/or *endo*-heterocycles.^10^ The regioselectivity of the process has been found to be dependent on the metal used, the length of the hydrocarbon chain linking the alcohol and alkyne moieties, and whether the alkyne functionality is terminal or internal.^8b,11^

In recent decades, an extensive library of catalysts based on metal complexes has been published for this transformation. Transition metal compounds comprising iridium,^12^ rhodium,^13^ gold,^14^ palladium,^15^ ruthenium,^16^ silver,^17^ platinum,^5a,18^ iron,^19^ and copper^20^ have shown to catalyze this reaction efficiently via a metal–alkynyl species by selectively activating the alkyne moiety of the substrate. In addition, *s*-block and rare-earth complexes have also proven to be highly active for this transformation.^21,22^ However, in this case, the reaction proceeds via the formation of a metal–alkoxide intermediate, which is generated by activating the hydroxyl group of the alkynol substrate.

**Scheme 1:**
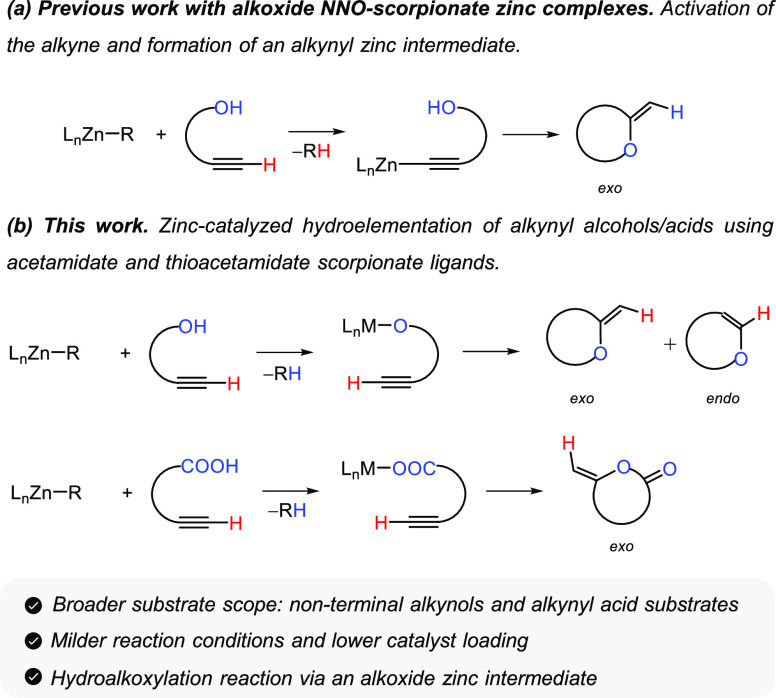
(a) Previous Zinc-Catalyzed Hydroalkoxylation of Alkynol Substrates and (b) This work: Zinc-Catalyzed Hydroelementation of Alkynol/Alkynoic Substrates

Despite progress in this field, the majority of hydroalkoxylation processes are catalyzed by highly expensive and less abundant metals. Therefore, the use of nonprecious metals has become highly desirable in terms of sustainability and environmental criteria.^23,24^ Although zinc has been widely studied for the synthesis of compounds by C–N and C–O bond formation reactions,^25^ there are scarce examples for the intramolecular hydroalkoxylation of alkynols. Recently, our research group has reported the first zinc catalysts for the hydroalkoxylation of different aromatic and aliphatic alkynyl alcohols, achieving high conversions under mild reaction conditions and 100% selectivity toward the cyclized *exo*-product (Scheme 1a).^26^

On the other hand, the hydrocarboxylation of alkynyl acid substrates has been less studied than their hydroalkoxylation analogs. This reaction yields various types of unsaturated lactones, which are prevalent structural motifs in natural and bioactive products, as well as useful compounds for the pharmaceutical and fragrance industries.^27^ Numerous transition metal catalysts have been reported for this process, especially iridium,^28^ rhodium,^29^ palladium,^30^ silver,^31^ and gold.^32^ The cycloisomerization reaction entails the π-activation of the C=C bond within the alkynoic acid. However, to the best of our knowledge, there are no precedents employing zinc catalysts for this reaction. Recently, Higashida et al. reported a *series* of gold–zinc catalysts for the 7-*exo-dig* hydrocarboxylation of nonactivated internal alkynes featuring flexible linker chains to produce ε-*exo*-alkylidene ε-lactones. The proximity between the gold and zinc sites was found crucial for high catalytic activity and cooperativity through a dual activation of the alkyne with a cationic gold atom and the carboxylic acid with a basic zinc salt.^32b^

Herein, we report the intramolecular hydroelementation reaction of a wide range of alkynyl alcohols and acid substrates catalyzed by novel acetamidate/thioacetamidate zinc complexes, becoming the first zinc catalysts reported for the cycloisomerization of alkynoic substrates. Kinetic and mechanistic studies have been performed for the hydroalkoxylation process, pointing to a mechanism through the activation of the substrate by the oxidizing element and the formation of a metal–alkoxide intermediate. The activation parameters have also been calculated and showed to be lower than those reported for previously used scorpionate zinc catalysts.

## Results and Discussion

### Synthesis and Structural Characterization

**Scheme 2:**
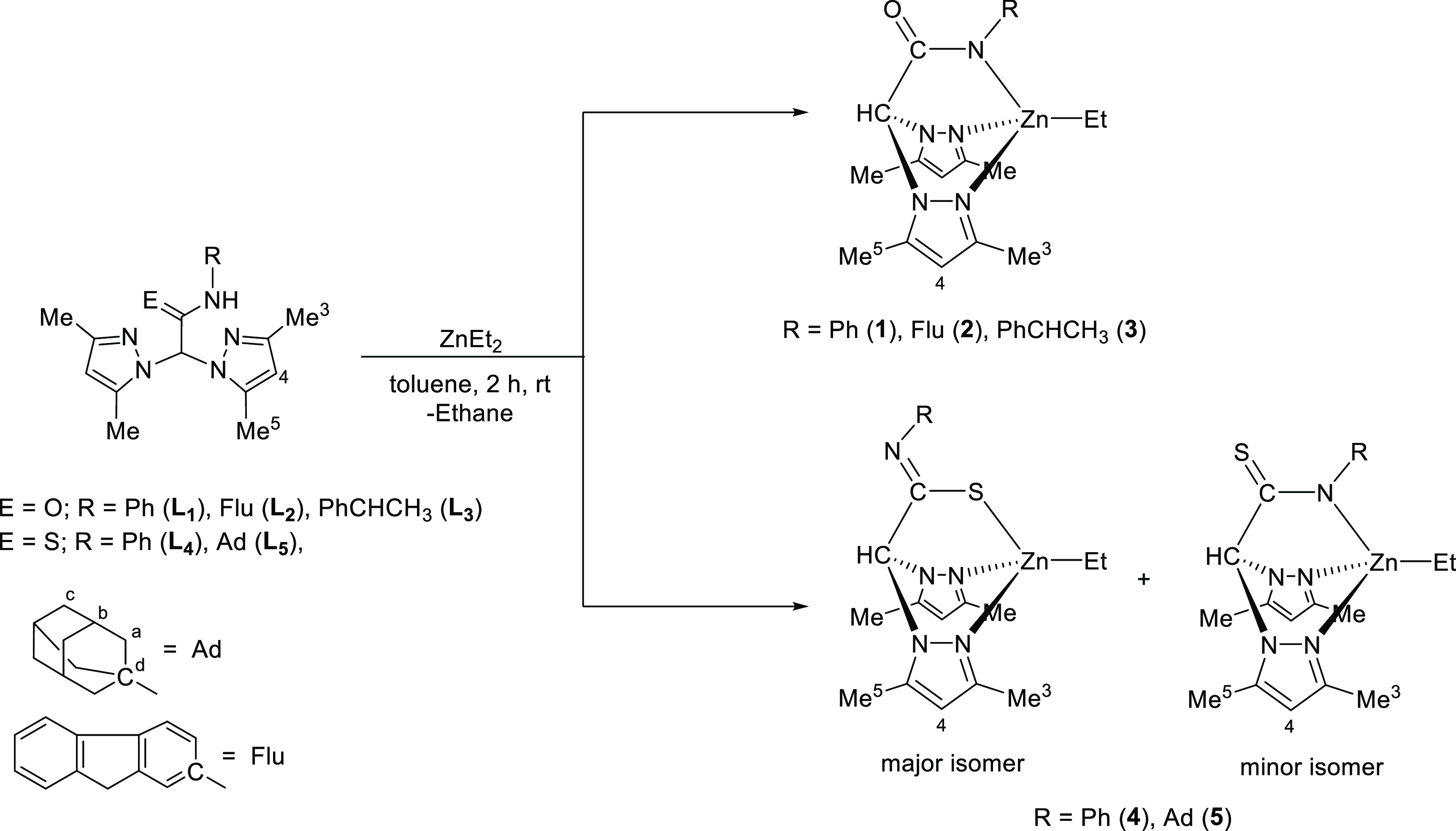
Synthesis of Zinc Complexes **1–5**

Heteroscorpionate acetamidate and thioacetamidate ligands have been thoroughly investigated and coordinated to a diverse range of metals (aluminum, rare-earth metals) due to their great versatility in their coordination mode.^33^ These ligands contain two Lewis basic coordinating groups (N and O/S centers), and this makes them of great interest in synthetic chemistry.^33^ Drawing upon this, we have now focused on the preparation of alkyl zinc complexes **1**–**5** by a reaction of the corresponding protonated acetamidate or thioacetamidate heteroscorpionate precursor [bpzpamH (**L**_**1**_), bpzfamH (**L**_**2**_), (*S*)-bpzmpamH (**L**_**3**_), bpzptamH (**L**_**4**_), and bpzatamH (**L**_**5**_)] with ZnEt_2_ in a 1:1 molar ratio in toluene at room temperature for 2 h. This reaction resulted in the formation of mononuclear ethyl zinc complexes [Zn(Et)(κ^3^-bpzpam)] (**1**), [Zn(Et)(κ^3^-bpzfam)] (**2**), [Zn(Et)(κ^3^-(*S*)-bpzmpam)] (**3**), [Zn(Et)(κ^3^-bpzptam)] (**4**), and [Zn(Et)(κ^3^-bpzatam)] (**5**) with vigorous elimination of ethane (Scheme 2). Complexes **1**–**5** are white solids and were obtained in excellent yields after the appropriate workup procedure. These compounds were obtained as achiral complexes except for compound **3**, which was isolated as an enantiomerically enriched compound. These complexes have shown to be stable against temperature and when they are exposed to air in solid state for several days.

Complexes **1**–**5** were structurally characterized by NMR spectroscopy and X-ray diffraction analysis. ^13^C{^1^H} NMR spectroscopy has been a great tool to deduce how the acetamidate–thioacetamide moiety of the ligand is coordinated to the zinc atom. In this sense, and in contrast to aluminum analogs,^33c,e^ acetamidate zinc complexes **1**–**3** exhibited a nitrogen coordination of the acetamidate moiety, as expected according to Pearson’s HSAB theory, in which the zinc atom displays an intermediate acidic hardness behavior.^34^ This was confirmed by the acetamidate carbon resonance, which exhibited a downfield shift compared to that of the neutral ligand (Table S1). On the other hand, thioacetamidate zinc complexes **4** and **5** showed two sets of signals for the thioacetamidate moiety in ^13^C{^1^H} NMR spectra. This represents a clear indication of the presence of two different isomers depending on the coordination mode of the thioacetamidate ligand (N or S) since both atoms display similar chemical hardness. The ratio of the isomer coordinated through the sulfur atom was found to be higher than that of its nitrogen counterpart for complex **5**, as the resonance assigned to the thioacetamidate carbon shifted to a higher field than that of the neutral ligand. This observation was ascribed to the steric hindrance of the ligand, which was shown to be higher for the adamantyl moiety in complex **5** than its phenyl analog **4**.

The ^1^H and ^13^C{^1^H} NMR spectra of zinc complexes **1**,**2**, **4**, and **5**, which contain nonchiral ligands, displayed a single set of resonances for the H^4^, Me^3^, and Me^5^ pyrazole protons, and these data confirm the equivalence of the two pyrazole rings (Figures S1–S5). On the contrary, complex **3**, which contains a stereogenic center in the heteroscorpionate ligand (*S*)-bpzmpam, revealed two different sets of signals for some of the protons and carbons at the 3, 4, and 5 positions of the pyrazole rings, indicating the nonequivalence between them (Figure S3). The spectroscopic data confirm a tetrahedral geometry around the zinc atom with the heteroscorpionate ligand linked in a monofacial tridentate form, κ^3^-NNN or κ^3^-NNS, and the ethyl ligand occupies the fourth position of the coordination sphere. ^1^H-NOESY-2D experiments enabled the clear assignment of all ^1^H resonances, while the assignment of the ^13^C{^1^H} NMR signals was accomplished using ^1^H–^13^C heteronuclear correlation (g-HSQC) experiments.

**Figure 1:**
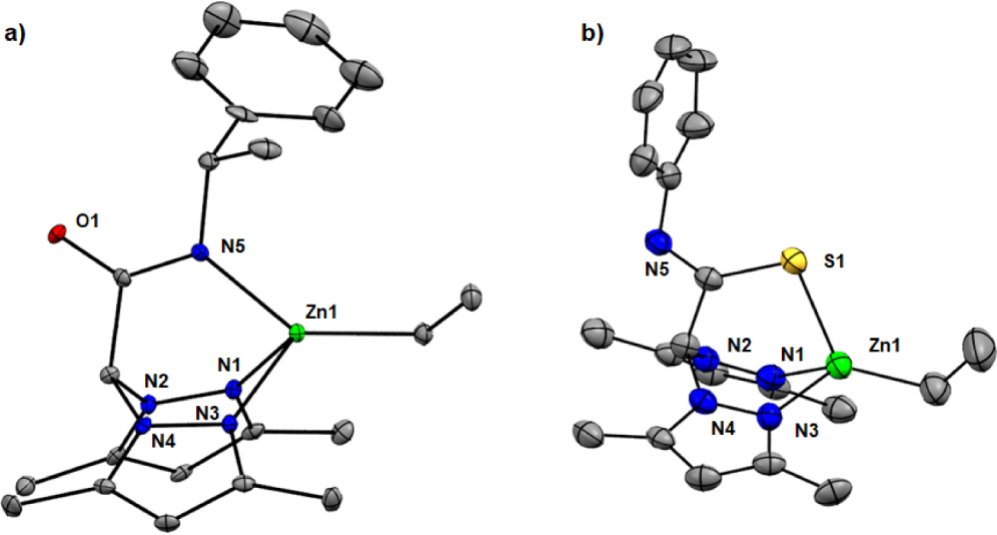
ORTEP diagrams for compounds **3** (a) and **4** (major isomer) (b).

The crystal structures of compounds **3** and **4** (major isomer) were corroborated by X-ray diffraction studies (Figure 1), and these structures are consistent with the solution proposed structures inferred from the spectroscopic data. These compounds exhibited a mononuclear structure with the ligand coordinated to the metal center through the nitrogen atom from the acetamidate group, the two nitrogen atoms of the pyrazole rings in a κ^3^-NNN monofacial tridentate mode for complex **3** (Figure 1a), and the sulfur atom of the thiacetamidate moiety in a κ^3^-NNS coordination fashion for complex **4** (major isomer) (Figure 1b). In addition, an ethyl group is bonded to the zinc center in complexes **3** and **4** (major isomer), occupying the fourth vacancy and completing the coordination sphere around the zinc atom.

Selected bond distances and angles, and the crystallographic data are provided in Tables S3 and S4, respectively. The Zn–C_ethyl_ [1.981(5) and 1.98(1) Å] and Zn–N_pyrazole_ [2.072(7)–2.143(4) Å] bond distances are consistent with those previously reported for alkyl zinc scorpionate compounds.^26,35^ The bond length Zn(1)–N(5) in complex **3** of 2.002(4) Å is shorter than Zn(1)–N(1) and Zn(1)–N(3) bond lengths of 2.143(4) and 2.109(4) Å, respectively, from the pyridinic nitrogen of both pyrazole rings, confirming the anionic fashion of this bond. The bond distance O(1)–C(14) of 1.248(6) Å for complex **3** confirms the localization of the double bond in the acetamidate moiety in this bond, which is similar to the C=O double bond theoretical distance of 1.220 Å.^36^ On the other hand, the bond distance N(5)–C(12) of 1.28(1) Å in complex **4** (major isomer) is very similar to that found for N=C double bond of 1.279 Å.^36^ The geometry at both zinc atoms is a distorted tetrahedral, with the most significant distortion observed in the N(3)–Zn(1)–N(1) bond angles for complexes **3** and **4** (major isomer), with values of 84.2(2)° and 85.6(3)°, respectively. Additionally, the dihedral angle between the C(1)–Zn(1)–N(5) and N(3)–Zn(1)–N(1) planes is 86.5° for complex **3**, and the dihedral angle between the C(19)–Zn(1)–S(1) and N(3)–Zn(1)–N(1) planes is 88.4° for complex **4** (major isomer), further confirming this distorted geometry.

### Catalytic Hydroalkoxylation/Hydrocarboxylation Studies

Following our previous methodology,^26^ compounds **1**–**5** were evaluated as catalysts in the hydroelementation reaction of alkynyl alcohols and alkynyl acid substrates. Initially, a first screening to evaluate the catalytic activity of compounds **1**–**5** was performed for the intramolecular hydroalkoxylation of 2-ethynylbenzyl alcohol (**6**) (Table 1). The reactions were conducted in a J-Young NMR tube at 80 °C in toluene-*d*_8_, utilizing a catalyst loading of 2.5 mol % (Table 1, entries 1–5). Acetamidate compounds (**1**–**3**) showed to be slightly more active than thioacetamide complexes (**4** and **5**), with complex **1** being the most active, achieving 92% conversion after 5 h (Table 1, entry 1; Figure S6). In terms of selectivity, it is worth noting that all complexes were 100% selective toward the hydroalkoxylation of substrate **6**, forming the *exo*-cyclic enol ether **7** with no evidence of the alternative 6-*endo*-cyclization. This result aligns well with previously reported findings for other zinc complexes.^26^

**Table 1:**
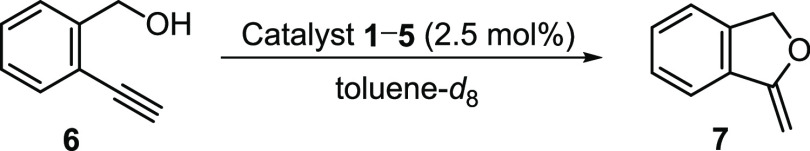
Hydroalkoxylation/Cyclization Reaction of 2-Ethynylbenzyl Alcohol (**6**) by Catalysts **1**–**5**^a^

entry	catalyst	conv. (%)^b^	TOF (h^–1^)
1	1	93	7.4
2	2	76	6.0
3	3	65	5.2
4	4	77	6.2
5	5	51	4.1

a Reaction conditions: 0.5 mmol substrate, 0.0125 mmol catalyst, 0.6 mL toluene-*d*_8_, 80 °C, 5 h. Determined by ^1^H NMR.

b TOF = (mol of product/mol of cat. × time).

After having optimized the reaction conditions for the hydroalkoxylation of 2-ethynylbenzyl alcohol (**6**), we investigated the substrate scope and studied the cyclization of different aliphatic alkynyl alcohols and acids (Table 2, entries 1–8; Figures S7–S11). These substrates proved to be more challenging than compound **6**. Thus, harsher reaction conditions were employed, increasing the catalyst loading and reaction temperature. As it can be observed, there is a strong dependence between the conversion and the structure for these substrates, since they are not aligned to form the cyclized product. These results evidence the influence of the Thorpe–Ingold effect^37,38^ of the geminal substituents with respect to the alcohol/carboxylic acid group and the electronic effects of the aromatic substituents in the hydroelementation reaction of these substrates. In terms of selectivity, it is worth noting that only the hydroalkoxylation of 4-pentynol (**8**) afforded the formation of both cyclization products, the *exo*-furane **8′** and *endo*-2-pyrene **8″** derivatives in a 67:33 ratio. All other substrates were 100% selective toward the formation of the *exo*-cyclic product. In the hydrocarboxylation of substrates **10** and **11**, unsaturated *exo*-lactones **10′** and **11′** were obtained. Lactone **11′** was obtained in a lower yield than its **10′** analogs, due to the lower stability of the 6-membered ring in comparison to the 5-membered one.

**Table 2:**
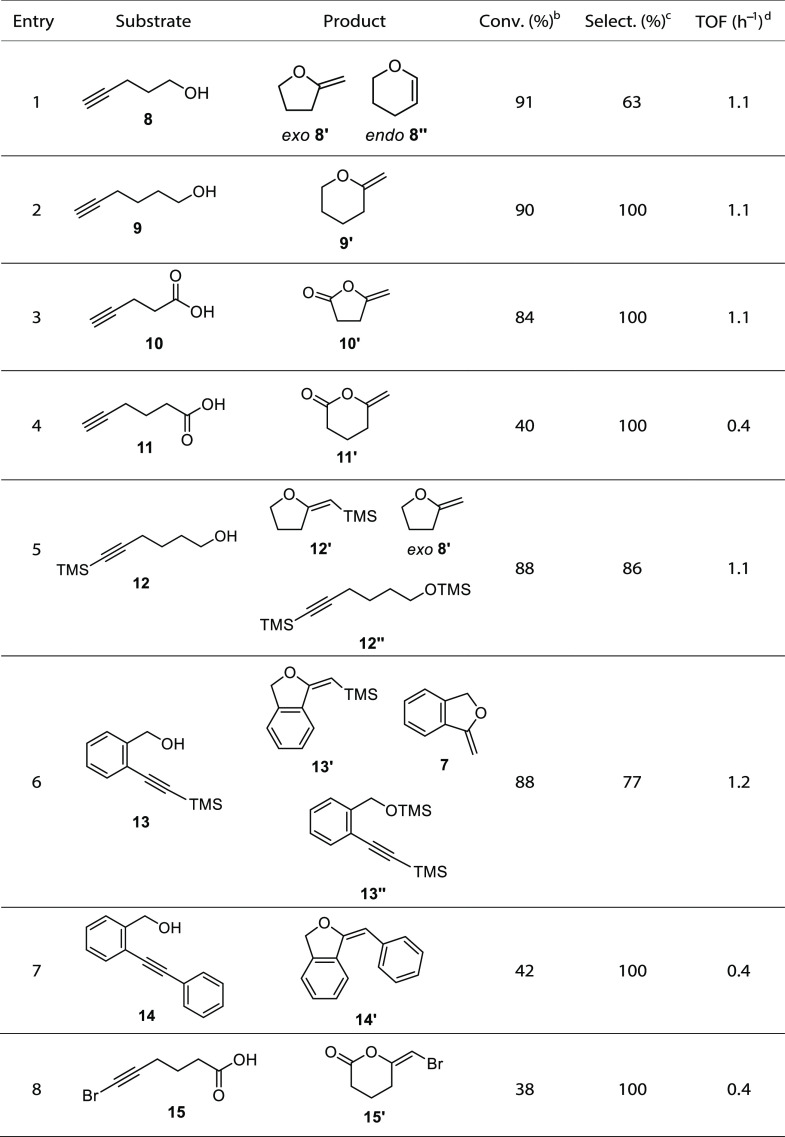
Cyclization Reaction of Alkynyl Alcohols/Carboxylic Acids Catalyzed by Compound 1^a^

a Reaction conditions: 0.5 mmol substrate, 0.025 mmol catalyst, 0.6 mL toluene-*d*_8_, 90 °C, 16 h.

b Determined by ^1^H NMR.

c Selectivity to the *exo*-product.

d TOF = (mol of product/mol of cat. × time).

Finally, to demonstrate the functional group tolerance of complex **1**, the catalytic cyclization of some internal alkyne derivatives was evaluated (Table 2, entries 5–8; Figures S12–S14). As can be observed, catalyst **1** proved to be very efficient in the catalytic intramolecular hydroalkoxylation of SiMe_3_-substituted substrates **12** and **13**, achieving conversion rates of 88%. It is also noteworthy that the desired cyclized products **12′** and **13′** were obtained with high selectivity despite the formation of the corresponding SiMe_3_-protected starting materials and SiMe_3_-deprotected products. The substitution of the trimethylsilyl moiety for a phenyl group in the alkyne function (substrate **14**) led to a significant decrease in the conversion rate (42%) compared to **13**, consistent with the previously reported one.^22f,h^ On the other hand, the cyclization of the internal alkynyl acid **15** using compound **1** as a catalyst was also successful, achieving a conversion similar to that obtained for the analogous nonterminal substrate **11**.

### Kinetic and Mechanistic Studies

**Figure 2:**
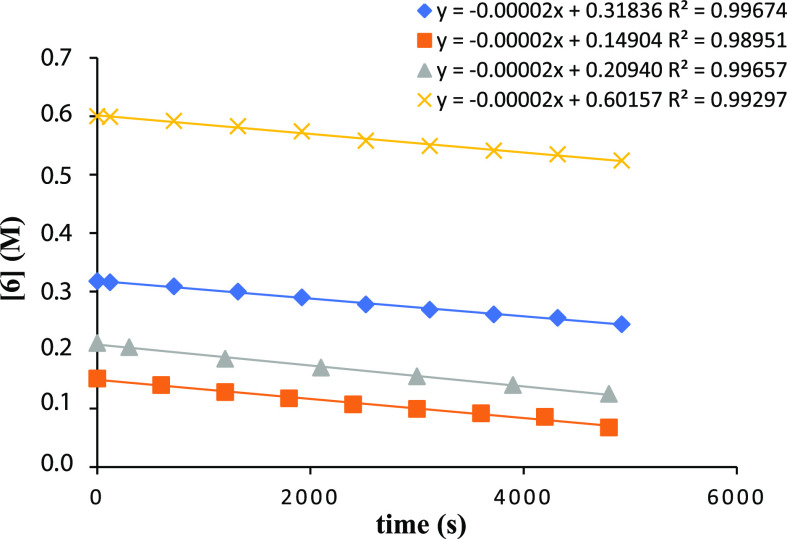
Plot of [**6**] versus reaction time for the hydroalkoxylation of **6** catalyzed by catalyst **1** at various initial concentrations of **6**.

Kinetic studies were performed using 2-ethynylbenzyl alcohol (**6**) as a substrate and alkyl zinc complex **1** as a catalyst. The essays were performed in a J-Young NMR tube at 80 °C in toluene-*d*_8_ and the reaction was monitored by ^1^H NMR spectroscopy (Figure S17). The order of the reaction with respect to substrate **6** was studied first, and this intramolecular hydroalkoxylation reaction was carried out at four different concentrations of alkynyl alcohol **6** (0.035–0.600 M), while maintaining a constant catalyst **1** concentration of 0.007 M. The linear correlation between [**6**] and time indicated that the reaction is zero-order with respect to the substrate concentration (Figure 2).

**Figure 3:**
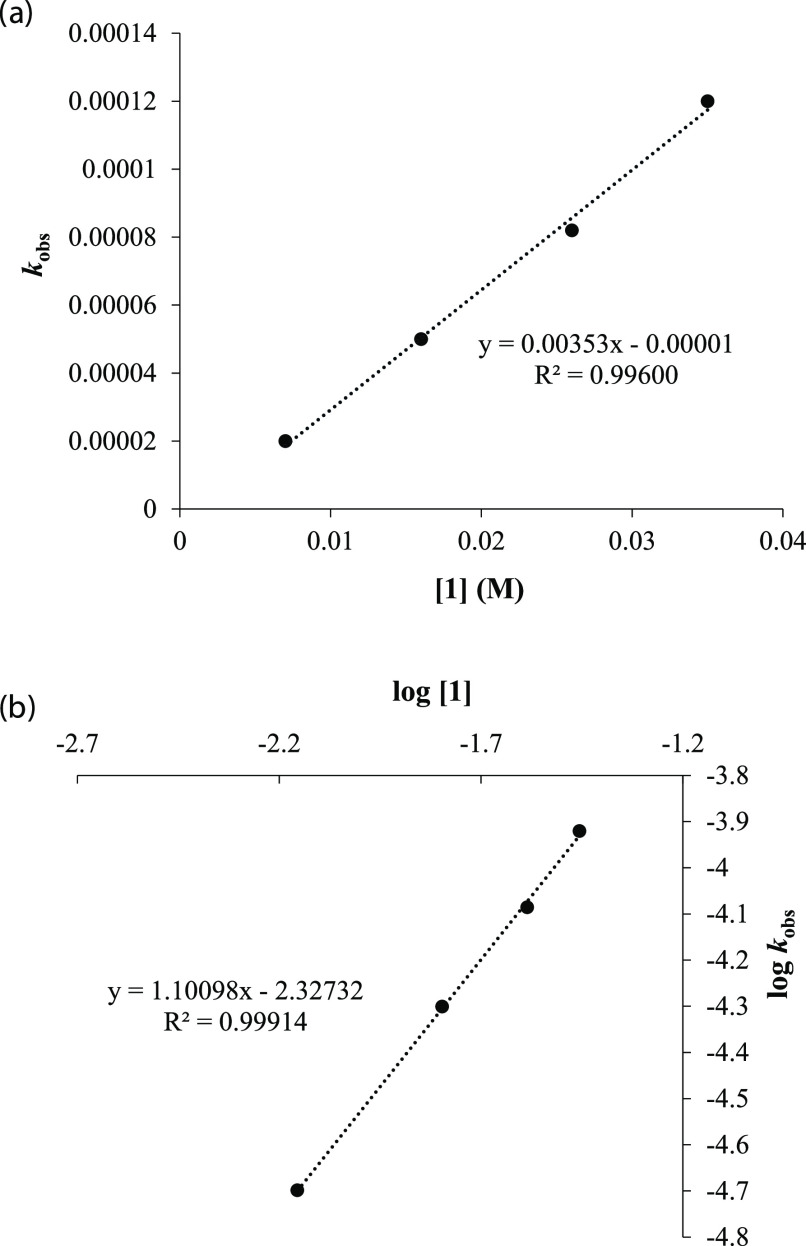
(a) Plot of *k*_obs_ versus [**1**] for the hydroalkoxylation of **6**. (b) Plot of log *k*_obs_ versus log [**1**].

To determine the order with respect to zinc compound **1**, the same procedure was followed, and the cyclization reaction of substrate **6** was carried out at four different concentrations of complex **1** (0.007–0.035 M), while maintaining a constant concentration of substrate **6** of 0.600 M (Figure S18). As it can be observed, as the catalyst concentration increases, so does the reaction rate for the hydroalkoxylation process. The plot of *k*_obs_ versus [**1**] displayed a strong linear fit, suggesting that the reactions follow first-order kinetics with respect to catalyst concentration (Figure 3a). This result was further supported by plotting log [**1**] against log *k*_obs_, revealing a slope of 1.101 (Figure 3b).

**Figure 4:**
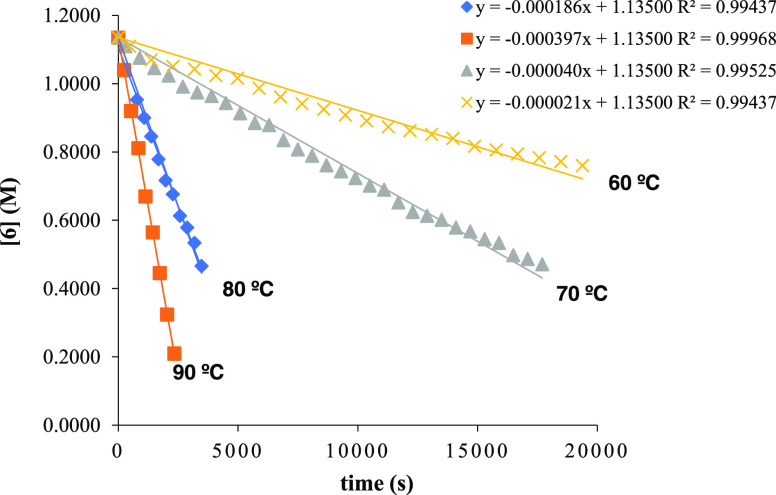
Plot of substrate consumption over time during the hydroalkoxylation of **6** by catalyst **1** at varying temperatures.

Having established the equation rate for the hydroalkoxylation process and the order with respect to substrate **6** and catalyst **1**, a kinetic study at variable temperatures was conducted to determine the activation parameters. Reactions were performed at four temperatures ranging from 60 to 90 °C, and their progress was monitored by using ^1^H NMR spectroscopy. Linear plots in all cases supported that reactions proceeded with zero-order kinetics with respect to the concentration of substrate **6** (Figure 4). Standard Arrhenius and Eyring plots were utilized to calculate the activation parameters (Figures S19 and S20, respectively). The activation energy (*E*_a_), enthalpy (Δ*H*^⧧^), and entropy (Δ*S*^⧧^) were determined to be 24.5(±0.2) kcal/mol, 23.8(±0.2) kcal/mol, and −12.5(±0.2) eu, respectively. The values of *E*_a_ and Δ*H*^⧧^ for catalyst **1** closely resemble those reported previously for other scorpionate zinc complexes.^26^

**Figure 5:**
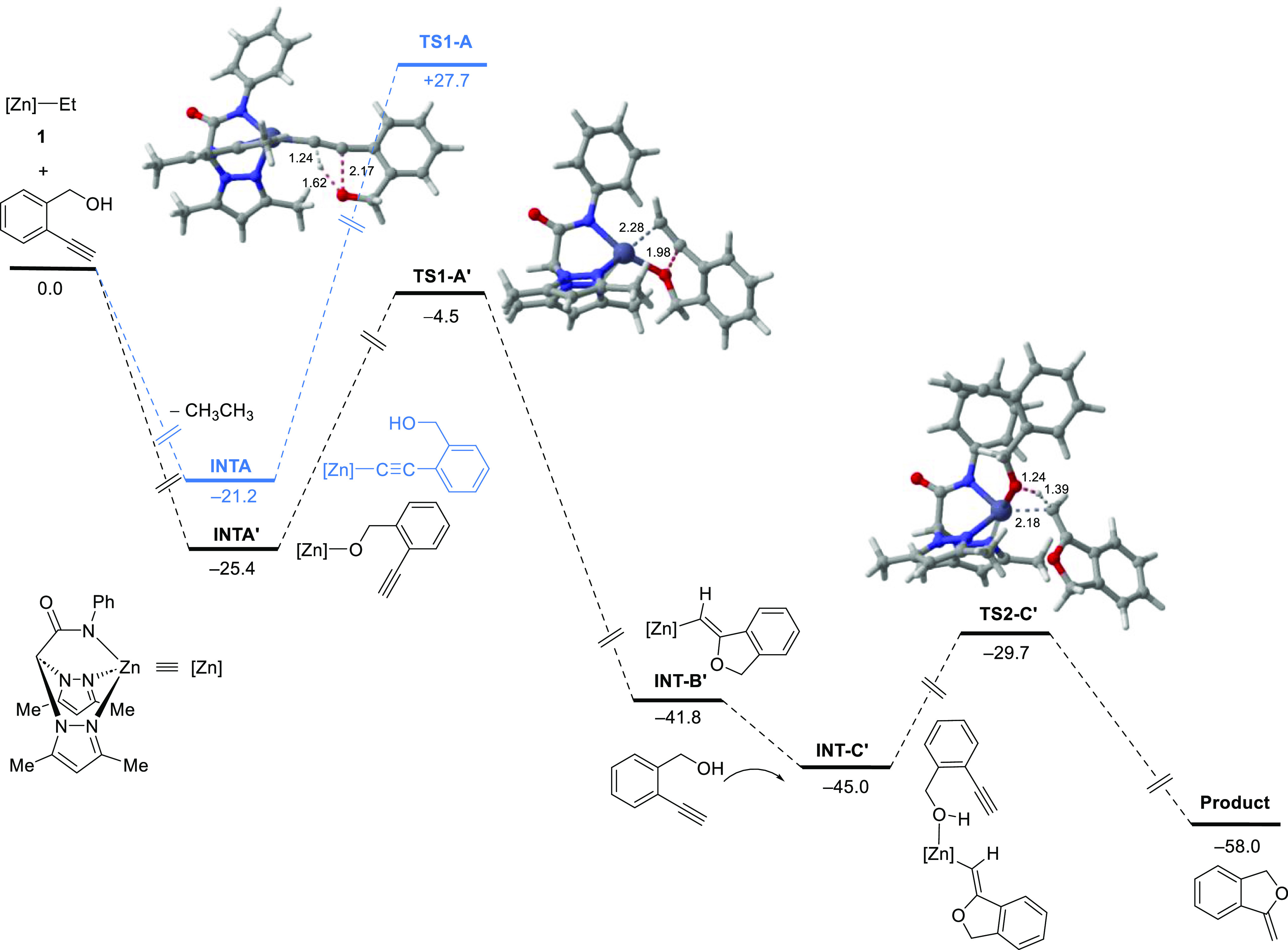
Gibbs free energy profile (in kcal/mol) for zinc-catalyzed hydroalkoxylation of **6**. Computed at the PCM-(toluene)-B3LYP-D3/def2-SVP level. Black lines represent the more plausible catalytic route. Blue lines represent alternative reaction paths to the catalytic cycle that are not favorable.

With the aim of obtaining a better understanding of the hydroalkoxylation mechanism, the stoichiometric reaction of complex **1** with 2-ethynylbenzyl alcohol (**6**) was explored. Interestingly, and in contrast to what was previously observed with other scorpionate zinc complexes,^26^ the latter reaction afforded a mixture of unidentified reaction intermediates and *exo*-cyclic product **7** even under mild conditions. This result prompted the investigation of the mechanism in more detail. To this end, density functional theory (DFT) calculations were performed at the dispersion-corrected PCM-(toluene)-B3LYP-D3/def2-SVP level (see computational details in the Supporting Information). The overall free energy profiles for the catalytic alkyne hydroalkoxylation are illustrated in Figure 5.

As depicted in Figure 5, this reaction initiates with the deprotonation of the alkyne or the hydroxyl group of substrate **6** via alkane elimination, forming the corresponding alkynyl (**INT-A**) or the alkoxide intermediate (**INT-A′**). Notably, the formation of the alkoxide intermediate is slightly more exergonic (approximately 4.2 kcal/mol) than that of the alternative alkynyl activation. Subsequently, a concerted coupling reaction between the alkyne group and the Zn–O moiety occurs via the transition state **TS1-A′** (Δ*G*^⧧^ = 20.9 kcal/mol), which is kinetically more favored over the alternative nucleophilic attack of the hydroxyl on the alkynyl–Zn bond. In addition, the latter pathway via transition state **TS1-A** appears unfeasible due to its much higher computed barrier (Δ*G*^⧧^ = 48.9 kcal/mol). Therefore, the catalytic cycle operates through the formation of the alkoxide intermediate (**INT-A′**) to yield the cyclized product (**INT-B′**), releasing 16.4 kcal/mol (compared to **INT-A′**). The cyclization step possibly serves as the rate-determining step along this pathway.

Moreover, the calculations reveal the release of a slight amount of free energy after a second substrate molecule coordinates to the metal center, forming **INT-C′**. Finally, an exergonic proton transfer (Δ*G* = −13.0 kcal/mol) from the hydroxyl group of the coordinated substrate to the cyclized product is observed via transition state **TS2-C′**. The cyclized product is then removed, and a new substrate molecule is incorporated, restoring the alkoxide intermediate **INT-A′**.

## Conclusions

Novel zinc acetamide/thioacetamidate scorpionate compounds were synthesized and characterized. These complexes were evaluated as catalysts in the cyclization reaction of alkynyl alcohol/acid substrates, achieving good conversions under milder reaction conditions and catalyst loadings lower than those reported previously. Furthermore, to the best of our knowledge, this represents the first use of zinc catalysts for the hydroalkoxylation of nonterminal alkynyl alcohols and the hydrocarboxylation of alkynyl acid substrates. The cyclization process has shown to be fully selective toward the *exo*-product in most of the cases. Kinetic studies have shown a zero-order dependence on substrate concentration and first-order dependence with respect to catalyst concentration. Finally, DFT calculations agree with the formation of an alkoxide intermediate as the catalytically active species and have pointed to the C≡C bond insertion to the alkoxide intermediate as the rate-determining step of the reaction. Additional investigations are underway to broaden the range of substrates in intermolecular hydroalkoxylation/hydrocarboxylation processes.

## Experimental Section

All handling of air- and moisture-sensitive compounds was conducted under dry nitrogen using either a Braun Labmaster glovebox or standard Schlenk line techniques. NMR spectra were acquired on a Bruker Ascend TM-500 spectrometer and referenced to the residual deuterated solvent. Elemental analyses were performed using a PerkinElmer 2400 CHN analyzer. Solvents were predried over sodium wire (toluene, THF, *n*-hexane) and distilled under nitrogen from sodium (toluene, THF) or sodium–potassium alloy (*n*-hexane). Deuterated solvents were stored over activated 4 Å molecular sieves and degassed by several freeze–thaw cycles. 2-Ethynylbenzyl alcohol (**6**), 4-pentynol (**8**), 5-hexynol (**9**), 5-(trimethylsilyl)-4-pentynol (**10**), 5-pentenoic acid (**11**), and 5-hexenoic acid (**12**) were purchased from Sigma-Aldrich. Substrates (2-((trimethylsilyl)ethynyl)phenyl)methanol (**13**), (2-(phenylethynyl)phenyl)methanol (**14**), and 6-bromo-5-hexynoic acid (**15**) were synthesized as previously described in the literature.^22f,39^ All other reagents were procured from standard commercial suppliers and used without further purification. Safety statements: No uncommon hazards are noted.

### Synthesis of [Zn(Et)(κ^3^-bpzpam)] (**1**)

In a 100 mL Schlenk tube, bpzpamH (**L**_**1**_) (0.50 g, 1.55 mmol) was dissolved in dry toluene (25 mL) and cooled to −50 °C. Then, a solution of ZnEt_2_ (1 M in *n*-hexane, 1.55 mL, 1.55 mmol) was added, and the mixture was allowed to warm up and stirred for 1 h. After that time, the solvent was removed under reduced pressure, and the residue was washed with *n*-hexane to afford complex **1** as a white solid. Yield: 0.61 g (95%). Anal. Calcd for C_20_H_25_N_5_OZn: C, 57.6; H, 6.1; N, 16.8. Found: C, 57.8; H, 6.3; N, 16.4. ^1^H NMR (500 MHz, C_6_D_6_, 297 K): δ 8.18 (d, *J*_HH_ = 7.0 Hz, 2H, ^*o*^*H-*Ar), 7.34 (m, *J*_HH_ = 7.0 Hz, 2H, ^*m*^*H-*Ar), 6.98 (t, *J*_HH_ = 7.0 Hz, 1H, ^*p*^*H-*Ar), 6.74 (s, 1H, CH), 5.22 (s, 2H, H^4^), 1.99 (s, 6H, Me^3^), 1.82 (t, *J*_HH_ = 8.1 Hz, 3H, ZnCH_2_CH_3_), 1.79 (s, 6H, Me^5^), 1.10 (m, *J*_HH_ = 8.1 Hz, 2H, Zn*CH*_*2*_CH_3_). ^13^C{^1^H} NMR (125 MHz, C_6_D_6_, 297 K): 164.6 (N*CO*), 149.8; 140.6 (C^3^, C^5^), 147.9 (*C*^*i*^-Ar), 128.1 (*C*^*m*^-Ar), 124.4 (C*^p^*-NAr), 122.4 (*C*°-NAr), 106.1 (C^4^), 71.1 (CH), 13.5 (ZnCH_2_*CH*_*3*_), 12.3 (Me^3^), 10.0 (Me^5^), 1.4 (Zn*CH*_*2*_CH_3_).

### Synthesis of [Zn(Et)(κ^3^-bpzfam)] (**2**)

The synthesis of **2** was performed by following the same procedure as that for compound **1**, using bpzfamH (**L**_**2**_) (0.50 g, 1.22 mmol) and ZnEt_2_ (1 M in *n*-hexane, 1.22 mL, 1.22 mmol). Compound **2** was isolated as a white solid. Yield: 0.55 g (90%). Anal. Calcd for C_27_H_29_N_5_OZn: C, 64.2; H, 5.8; N, 13.9. Found: C, 64.3; H, 5.9; N, 13.7 ^1^H NMR (500 MHz, C_6_D_6_, 297 K): δ 8.32–7.00 (m, 7H, *Ar-*Flu), 6.67 (s, 1H, CH), 5.21 (s, 2H, H^4^), 3.56 (s, 2H, *CH*_*2*_-Flu), 1.96 (s, 6H, Me^3^), 1.86 (t, *J*_HH_ = 8.1 Hz, 3H, ZnCH_2_*CH*_*3*_), 1.77 (s, 6H, Me^5^), 1.04 (m, *J*_HH_ = 8.1 Hz, 2H, Zn*CH*_*2*_CH_3_). ^13^C{^1^H} NMR (125 MHz, C_6_D_6_, 297 K): 164.5 (N*CO*), 149.5; 140.9 (C^3^, C^5^), 129.9–119.9 (*Ar*-Flu), 106.2 (C^4^), 71.2 (CH), 36.8 (*CH*_*2*_-Flu), 13.0 (ZnCH_2_*CH*_*3*_), 12.5 (Me^3^), 10.0 (Me^5^), −2.0 (Zn*CH*_*2*_CH_3_).

### Synthesis of [Zn(Et)(κ^3^-(*S*)-bpzmpam)] (**3**)

The synthesis of **3** was conducted by following the same procedure as that for compound **1** using (*S*)-bpzmpamH (**L**_**3**_) (0.50 g, 1.42 mmol) and ZnEt_2_ (1 M in *n*-hexane, 1.42 mL, 1.42 mmol). Compound **3** was isolated as a white solid. Yield: 0.59 g (94%). Anal. Calcd for C_22_H_29_N_5_OZn: C, 59.4; H, 6.6; N, 15.7. Found: C, 59.6; H, 6.8; N, 15.4 ^1^H NMR (500 MHz, C_6_D_6_, 297 K): δ 7.49 (d, *J*_HH_ = 7.0 Hz, 2H, ^*o*^*H-*Ar), 7.20 (m, *J*_HH_ = 7.0 Hz, 2H, ^*m*^*H-*Ar), 7.02 (t, *J*_HH_ = 7.0 Hz, 1H, ^*p*^*H-*Ar), 6.99 (brs, 1H, CH), 5.74 (m, 1H, *CH*MePh), 5.25; 5.22 (s, 2H, H^4,4′^), 2.07; 2.06 (s, 6H, Me^3,3′^), 1.75 (brs, 3H, CH*Me*Ph), 1.70 (t, *J*_HH_ = 8.1 Hz, 3H, ZnCH_2_*CH*_*3*_), 1.65; 1.62 (s, 6H, Me^5,5′^), 0.65 (m, *J*_HH_ = 8.1 Hz, 2H, Zn*CH*_*2*_CH_3_). ^13^C{^1^H} NMR (125 MHz, C_6_D_6_, 297 K): 165.8 (N*CO*), 149.0; 148.8; 148.2; 140,8; 140.6 (C^3,3′,5,5′^ and *C*^*i*^-Ar), 127.9 (*C*^*m*^-Ar), 126.6 (C*^p^*-Ar), 125.8 (*C*°-Ar), 105.9; 105.8 (C^4,4′^), 70.0 (*CH*), 52.0 (*C*HMePh), 24.0 (CH*Me*Ph), 13.5 (ZnCH_2_*CH*_*3*_), 12.3; 12.2 (Me^3,3′^), 10.1; 10.0 (Me^5,5′^), −1.8 (Zn*CH*_*2*_CH_3_).

### Synthesis of [Zn(Et)(κ^3^-bpzptam)] (**4**)

The synthesis of **4** was conducted by following the same procedure as that for compound **1** using bpzptamH (**L**_**4**_) (0.50 g, 1.48 mmol) and ZnEt_2_ (1 M in *n*-hexane, 1.48 mL, 1.48 mmol). Compound **4** was isolated as a white solid. Yield: 0.61 g (95%). Anal. Calcd for C_20_H_25_N_5_SZn: C, 55.5; H, 5.8; N, 16.2. Found: C, 55.7; H, 5.9; N, 16.0 ^1^H NMR (500 MHz, C_6_D_6_, 297 K): Major isomer (κ^3^-NNS): δ 7.40 (d, *J*_HH_ = 7.0 Hz, 2H, ^*o*^*H-*Ar), 7.22 (m, *J*_HH_ = 7.0 Hz, 2H, ^*m*^*H-*Ar), 7.01 (s, 1H, CH), 6.95 (t, *J*_HH_ = 7.0 Hz, 1H, ^*p*^*H-*Ar), 5.31 (s, 2H, H^4^), 2.05 (s, 6H, Me^3^), 1.86 (s, 6H, Me^5^), 1.84 (t, *J*_HH_ = 8.1 Hz, 3H, ZnCH_2_*CH*_*3*_), 1.00 (m, *J*_HH_ = 8.1 Hz, 2H, Zn*CH*_*2*_CH_3_). ^13^C{^1^H} NMR (125 MHz, C_6_D_6_, 297 K): 171.7 (N*CS*), 149.9; 141.1 (C^3^, C^5^), 151.8 (*C*^*i*^-Ar), 128.8 (*C*^*m*^-Ar), 123.9 (C*^p^*-NAr), 122.3 (*C*°-NAr), 106.6 (C^4^), 74.3 (CH), 13.9 (ZnCH_2_*CH*_*3*_), 12.9 (Me^3^), 10.6 (Me^5^), −1.2 (Zn*CH*_*2*_CH_3_). Minor isomer (κ^3^-NNN): δ 7.66 (d, *J*_HH_ = 8.5 Hz, 2H, ^*o*^*H-*Ar), 7.54 (s, 1H, *CH*), 7.23 (m, *J*_HH_ = 8.5 Hz, 2H, ^*m*^*H-*Ar), 6.95 (t, *J*_HH_ = 8.5 Hz, 1H, ^*p*^*H-*Ar), 5.26 (s, 2H, H^4^), 1.97 (s, 6H, Me^3^), 1.94 (s, 6H, Me^5^), 1.65 (t, *J*_HH_ = 8.1 Hz, 3H, ZnCH_2_*CH*_*3*_), 0.85 (m, *J*_HH_ = 8.1 Hz, 2H, Zn*CH*_*2*_CH_3_). ^13^C{^1^H} NMR (125 MHz, C_6_D_6_, 297 K): 187.2 (*NC*S), 149.9; 141.8 (C^3^, C^5^), 150.1 (*C*^*i*^-Ar), 128.6 (*C*^*m*^-Ar), 125.0 (C*^p^*-Ar), 124.6 (*C*°-Ar), 105.4 (C^4^), 77.5 (CH), 13.5 (ZnCH_2_CH_3_), 12.8 (Me^3^), 10.7 (Me^5^), −2.7 (ZnCH_*2*_CH_3_).

### Synthesis of [Zn(Et)(*κ*^3^-bpzatam)] (**5**)

The synthesis of **5** was performed by following the same procedure as that for compound **1** using bpzatamH (**L**_**5**_) (0.50 g, 1.26 mmol) and ZnEt_2_ (1 M in *n*-hexane, 1.26 mL, 1.26 mmol). Compound **5** was isolated as a white solid. Yield: 0.53 g (85%). Anal. Calcd for C_24_H_35_N_5_SZn: C, 58.7; H, 7.2; N, 14.3. Found: C, 58.9; H, 7.5; N, 14.0. Major isomer (κ^3^-NNS): ^1^H NMR (500 MHz, C_6_D_6_, 297 K): δ 6.83 (s, 1H, CH), 5.31 (s, 2H, H^4^), 2.51 (brs, 6H, CH_2_a), 2.07 (m, 3H, CHb), 2.04 (s, 6H, Me^3^), 1.90 (t, *J*_HH_ = 8.1 Hz, 3H, ZnCH_2_*CH*_*3*_), 1.88 (s, 6H, Me^5^), 1.69 (m, 6H, CH_2_c), 1.03 (m, *J*_HH_ = 8.1 Hz, 2H, Zn*CH*_*2*_CH_3_). ^13^C{^1^H} NMR (125 MHz, C_6_D_6_, 297 K): 164.1 (N*CS*), 149.0; 140.5 (C^3^, C^5^), 106.0 (C^4^), 76.0 (CH), 57.0–30.1 (Ad), 13.7 (ZnCH_2_*CH*_*3*_), 12.6 (Me^3^), 10.4 (Me^5^), −1.6 (Zn*CH*_*2*_CH_3_). Minor isomer (κ^3^-NNN): ^1^H NMR (500 MHz, C_6_D_6_, 297 K): δ 7.00 (s, 1H, CH), 5.46; 5.24 (s, 2H, H^4,4′^), 2.78; 2.60; 2.51 (brs, 6H, CH_2_a), 2.07 (s, 3H, CHb), 2.04 (s, 6H, Me^3^), 1.94 (s, 6H, Me^5^), 1.83 (m, 3H, ZnCH_2_*CH*_*3*_), 1.72–1.50 (m, 6H, CH_2_c), 0.91 (m, 2H, Zn*CH*_*2*_CH_3_). ^13^C{^1^H} NMR (125 MHz, C_6_D_6_, 297 K): 184.9 (N*CS*), 149.0; 141.1 (C^3^, C^5^), 106.2 (C^4,4′^), 79.7 (CH), 58.4–29.0 (Ad), 13.3 (ZnCH_2_*CH*_*3*_), 12.4 (Me^3^), 10.8 (Me^5^), 1.3 (ZnCH_*2*_CH_3_).

## Supporting Information Available

The synthesis of complexes **1**–**5**, catalytic intramolecular hydroalkoxylation, spectra of ^1^H and ^13^C{^1^H} NMR for complexes **1**–**5**, kinetic measurements and representative kinetic plots, and X-ray crystallographic data for compounds **3** and **4** (major isomer) (PDF)
