# Supplementary material for: Zinc-Catalyzed Cyclization of Alkynyl Derivatives: Substrate Scope and Mechanistic Insights
Source: Inorg Chem. 2024 Jul 16;63(30):13875–85. doi: 10.1021/acs.inorgchem.4c00832 (PMC11289758; doi:10.1021/acs.inorgchem.4c00832)
Supplement: Supplementary file 1 — ic4c00832_si_001.pdf [file ic4c00832_si_001.pdf]

SUPPORTING INFORMATION FOR:

Zinc-Catalyzed Cyclization of Alkynyl-Derivatives: Substrate Scope and Mechanistic Insights

Marc Martínez de Sarasa Buchaca,[a] Miguel A. Gaona,[a] Luis F. Sánchez-Barba,[c] Andrés Garcés,[c] Ana M. Rodríguez,[a] Antonio Rodríguez-Diéguez,[d] Felipe de la Cruz-Martínez,[,b] José A. Castro-Osma,*[,b] Agustín Lara-Sánchez*[,a]*

[a]Universidad de Castilla-La Mancha, Departamento de Química Inorgánica, Orgánica y Bioquímica-Centro de Innovación en Química Avanzada (ORFEO-CINQA), Facultad de Ciencias y Tecnologías Químicas, 13071-Ciudad Real, Spain. E-mail: Agustin.Lara@uclm.es.

[b]Universidad de Castilla-La Mancha, Departamento de Química Inorgánica, Orgánica y Bioquímica-Centro de Innovación en Química Avanzada (ORFEO-CINQA), Facultad de Farmacia, 02071-Albacete, Spain. E-mail: Felipe.Cruz@uclm.es; JoseAntonio.Castro@uclm.es.

[c]Universidad Rey Juan Carlos, Departamento de Biología y Geología, Física y Química Inorgánica, 28933-Móstoles, Spain.

[d]Universidad de Granada, Departamento de Química Inorgánica, Facultad de Ciencias, 18071-Granada, Spain

Table of Contents

1. Experimental section **S4**

2. Figures and Tables referred to in main text **S9**

Table S1. NCO C^{13}-NMR chemical shifts for complexes **1-3** and neutral heteroscorpionate ligand precursors in C_6D_6 S9

Table S2. NCS C^{13}-NMR chemical shifts for complexes **1-3** and neutral heteroscorpionate ligand precursors in C_6D_6 S9

Figure S1. NMR spectra for [[Zn{Et}(κ^3-bpzpam)] (**1**) in C_6D_6 S10

Figure S2. NMR spectra for [Zn{Et}(κ^3-bpzfam)] (**2**) in C_6D_6 S12

Figure S3. NMR spectra for [Zn{Et}(κ^3-(*S*)-bpzmpam)] (**3**) in C_6D_6 S14

Figure S4. NMR spectra for [Zn{Et}(κ^3-bpzptam)] (**4**) in C_6D_6 S16

Figure S5. NMR spectra for [Zn{Et}(κ^3-bpzatam)] (**5**) in C_6D_6 S18

Figure S6. NMR crude (500 MHz, toluene-d_8, 25 ºC) for the reaction of substrate **6** to afford cyclized product **7** using catalyst **1** (Table 1, entry 1) S20

Figure S7. NMR crude (500 MHz, toluene-d_8, 25 ºC) for the reaction of substrate **8** to afford cyclized products **8’** and **8’’** using catalyst **1** (Table 2, entry 1) S20

Figure S8. NMR crude (500 MHz, toluene-d_8, 25 ºC) for the reaction of substrate **9** to afford cyclized product **9’** using catalyst **1** (Table 2, entry 2) S21

Figure S9. NMR crude (500 MHz, toluene-d_8, 25 ºC) for the reaction of substrate **10** to afford cyclized product **10’** using catalyst **1** (Table 2, entry 3) S21

Figure S10. NMR crude (500 MHz, toluene-d_8, 25 ºC) for the reaction of substrate **11** to afford cyclized product **11’** using catalyst **1** (Table 2, entry 4) S22

Figure S11. NMR crude (500 MHz, toluene-d_8, 25 ºC) for the reaction of substrate **12** to afford cyclized product **12’** using catalyst **1** (Table 2, entry 5) S22

Figure S12. NMR crude (500 MHz, toluene-d_8, 25 ºC) for the reaction of substrate **13** to afford cyclized product **13’** using catalyst **1** (Table 2, entry 6) S23

Figure S13. NMR crude (500 MHz, toluene-d_8, 25 ºC) for the reaction of substrate **14** to afford cyclized product **14’** using catalyst **1** (Table 2, entry 7) S23

Figure S14. NMR crude (500 MHz, toluene-d_8, 25 ºC) for the reaction of substrate **15** to afford cyclized product **15’** using catalyst **1** (Table 2, entry 8) S24

Figure S15. NOESY-1D experiment performed for cyclized product **13'** S24

Figure S16. NOESY-1D experiment performed for cyclized product **14'** S25

Figure S17. ^{1}H NMR monitoring of the hydroalkoxylation of **6** to **7** from t=0 to t=2.5 h mediated by catalyst **1** in toluene-d^8 at 90 ºC S25

Figure S18. Plot of [**6**] versus reaction time for the hydroalkoxylation of **6** catalyzed by catalyst **1** at different concentrations of **1** S26

Figure S19. Arrhenius plot for the cyclization of **6** catalyzed by **1** over the temperature range 60-90 ºC in toluene-d^8 S26

Figure S20. Eyring plot for the cyclization of **6** catalyzed by **1** over the temperature range 60-90 ºC in toluene-d^8 S27

Table S3. Crystal data and structure refinement for compounds **3** and **4** S28

Table S4. Selected bond distances (Å) and angles (°) for compounds **3** and **4** S29

3. Computational Details S30

4. References S44

1. EXPERIMENTAL SECTION

All handling of air- and moisture-sensitive compounds was conducted under dry nitrogen using either a Braun Labmaster glovebox or standard Schlenk line techniques. NMR spectra were acquired on a Bruker Ascend TM-500 spectrometer and referenced to the residual deuterated solvent. Elemental analyses were performed using a Perkin Elmer 2400 CHN analyzer. Solvents were pre-dried over sodium wire (toluene, THF, *n*-hexane) and distilled under nitrogen from sodium (toluene, THF) or sodium-potassium alloy (*n*-hexane). Deuterated solvents were stored over activated 4 Å molecular sieves and degassed by several freeze thaw cycles. 2-ethynylbenzyl alcohol (**6**), 4-pentynol (**8**), 5-hexynol (**9**), 5-(trimethylsilyl)-4-pentynol (**10**), 5-pentenoic acid (**11**) and 5-hexenoic acid (**12**) were purchased from Sigma-Aldrich. Substrates (2-((Trimethylsilyl)ethynyl)phenyl)methanol (**13**), (2-(Phenylethynyl)phenyl)methanol (**14**) and 6-bromo-5-hexynoic acid (**15**) were synthesized as previously described in literature.[1,2] All other reagents were procured from standard commercial suppliers and utilized without further purification.

Synthesis of [Zn(Et)(κ^3-bpzpam)] (1): In 100 mL Schlenk tube, bpzpamH (**L$_1$**) (0.50 g, 1.55 mmol) was dissolved in dry toluene (25 mL) and cooled to −50 °C. Then, a solution of $ZnEt_2$ (1 M in *n*-hexane, 1.55 mL, 1.55 mmol) was added, and the mixture was allowed to warm up and stirred for 1 h. After that time, the solvent was removed under reduced pressure and the residue washed with *n*-hexane to afford complex **1** as a white solid. Yield: 0.61 g (95 %). Anal. Calcd for $C_{20}H_{25}N_5OZn$: C, 57.6; H, 6.1; N, 16.8. Found: C, 57.8; H, 6.3; N, 16.4. ^{1}H NMR (500 MHz, C_6D_6, 297 K): δ 8.18 (d, J_{HH} = 7.0 Hz, 2H, ^oH-Ar), 7.34 (m, J_{HH} = 7.0 Hz, 2H, ^mH-Ar), 6.98 (t, J_{HH} = 7.0 Hz, 1H, ^pH-Ar), 6.74 (s, 1H, CH), 5.22 (s, 2H, H^4), 1.99 (s, 6H, Me^3), 1.82 (t, J_{HH} = 8.1 Hz, 3H, $ZnCH_2CH_3$), 1.79 (s, 6H, Me^5), 1.10 (m, J_{HH} = 8.1 Hz, 2H, $ZnCH_2CH_3$). ^{13}C{^{1}H} NMR (125 MHz, C_6D_6, 297 K): 164.6 (N*CO*), 149.8; 140.6 (C^3, C^5), 147.9 (C^i-Ar), 128.1 (C^m-Ar), 124.4 (C^p-NAr), 122.4 (C^o-NAr), 106.1 (C^4), 71.1 (CH), 13.5 ($ZnCH_2CH_3$), 12.3 (Me^3), 10.0 (Me^5), 1.4 ($ZnCH_2CH_3$).

Synthesis of [Zn(Et)(κ^3-bpzfam)] (2): The synthesis of **2** was performed following the same procedure as for compound **1**, using bpzfamH (**L$_2$**) (0.50 g, 1.22 mmol) and $ZnEt_2$ (1 M in *n*-hexane, 1.22 mL, 1.22 mmol). Compound **2** was isolated as a white solid. Yield: 0.55 g (90 %). Anal. Calcd for $C_{27}H_{29}N_5OZn$: C, 64.2; H, 5.8; N, 13.9. Found: C, 64.3; H, 5.9; N, 13.7 ^{1}H NMR (500 MHz, C_6D_6,

297 K): δ 8.32–7.00 (m, 7H, *Ar*-Flu), 6.67 (s, 1H, CH), 5.21 (s, 2H, H^4), 3.56 (s, 2H, *CH$_2$*-Flu), 1.96 (s, 6H, Me^3), 1.86 (t, J_{HH} = 8.1 Hz, 3H, $ZnCH_2$*CH$_3$*), 1.77 (s, 6H, Me^5), 1.04 (m, J_{HH} = 8.1 Hz, 2H, Zn*CH$_2$*CH$_3$). $^{13}C\{^1H\}$ NMR (125 MHz, C_6D_6, 297 K): 164.5 (N*CO*), 149.5; 140.9 (C^3, C^5), 129.9-119.9 (*Ar*-Flu), 106.2 (C^4), 71.2 (CH), 36.8 (*CH$_2$*-Flu), 13.0 ($ZnCH_2$*CH$_3$*), 12.5 (Me^3), 10.0 (Me^5), −2.0 (Zn*CH$_2$*CH$_3$).

Synthesis of [Zn(Et)(κ^3-(*S*)-bpzmpam)] (3): The synthesis of **3** was conducted following the same procedure as for compound **1**, using (*S*)-bpzmpamH (**L$_3$**) (0.50 g, 1.42 mmol) and $ZnEt_2$ (1 M in *n*-hexane, 1.42 mL, 1.42 mmol). Compound **3** was isolated as a white solid. Yield: 0.59 g (94 %). Anal. Calcd for $C_{22}H_{29}N_5OZn$: C, 59.4; H, 6.6; N, 15.7. Found: C, 59.6; H, 6.8; N, 15.4 ^1H NMR (500 MHz, C_6D_6, 297 K): δ 7.49 (d, J_{HH} = 7.0 Hz, 2H, ^{o}*H*-Ar), 7.20 (m, J_{HH} = 7.0 Hz, 2H, ^{m}*H*-Ar), 7.02 (t, J_{HH} = 7.0 Hz, 1H, ^{p}*H*-Ar), 6.99 (brs, 1H, CH), 5.74 (m, 1H, *CH*MePh), 5.25; 5.22 (s, 2H, $H^{4,4'}$), 2.07; 2.06 (s, 6H, $Me^{3,3'}$), 1.75 (brs, 3H, CH*Me*Ph), 1.70 (t, J_{HH} = 8.1 Hz, 3H, $ZnCH_2$*CH$_3$*), 1.65; 1.62 (s, 6H, $Me^{5,5'}$), 0.65 (m, J_{HH} = 8.1 Hz, 2H, Zn*CH$_2$*CH$_3$). $^{13}C\{^1H\}$ NMR (125 MHz, C_6D_6, 297 K): 165.8 (N*CO*), 149.0; 148.8; 148.2; 140,8; 140.6 ($C^{3,3',5,5'}$ and *C^i*-Ar), 127.9 (*C^m*-Ar), 126.6 (C^p-Ar), 125.8 (C^o-Ar), 105.9; 105.8 ($C^{4,4'}$), 70.0 (*CH*), 52.0 (*C*HMePh), 24.0 (CH*Me*Ph), 13.5 ($ZnCH_2$*CH$_3$*), 12.3; 12.2 ($Me^{3,3'}$), 10.1; 10.0 ($Me^{5,5'}$), −1.8 (Zn*CH$_2$*CH$_3$).

Synthesis of [Zn(Et)(κ^3-bpzptam)] (4): The synthesis of **4** was conducted following the same procedure as for compound **1**, using bpzptamH (**L$_4$**) (0.50 g, 1.48 mmol) and $ZnEt_2$ (1 M in *n*-hexane, 1.48 mL, 1.48 mmol). Compound **4** was isolated as a white solid. Yield: 0.61 g (95 %). Anal. Calcd for $C_{20}H_{25}N_5SZn$: C, 55.5; H, 5.8; N, 16.2. Found: C, 55.7; H, 5.9; N, 16.0 ^1H NMR (500 MHz, C_6D_6, 297 K): <u>Major Isomer (κ^3-NNS):</u> δ 7.40 (d, J_{HH} = 7.0 Hz, 2H, ^{o}*H*-Ar), 7.22 (m, J_{HH} = 7.0 Hz, 2H, ^{m}*H*-Ar), 7.01 (s, 1H, CH), 6.95 (t, J_{HH} = 7.0 Hz, 1H, ^{p}*H*-Ar), 5.31 (s, 2H, H^4), 2.05 (s, 6H, Me^3), 1.86 (s, 6H, Me^5), 1.84 (t, J_{HH} = 8.1 Hz, 3H, $ZnCH_2$*CH$_3$*), 1.00 (m, J_{HH} = 8.1 Hz, 2H, Zn*CH$_2$*CH$_3$). $^{13}C\{^1H\}$ NMR (125 MHz, C_6D_6, 297 K): 171.7 (N*CS*), 149.9; 141.1 (C^3, C^5), 151.8 (*C^i*-Ar), 128.8 (*C^m*-Ar), 123.9 (C^p-NAr), 122.3 (C^o-NAr), 106.6 (C^4), 74.3 (CH), 13.9 ($ZnCH_2$*CH$_3$*), 12.9 (Me^3), 10.6 (Me^5), −1.2 (Zn*CH$_2$*CH$_3$). <u>Minor Isomer (κ^3-NNN):</u> δ 7.66 (d, J_{HH} = 8.5 Hz, 2H, ^{o}*H*-Ar), 7.54 (s, 1H, *CH*), 7.23 (m, J_{HH} = 8.5 Hz, 2H, ^{m}*H*-Ar), 6.95 (t, J_{HH} = 8.5 Hz, 1H, ^{p}*H*-Ar), 5.26 (s, 2H, H^4), 1.97 (s, 6H,

Me^3), 1.94 (s, 6H, Me^5), 1.65 (t, J_{HH} = 8.1 Hz, 3H, ZnCH$_2$*CH$_3$*), 0.85 (m, J_{HH} = 8.1 Hz, 2H, Zn*CH$_2$*CH$_3$). ^{13}C{^{1}H} NMR (125 MHz, C$_6$D$_6$, 297 K): 187.2 (*NC*S), 149.9; 141.8 (C^3, C^5), 150.1 (*C*-Ar), 128.6 (*C^m*-Ar), 125.0 (C^p-Ar), 124.6 (C^o-Ar), 105.4 (C^4), 77.5 (CH), 13.5 (ZnCH$_2$*CH$_3$*), 12.8 (Me^3), 10.7 (Me^5), −2.7 (Zn*CH$_2$*CH$_3$).

Synthesis of [Zn(Et)(κ^3-bpzatam)] (5): The synthesis of **5** was performed following the same procedure as for compound **1**, using bpzatamH (**L$_5$**) (0.50 g, 1.26 mmol) and ZnEt$_2$ (1 M in *n*-hexane, 1.26 mL, 1.26 mmol). Compound **5** was isolated as a white solid. Yield: 0.53 g (85 %). Anal. Calcd for C$_{24}$H$_{35}$N$_5$SZn: C, 58.7; H, 7.2; N, 14.3. Found: C, 58.9; H, 7.5; N, 14.0. Major Isomer (κ^3-NNS): ^{1}H NMR (500 MHz, C$_6$D$_6$, 297 K): δ 6.83 (s, 1H, CH), 5.31 (s, 2H, H^4), 2.51 (brs, 6H, CH$_2$a), 2.07 (m, 3H, CHb), 2.04 (s, 6H, Me^3), 1.90 (t, J_{HH} = 8.1 Hz, 3H, ZnCH$_2$*CH$_3$*), 1.88 (s, 6H, Me^5), 1.69 (m, 6H, CH$_2$c), 1.03 (m, J_{HH} = 8.1 Hz, 2H, Zn*CH$_2$*CH$_3$). ^{13}C{^{1}H} NMR (125 MHz, C$_6$D$_6$, 297 K): 164.1 (N*CS*), 149.0; 140.5 (C^3, C^5), 106.0 (C^4), 76.0 (CH), 57.0-30.1 (Ad), 13.7 (ZnCH$_2$*CH$_3$*), 12.6 (Me^3), 10.4 (Me^5), −1.6 (Zn*CH$_2$*CH$_3$). Minor Isomer (κ^3-NNN): ^{1}H NMR (500 MHz, C$_6$D$_6$, 297 K): δ 7.00 (s, 1H, CH), 5.46; 5.24 (s, 2H, H$^{4,4'}$), 2.78; 2.60; 2.51 (brs, 6H, CH$_2$a), 2.07 (s, 3H, CHb), 2.04 (s, 6H, Me^3), 1.94 (s, 6H, Me^5), 1.83 (m, 3H, ZnCH$_2$*CH$_3$*), 1.72-1.50 (m, 6H, CH$_2$c), 0.91 (m, 2H, Zn*CH$_2$*CH$_3$). ^{13}C{^{1}H} NMR (125 MHz, C$_6$D$_6$, 297 K): 184.9 (N*CS*), 149.0; 141.1 (C^3, C^5), 106.2 (C$^{4,4'}$), 79.7 (CH), 58.4-29.0 (Ad), 13.3 (ZnCH$_2$*CH$_3$*), 12.4 (Me^3), 10.8 (Me^5), 1.3 (Zn*CH$_2$*CH$_3$).

X-ray Crystallographic Structure Determination

Tables S1 and S2 contain data regarding data collection, refinement parameters and selected bond lengths and angles for complexes **3** and **4** (major isomer).A single crystal was coated with high-vacuum grease, mounted on a glass fiber, and transferred to a D8 Venture and a Bruker APEX II CCD-based diffractometer equipped with a graphite-monochromated MoK radiation source (λ=0.71073 Å). The datasets were integrated using SAINT[3] and corrected for Lorentzian and polarization effects. A semi-empirical absorption correction was applied to the diffraction data using SADABS.[4] The software package OLEX[5] was employed for structure solution and refinement by full-matrix least-squares methods based on F2. A successful solution by direct methods provided most non-hydrogen atoms from the E map. The remaining non-hydrogen atoms were located through an

alternating series of least-squares cycles and difference Fourier maps. Anisotropic displacement coefficients were refined for non-hydrogen atoms, while hydrogen atoms were placed by using a riding model and included in the refinement at calculated positions.

The structures of **3** and **4** (major isomer) have CCDC reference numbers 2332768 and 2332769, respectively. Copies of the data are available free of charge upon request to CCDC, 12 Union Road, Cambridge CB2 1EZ, U.K. (fax, (+44)1223 336-033; e-mail, deposit@ ccdc.cam.ac.uk).

General procedure for catalytic intramolecular hydroalkoxylation/carboxylation

In the glovebox, catalyst **1–5**, alkynyl substrates **6**, **8–15** and mesitylene as internal standard were dissolved in toluene-d_8 and placed in a J. Young style NMR tube with a resealable Teflon valve. The tube was closed and placed in a oil bath preheated to the desired temperature. The reaction was monitored by ^{1}H NMR spectroscopy to determine the conversion.

Typical NMR-scale catalytic reaction: In the glovebox, the corresponding alkynyl substrate (0.5 mmol), ethyl zinc catalyst **1** (0.021 g, 0.025 mmol) and mesitylene (0.5 mmol) were dissolved in toluene-d_8 (0.6 mL). The resultant solution was quickly transferred to a J. Young style NMR tube which was immediately placed into an oil bath that was preheated to the desired temperature. Single scan spectra were acquired automatically at different time intervals. The conversion of product at any given time was determined by integration of mesitylene and product resonances.

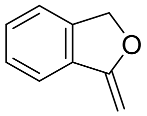

1-Methylene-1, 3-dihydroisobenzofuran (7). ^{1}H NMR (500 MHz, toluene-d_8): δ 7.20 (m, 1H), 6.99 (m, 2H), 6.74 (m, 1H), 4.83 (s, 2H), 4.63 (d, 1H, J = 2.1 Hz), 4.50 (d, 1H, J = 2.1 Hz).

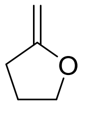

2-Methylene-tetrahydrofuran (8'). ^{1}H NMR (500 MHz, toluene-d_8): δ 4.35 (d, 1H, J = 1.6 Hz), 3.82 (d, 1H, J = 1.5 Hz), 3.72 (t, 2H, J = 6.7 Hz), 2.20 (m, 2H), 1.50 (q, 2H, J = 7.1 Hz).

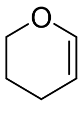

3,4-dihydro-2*H*-pyran (8''). ^{1}H NMR (500 MHz, toluene-d_8): δ 6.63 (s, 1H), 4.43 (s, 1H), 4.10 (t, 2H, J = 9.3 Hz), 2.36 (m, 2H), 1.68 (m, 2H).

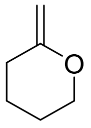

2-Methylene-tetrahydropyran (9'). ^{1}H NMR (500 MHz, toluene-d_8): δ 4.50 (s, 1H), 4.03 (s, 1H), 3.61 (t, 2H, J = 5.3 Hz), 1.99 (t, 2H, J = 6.4 Hz), 1.35 (q, 2H, J = 6.0 Hz), 1.29 (q, 2H, J = 5.6 Hz).

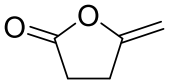

5-Methylenedyihydrofuran-2(*3H*)-one (10'). ^{1}H NMR (500 MHz, toluene-d_8): δ 4.54 (s, 1H), 3.90 (s, 1H), 2.00 (t, 2H, J = 9.3 Hz), 1.80 (t, 2H, J = 8.8 Hz).

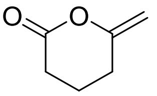

6-Methylenetetrahydro-*2H*-pyran-2-one (11'): ^{1}H NMR (500 MHz, toluene-d_8): δ 4.49 (s, 1H), 3.91 (s, 1H), 1.98 (t, 2H, J = 6.8 Hz), 1.77 (t, 2H, J = 6.4 Hz), 1.07 (q, 2H, J = 6.7 Hz).

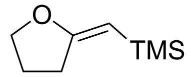

(*E*)-((dihydrofuran-2(*3H*)-ylidene)methyl)trimethylsilane (12'): ^{1}H NMR (500 MHz, toluene-d_8): δ 4.69 (s, 1H), 3.63 (t, 2H, J = 6.6 Hz), 2.17 (m, 2H), 1.43 (q, 2H, J = 7.1 Hz), 0.15 (s, 9H).

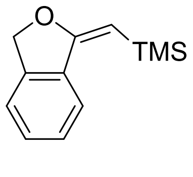

(*E*)-(isobenzofuran-1(3H)-ylidenemethyl)trimethylsilane (13'): ^{1}H NMR (500 MHz, toluene-d_8): δ 7.76 (d, 2H, J = 7.8 Hz), 7.36 (m, 2H), 6.73 (d, 2H, J = 7.6 Hz), 5.15 (s, 1H), 4.79 (s, 2H), 0.32 (s, 9H).

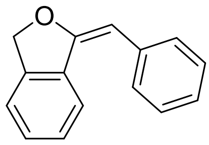

(*E*)-1-benzylidene-1,3-dihydroisobenzofuran (14'): ^{1}H NMR (500 MHz, toluene-d_8): δ 7.45 (d, 1H, *J* = 7.9 Hz), 7.36 (d, 2H, *J* = 7.6 Hz), 7.17 (t, 2H, *J* = 7.6 Hz), 7.08 (m, 1H), 6.91 (t, 1H, *J* = 7.4 Hz), 6.76 (t, 1H, *J* = 7.7 Hz), 6.71 (d, 1H, *J* = 7.7 Hz), 6.44 (s, 1H), 4.87 (s, 2H).

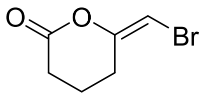

(*E*)-6-(bromomethylene)tetrahydro-2H-pyran-2-one (15'): ^{1}H NMR (500 MHz, toluene-d_8): δ 4.72 (s, 1H), 1.85 (m, 2H), 1.48 (m, 2H), 0.85 (m, 2H, *J* = 6.8 Hz)

General procedure for kinetic measurements

In a glovebox, a J. Young style NMR tube was charged with of the catalyst **1** and the substrate **6** in toluene-d_8. The conversion was monitored by ^{1}H NMR spectroscopy by following the disappearance of the alkyne signals of the substrate.

Representative example: In the kinetic studies to determine the rate dependence on catalyst in the cyclization of 2-ethynylbenzyl alcohol (**6**), 0.044 g (0.33 mmol) of substrate **6** and a 96 µL of solution containing a known concentration of catalyst **1** (0.2205 M in toluene-d_8), were placed in a glass vial. Then, toluene-d_8 was added in order to complete the total volume of 0.6 mL and the dissolved mixture was transferred to a J. Young style NMR tube. The tube was closed and placed into a Bruker Ascend TM-500 NMR spectrometer that was preheated at 90 ºC. The reaction was monitored at regular intervals (2 min) by ^{1}H NMR spectroscopy.

2. FIGURES AND TABLES REFERRED TO IN MAIN TEXT

Table S1. NCO ^{13}C-NMR chemical shifts for complexes **1-3** and neutral heteroscorpionate ligand precursors in C_6D_6.

Complex	*δ* (NCO) (ppm)	Free Ligand	*δ* (NCE) (ppm)
[Zn(Et)(κ^3-bpzpam)] (**1**)	164.6	bpzpamH	162.2
[Zn(Et)(κ^3-bpzfam)] (**2**)	164.5	bpzfamH	162.4
[Zn(Et)(κ^3-(*S*)-bpzmpam)] (**3**)	165.8	(*S*)-bpzmpamH	163.6

Table S2. NCS ^{13}C-NMR chemical shifts for complexes **4-5** and neutral heteroscorpionate ligand precursors in C_6D_6.

Complex	*δ* (NCS) (ppm)	Free Ligand	*δ* (NCE) (ppm)

[Zn(Et)(κ^3-bpzptam)] (**4**)	171.3 (76%)<br>187.2 (24%)	bpzptamH	189.0
[Zn(Et)(κ^3-bpzatam)] (**5**)	164.1 (85%)<br>185.1 (15%)	bpzatamH	188.1

Figure S1. NMR spectra for [Zn(Et)(κ^3-bpzpam)] (**1**) in C_6D_6.

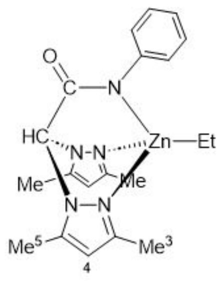
O
C
N
HC
N-N
Zn
Et
Me
Me
N-N
Me5
Me3
4

(1)

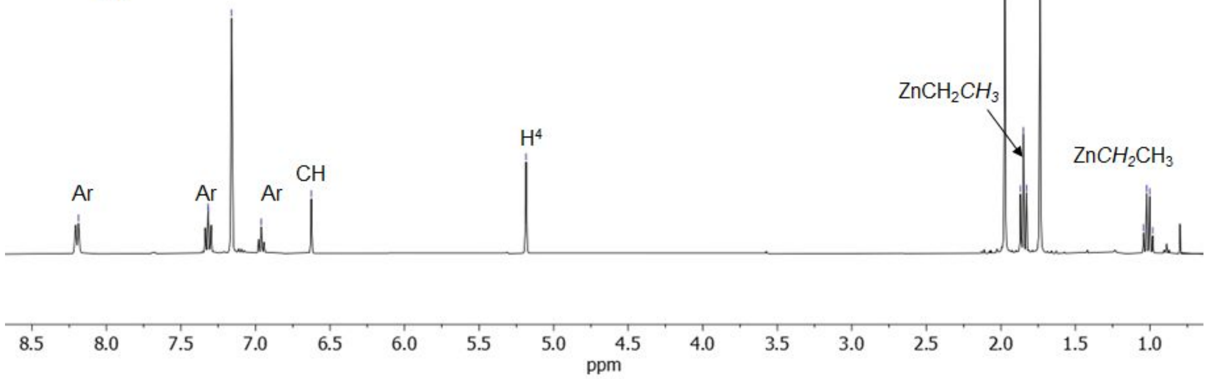
Me3
Me5
ZnCH2CH3
H4
ZnCH2CH3
Ar
Ar
Ar
CH
8.5
8.0
7.5
7.0
6.5
6.0
5.5
5.0
4.5
4.0
3.5
3.0
2.5
2.0
1.5
1.0
ppm

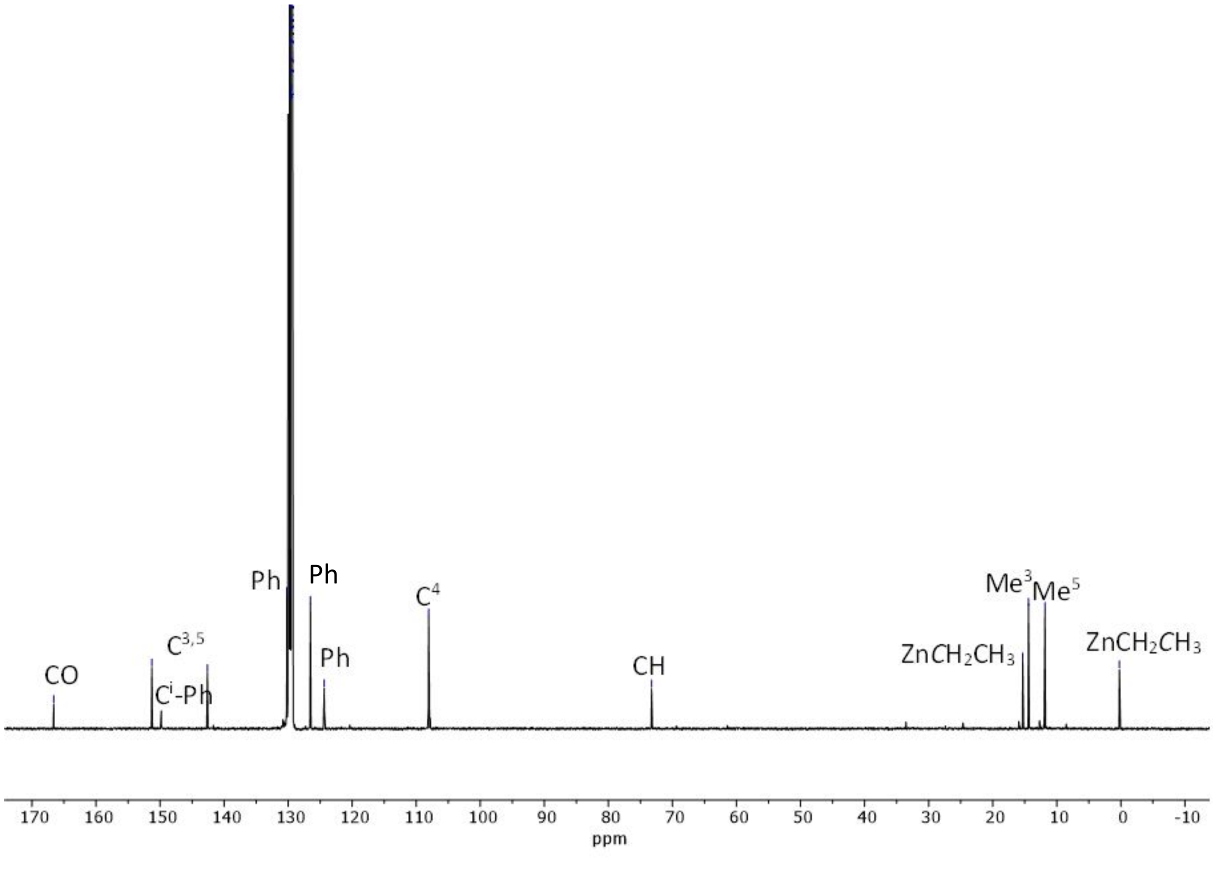
Ph
Ph
C4
Me3
Me5
C3,5
Ph
ZnCH2CH3
ZnCH2CH3
CO
Ci-Ph
CH
170
160
150
140
130
120
110
100
90
80
70
60
50
40
30
20
10
0
-10
ppm

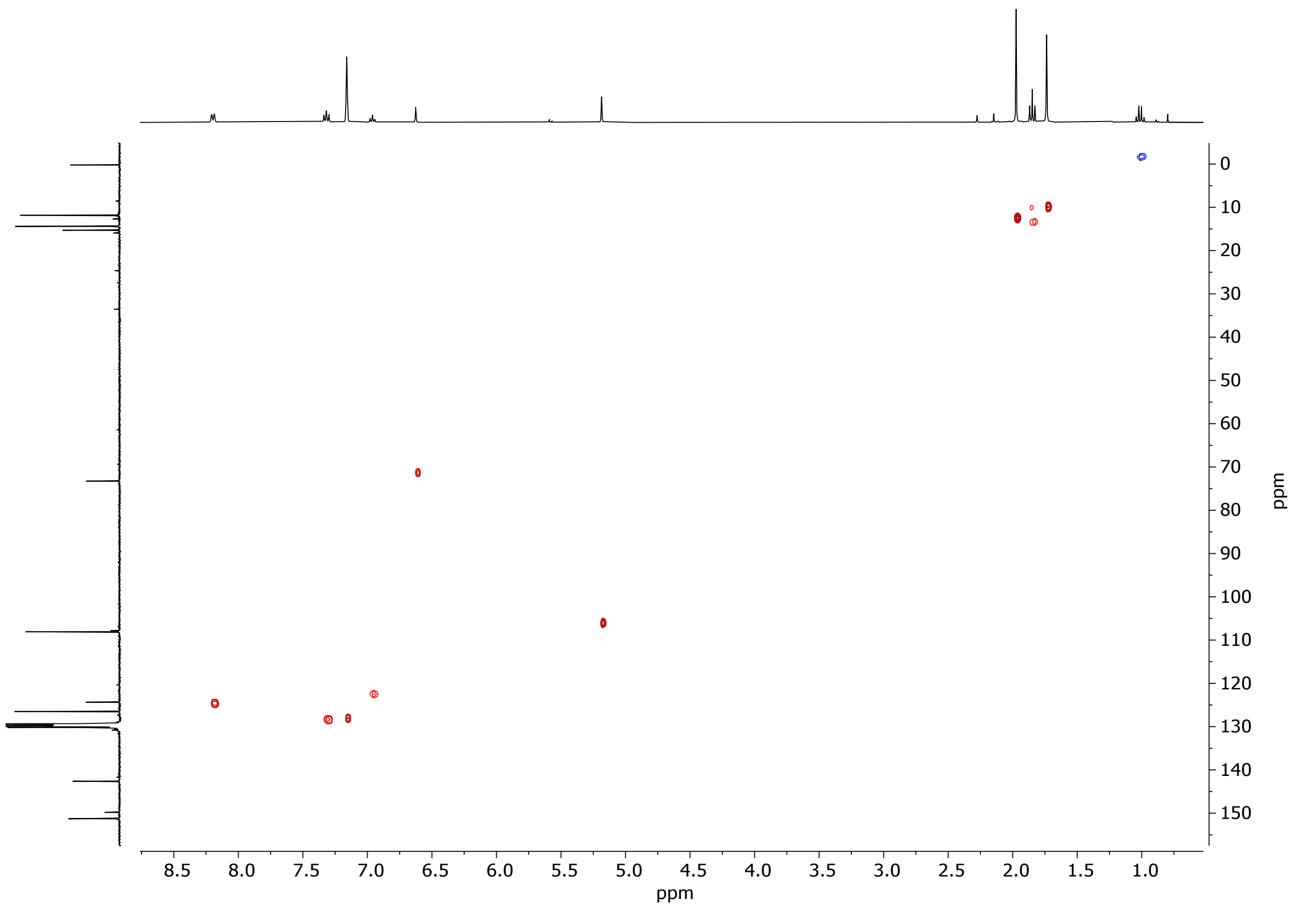

0
10
20
30
40
50
60
70
80
90
100
110
120
130
140
150
ppm
8.5 8.0 7.5 7.0 6.5 6.0 5.5 5.0 4.5 4.0 3.5 3.0 2.5 2.0 1.5 1.0
ppm

Figure S2. NMR spectra for [Zn(Et)(κ^3-bpzfam)] (**2**) in C_6D_6.

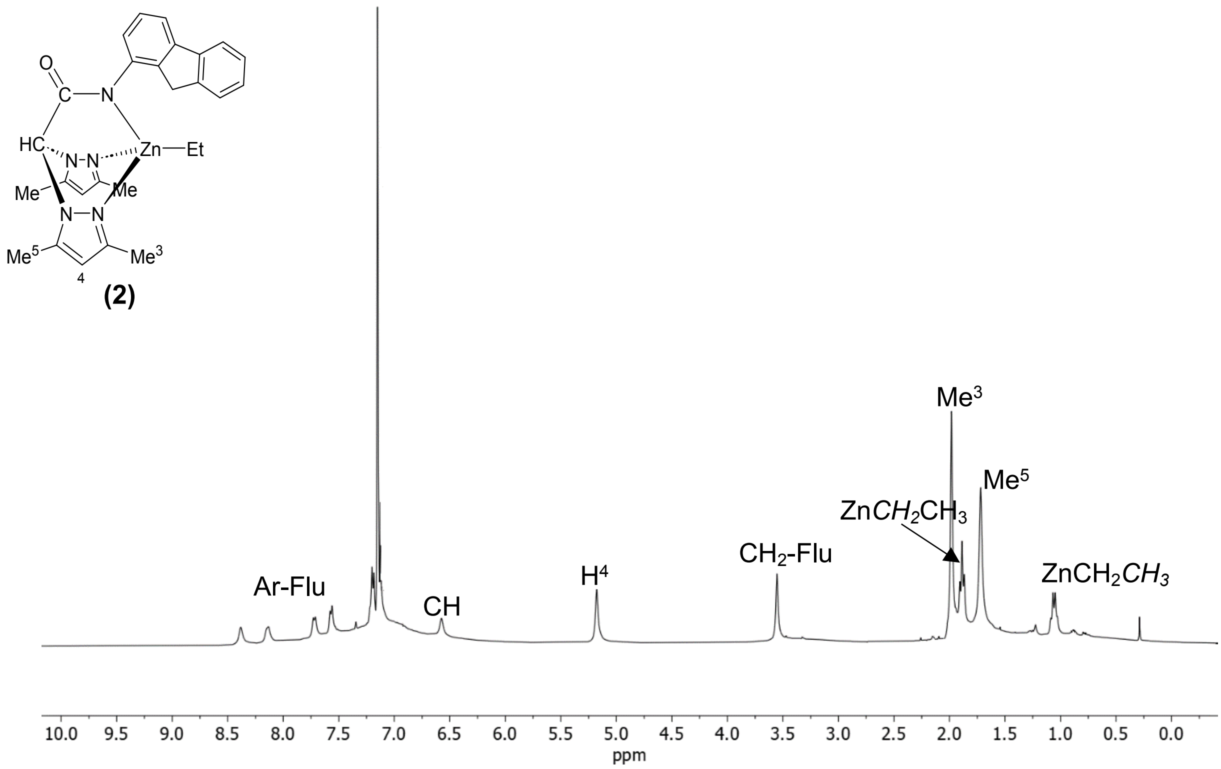

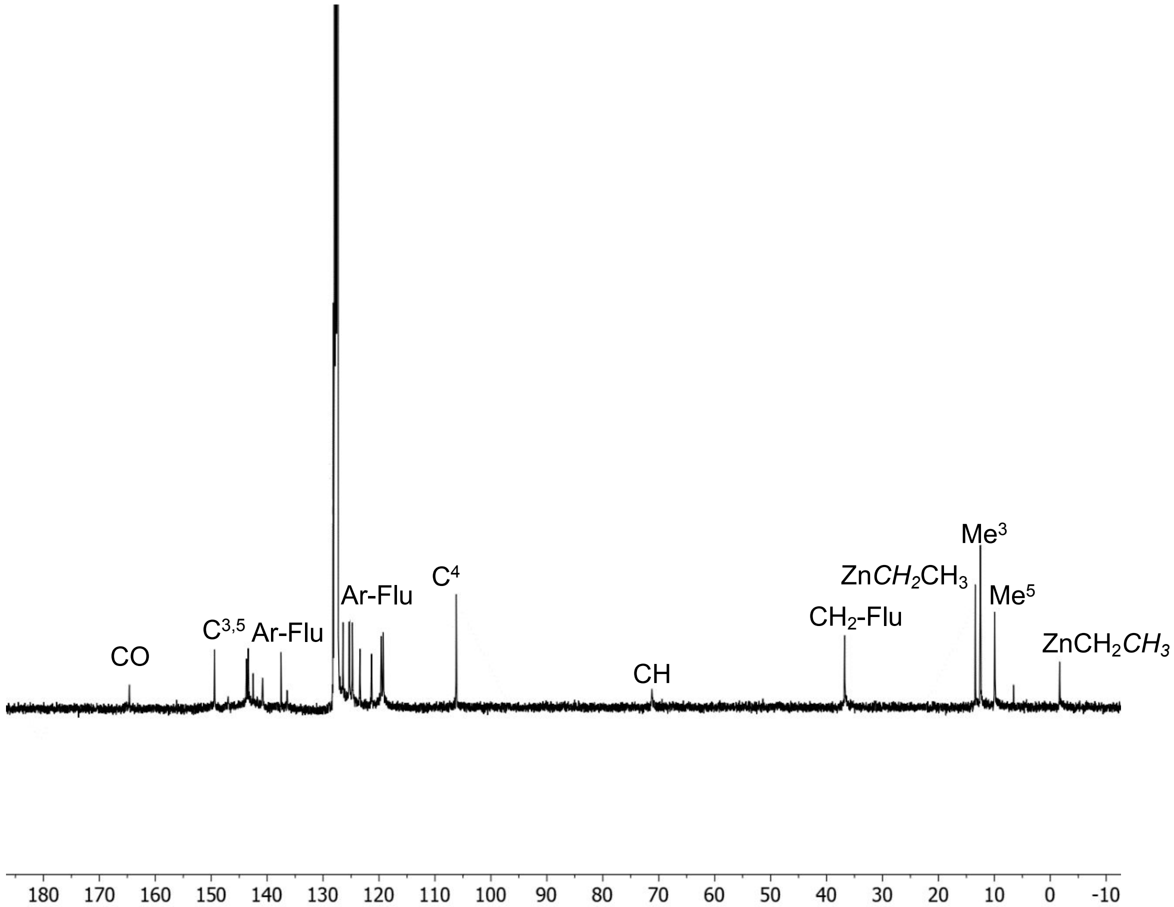

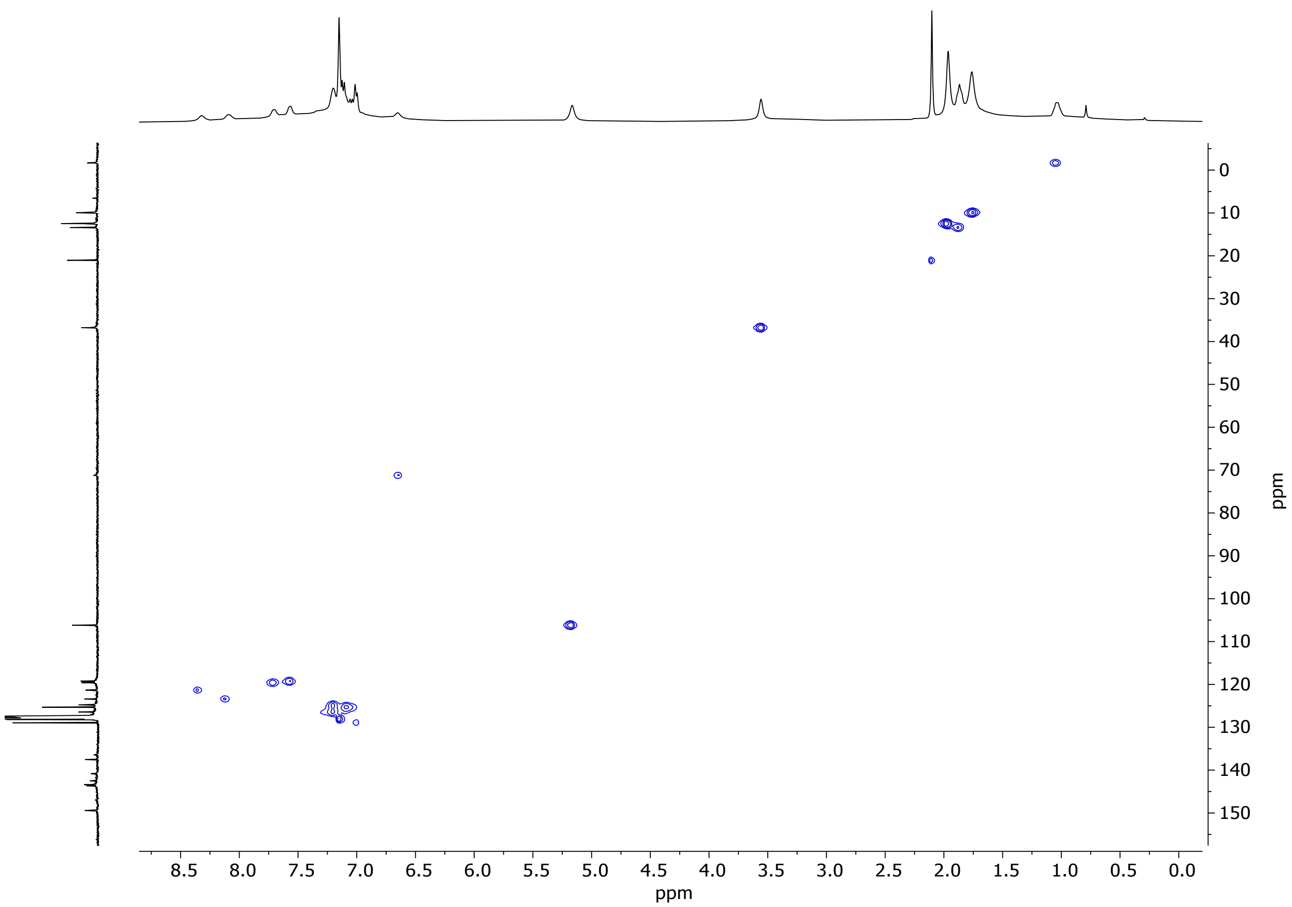
0
10
20
30
40
50
60
70
80
90
100
110
120
130
140
150
ppm
8.5 8.0 7.5 7.0 6.5 6.0 5.5 5.0 4.5 4.0 3.5 3.0 2.5 2.0 1.5 1.0 0.5 0.0
ppm

Figure S3. NMR spectra for [Zn(Et)(κ^3-(*S*)-bpzmpam)] (**3**) in C_6D_6.

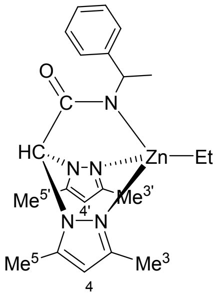

(3)

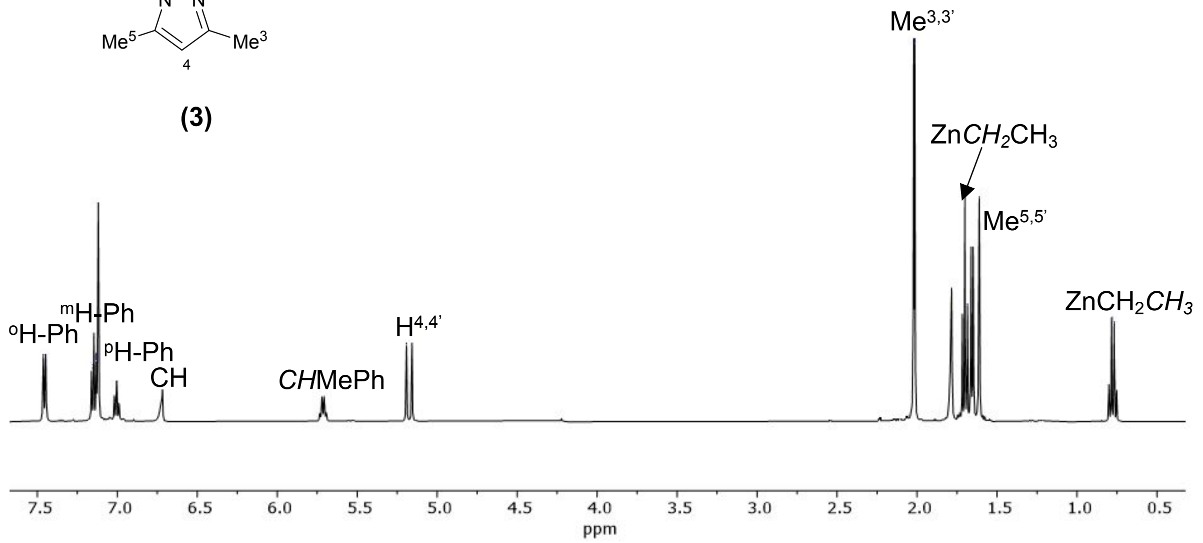

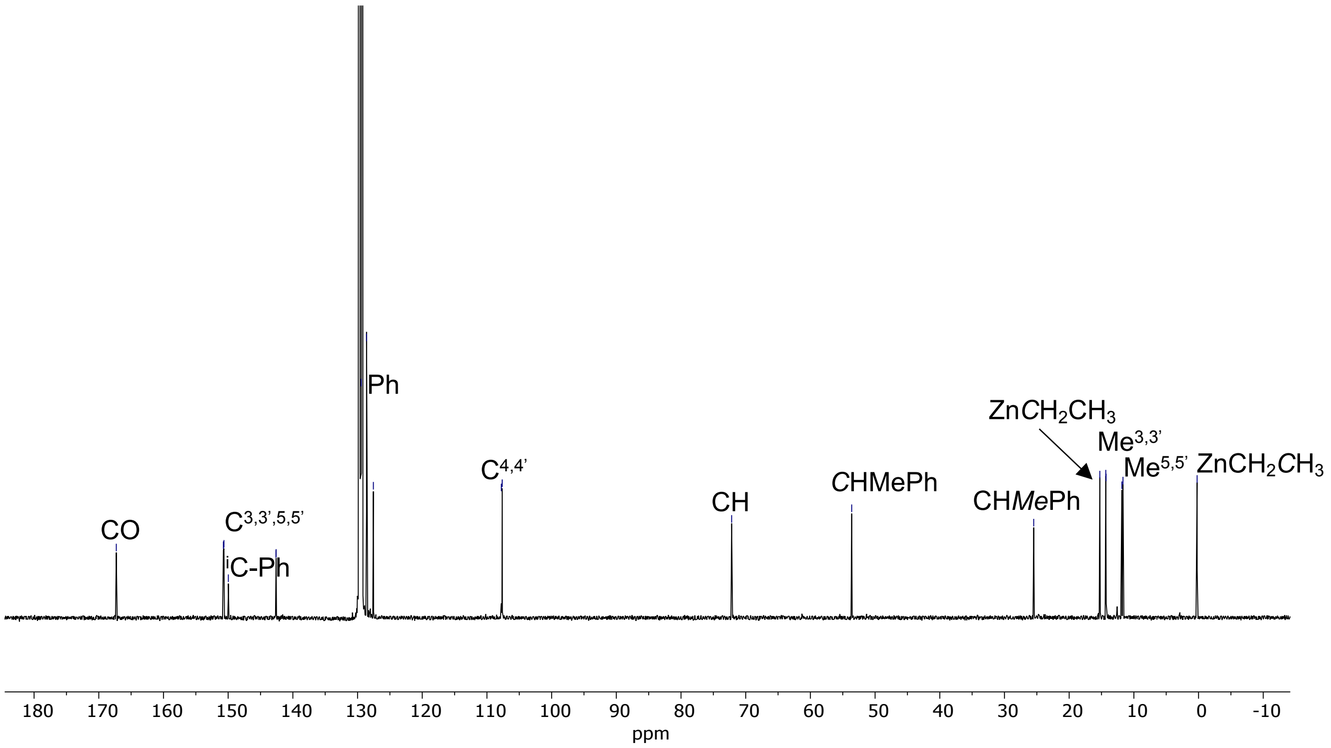

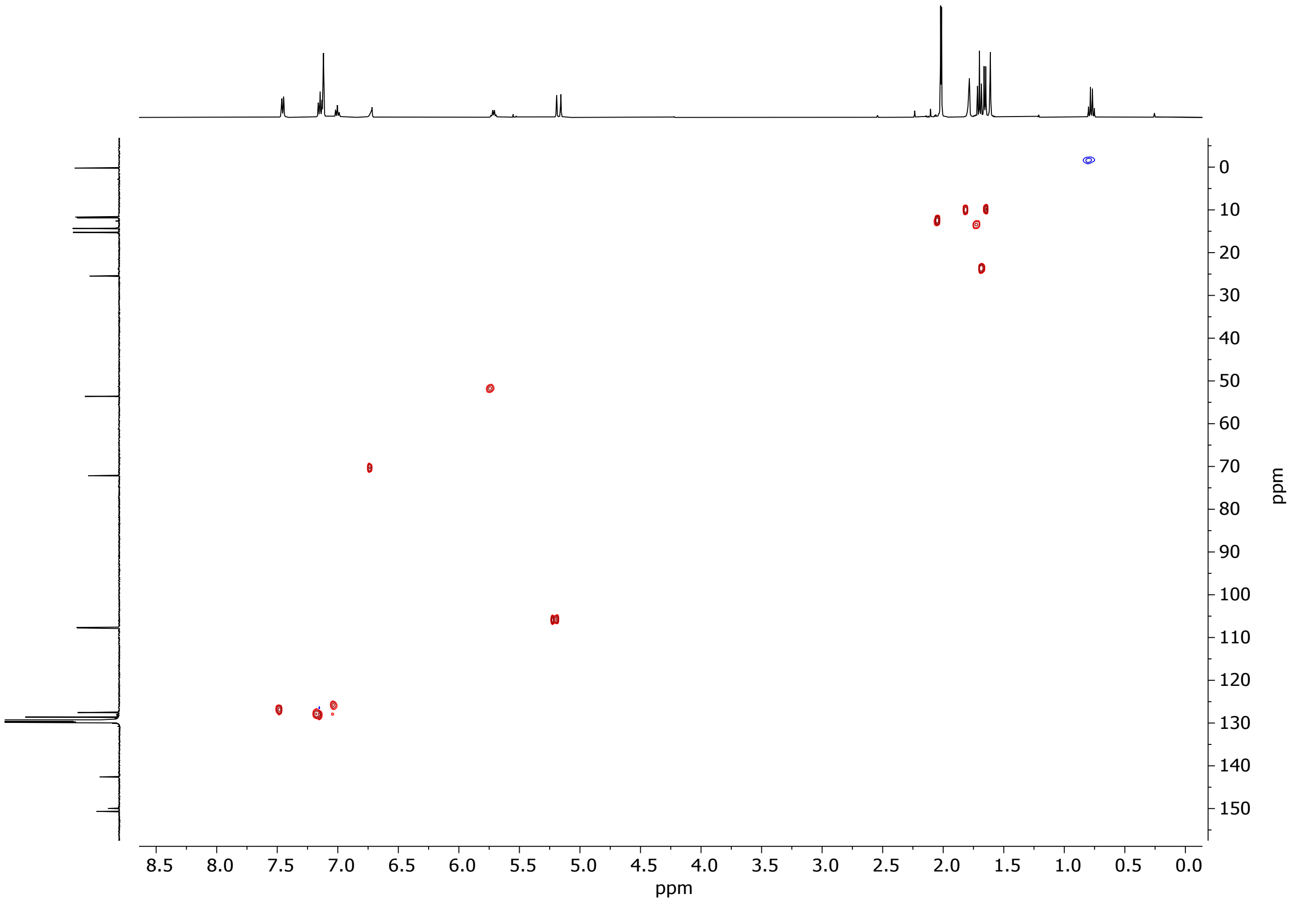
8.5
8.0
7.5
7.0
6.5
6.0
5.5
5.0
4.5
4.0
3.5
3.0
2.5
2.0
1.5
1.0
0.5
0.0
ppm
0
10
20
30
40
50
60
70
80
90
100
110
120
130
140
150
ppm

Figure S4. NMR spectra for [Zn(Et)(κ^3-bpzptam)] (**4**) in C_6D_6.

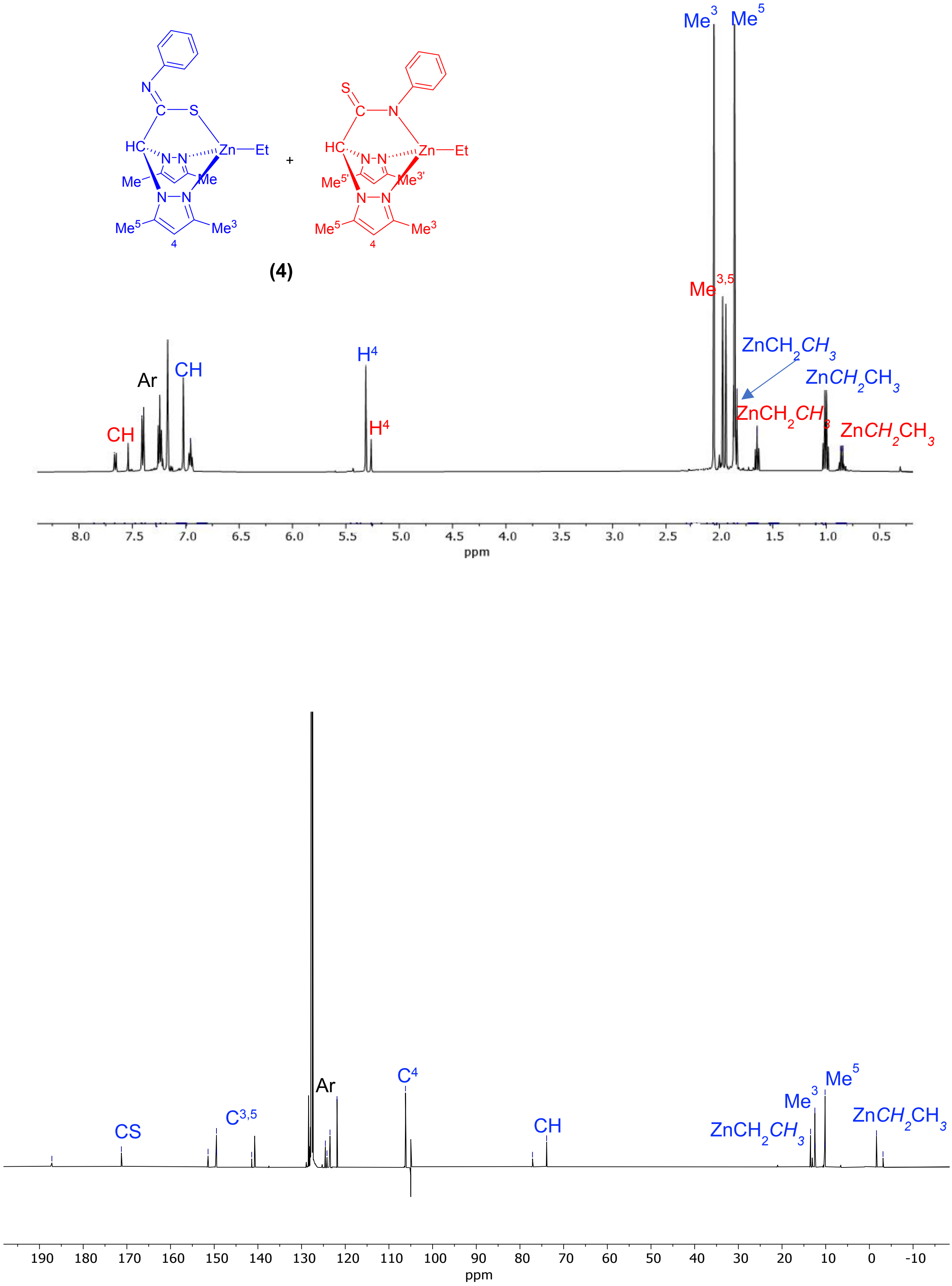

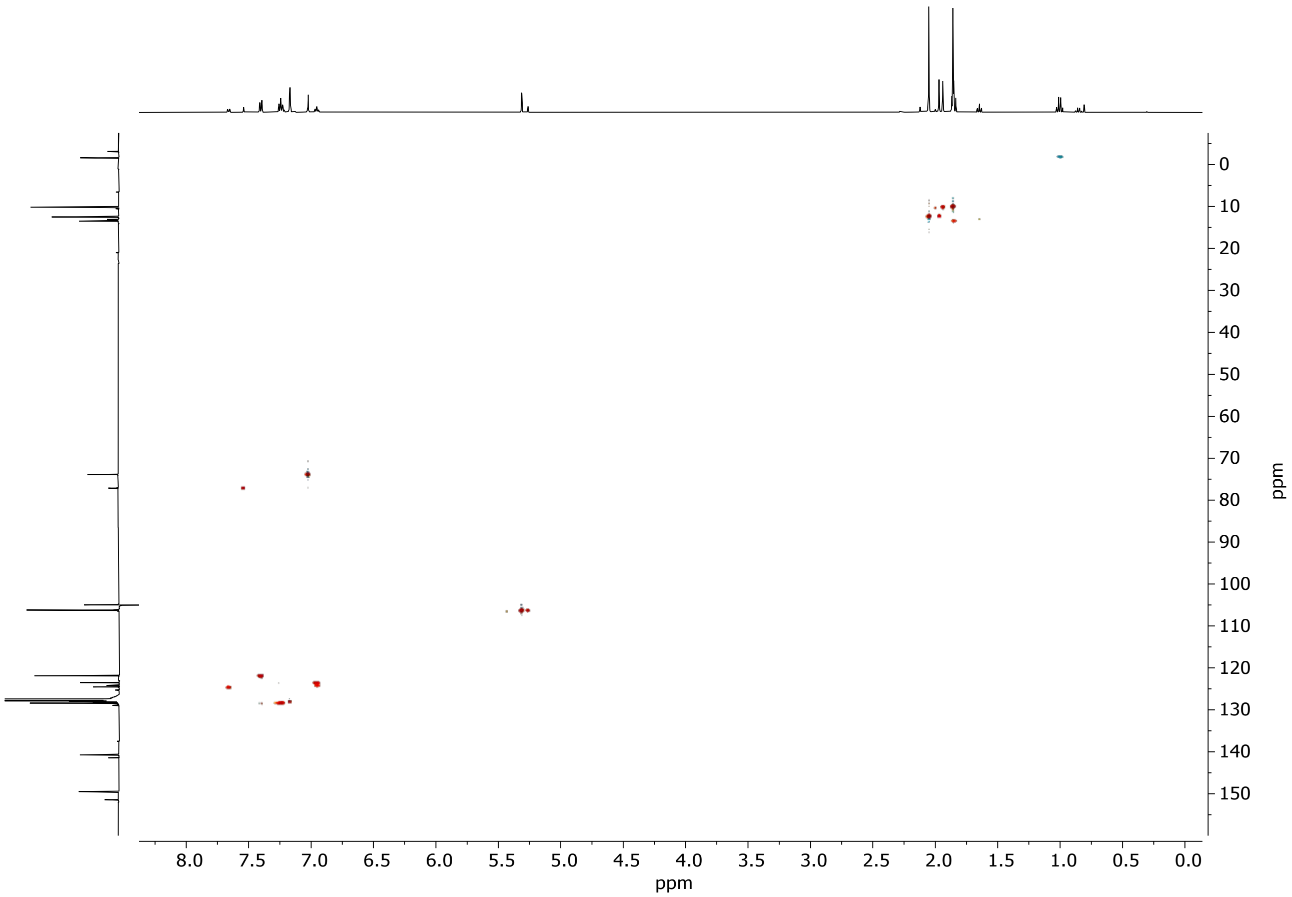

ppm
ppm

Figure S5. NMR spectra for [Zn(Et)(κ^3-bpzatam)] (**5**) in C_6D_6.

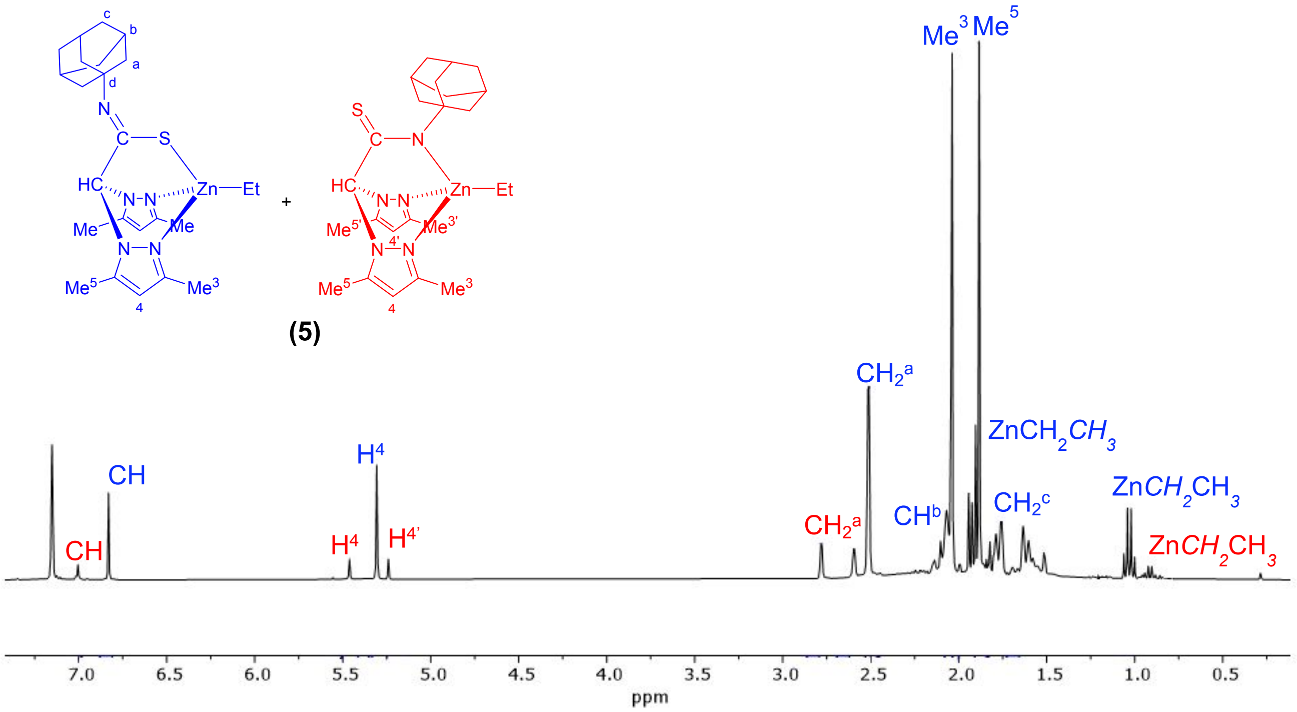

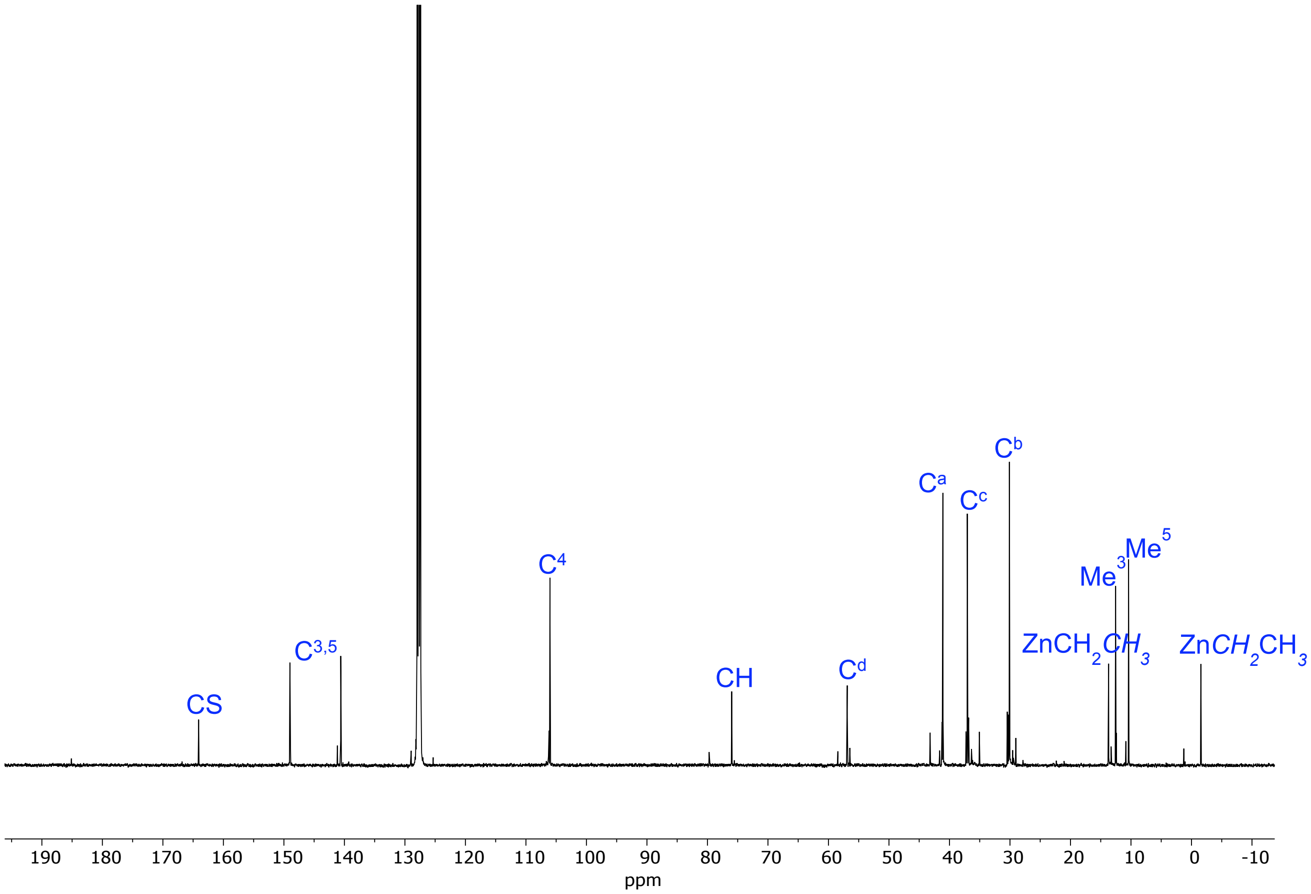

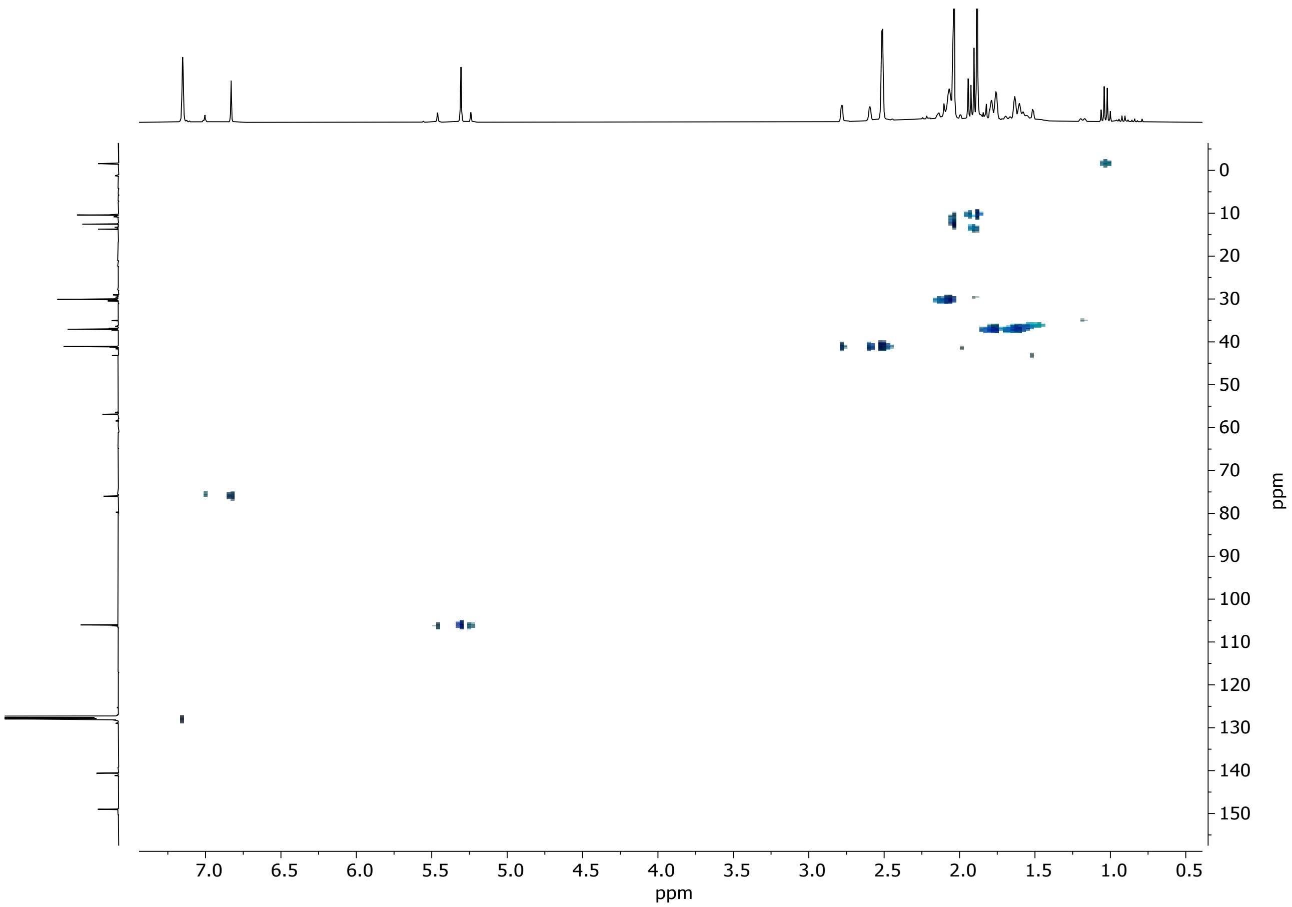
0
10
20
30
40
50
60
70
80
90
100
110
120
130
140
150
ppm
7.0
6.5
6.0
5.5
5.0
4.5
4.0
3.5
3.0
2.5
2.0
1.5
1.0
0.5
ppm

Figure S6. NMR crude (500 MHz, toluene-d_8, 25 ºC) for the reaction of substrate **6** to afford cyclized product **7** using catalyst **1** (Table 1, entry 1).

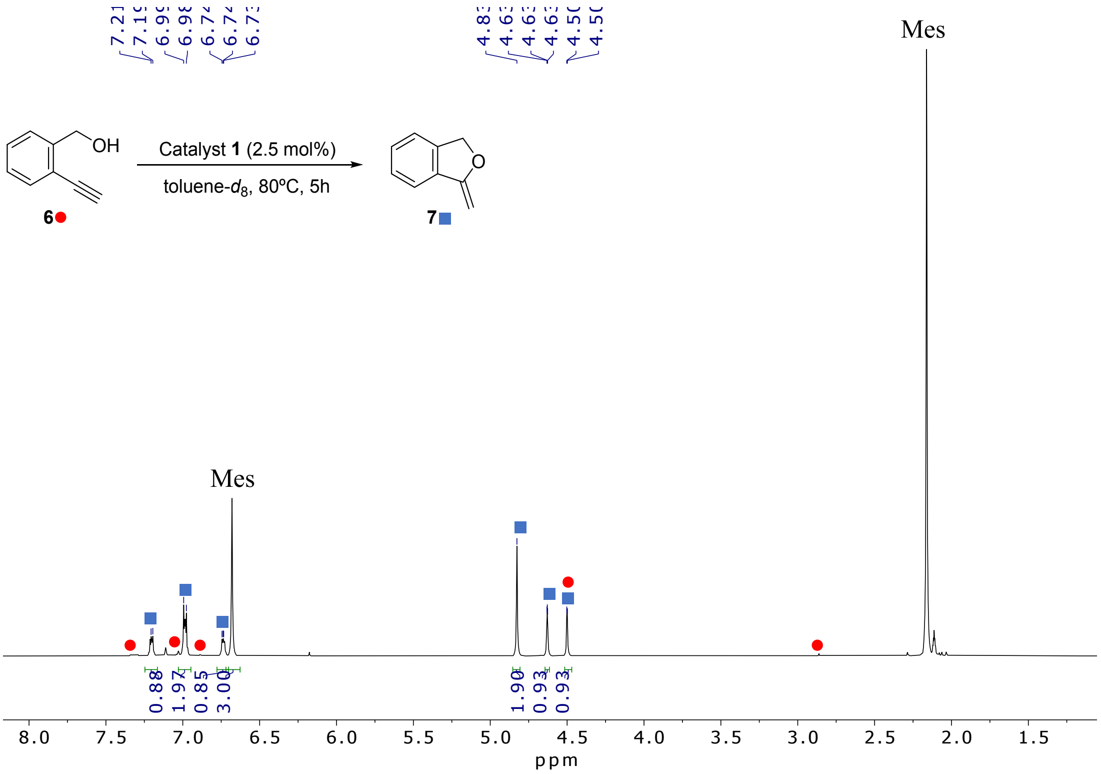

Figure S7. NMR crude (500 MHz, toluene-d_8, 25 ºC) for the reaction of substrate **8** to afford cyclized products **8’** and **8’’** using catalyst **1** (Table 2, entry 1).

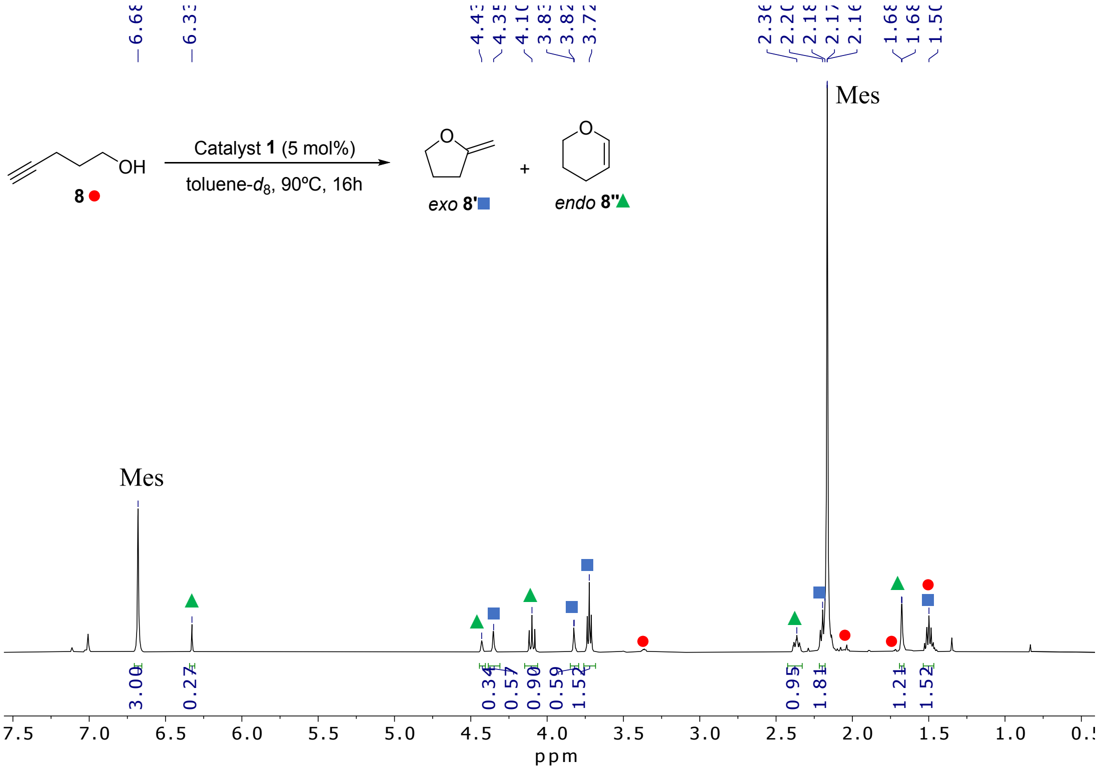

Figure S8. NMR crude (500 MHz, toluene-d_8, 25 ºC) for the reaction of substrate **9** to afford cyclized product **9'** using catalyst **1** (Table 2, entry 2).

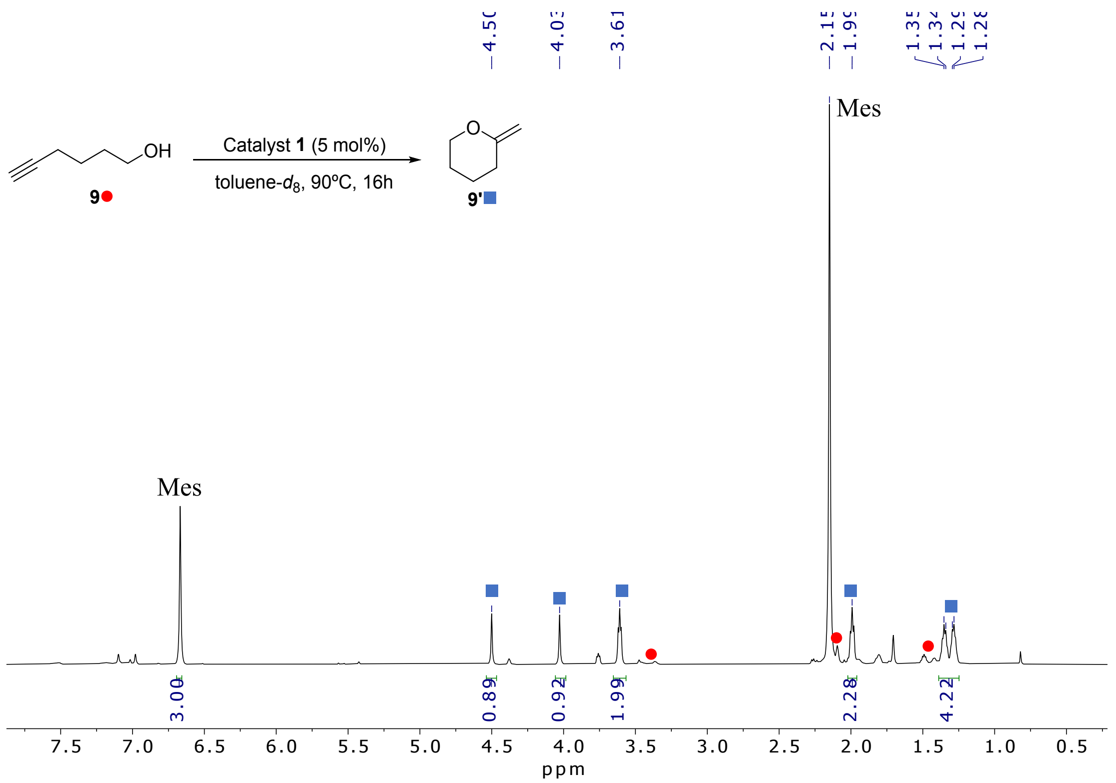

Figure S9. NMR crude (500 MHz, toluene-d_8, 25 ºC) for the reaction of substrate **10** to afford cyclized product **10'** using catalyst **1** (Table 2, entry 3).

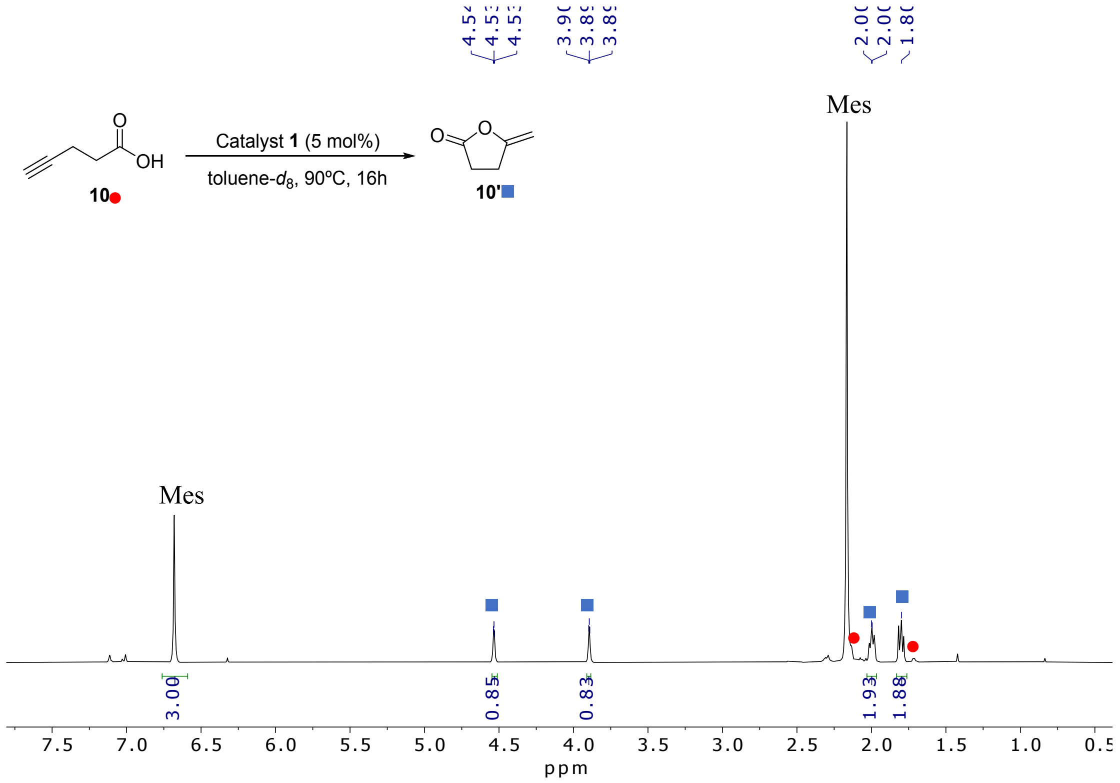

Figure S10. NMR crude (500 MHz, toluene-d_8, 25 ºC) for the reaction of substrate **11** to afford cyclized product **11'** using catalyst **1** (Table 2, entry 4).

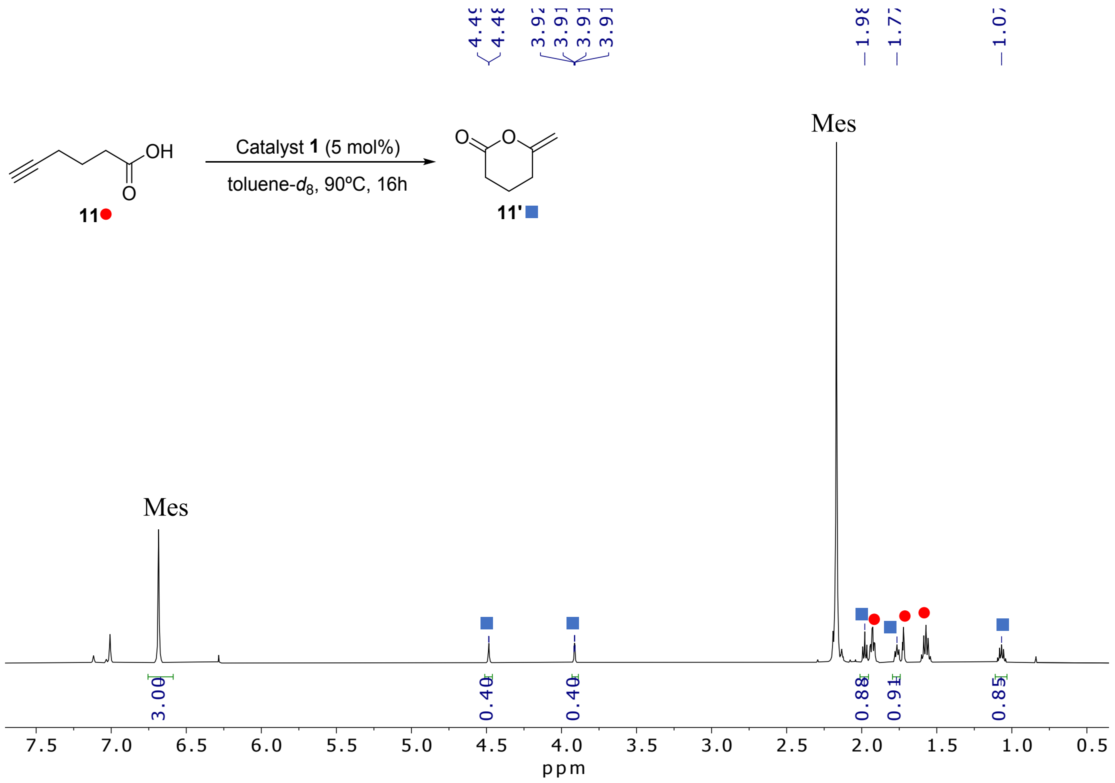

Figure S11. NMR crude (500 MHz, toluene-d_8, 25 ºC) for the reaction of substrate **12** to afford cyclized product **12'** using catalyst **1** (Table 2, entry 5).

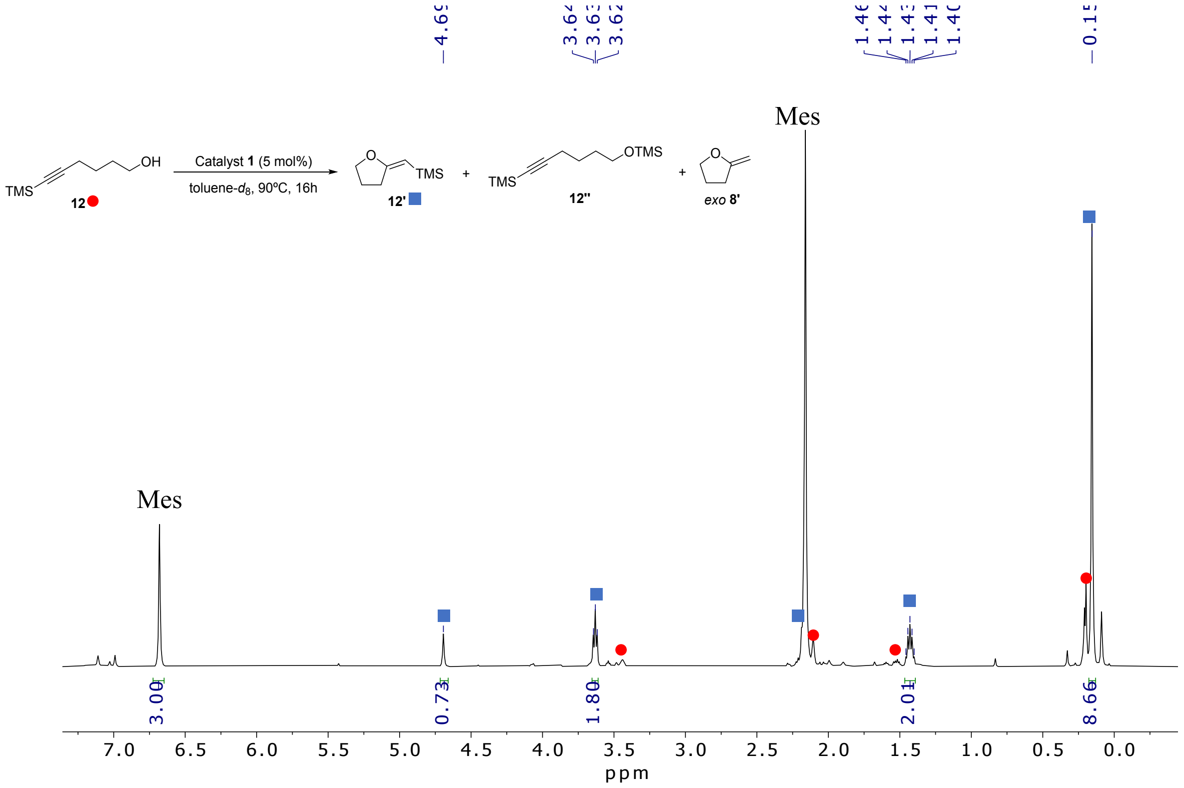

Figure S12. NMR crude (500 MHz, toluene-d_8, 25 ºC) for the reaction of substrate **13** to afford cyclized product **13’** using catalyst **1** (Table 2, entry 6).

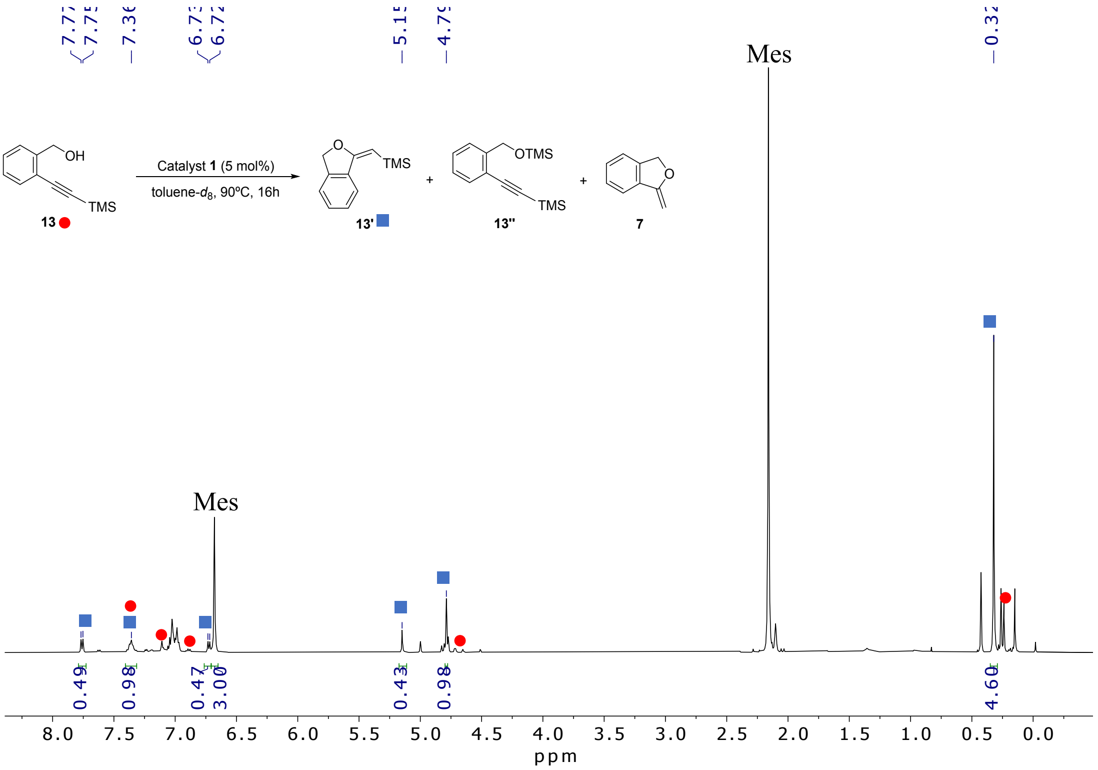

Figure S13. NMR crude (500 MHz, toluene-d_8, 25 ºC) for the reaction of substrate **14** to afford cyclized product **14’** using catalyst **1** (Table 2, entry 7).

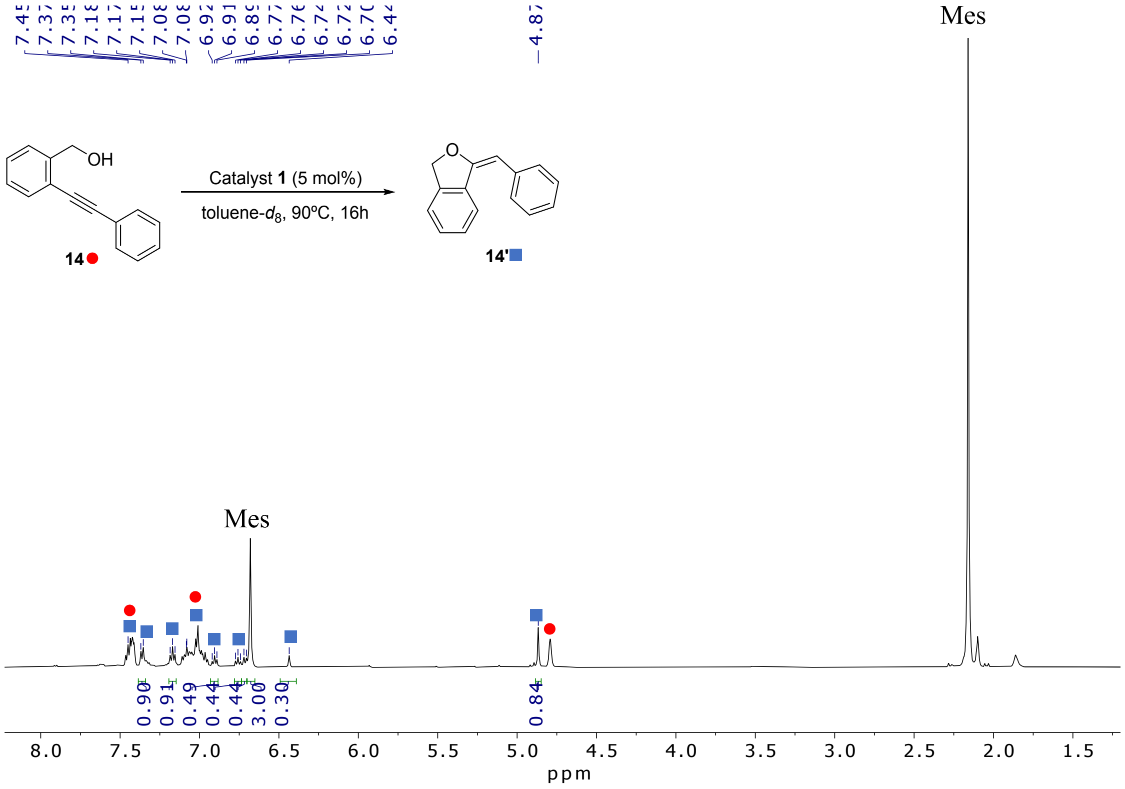

Figure S14. NMR crude (500 MHz, toluene-d_8, 25 ºC) for the reaction of substrate **15** to afford cyclized product **15'** using catalyst **1** (Table 2, entry 8).

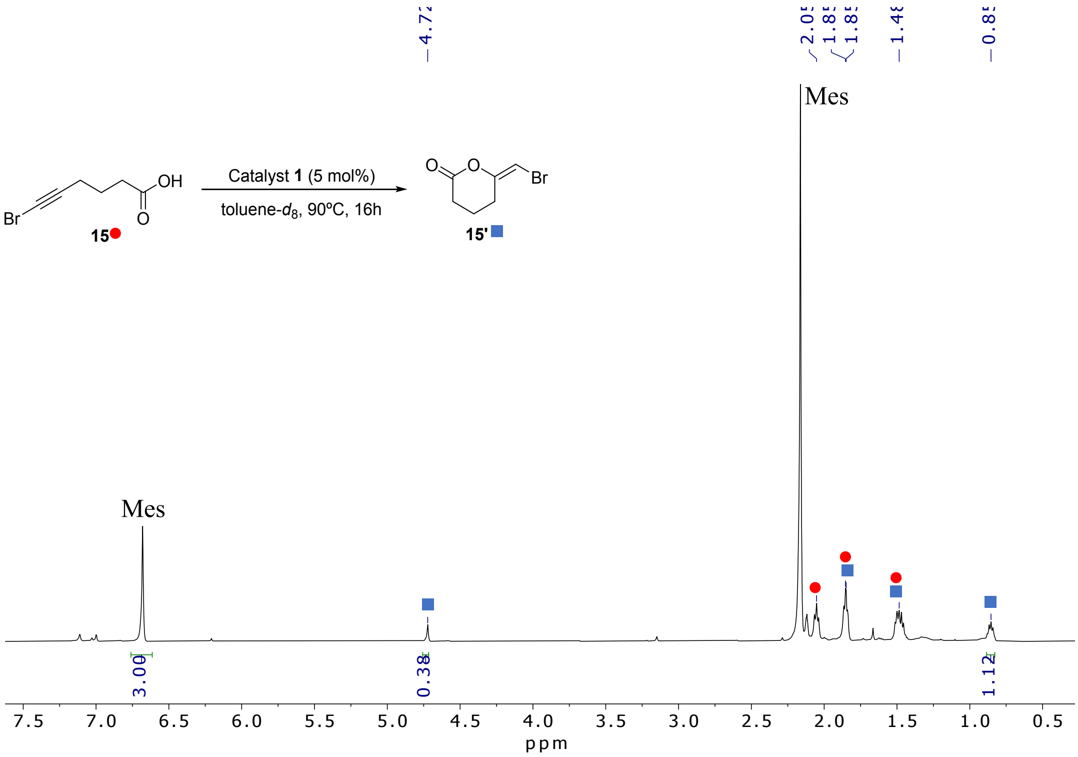

Figure S15. NOESY-1D experiment performed for cyclized product **13'**.

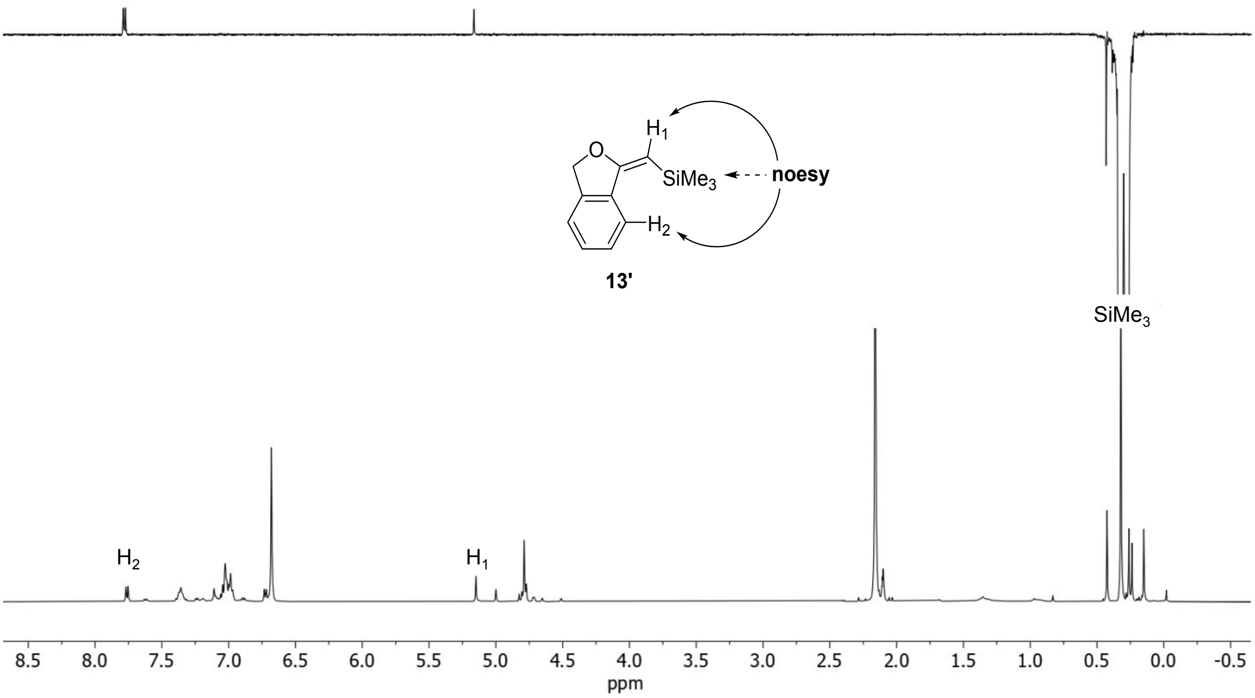

Figure S16. NOESY-1D experiment performed for cyclized product **14’**.

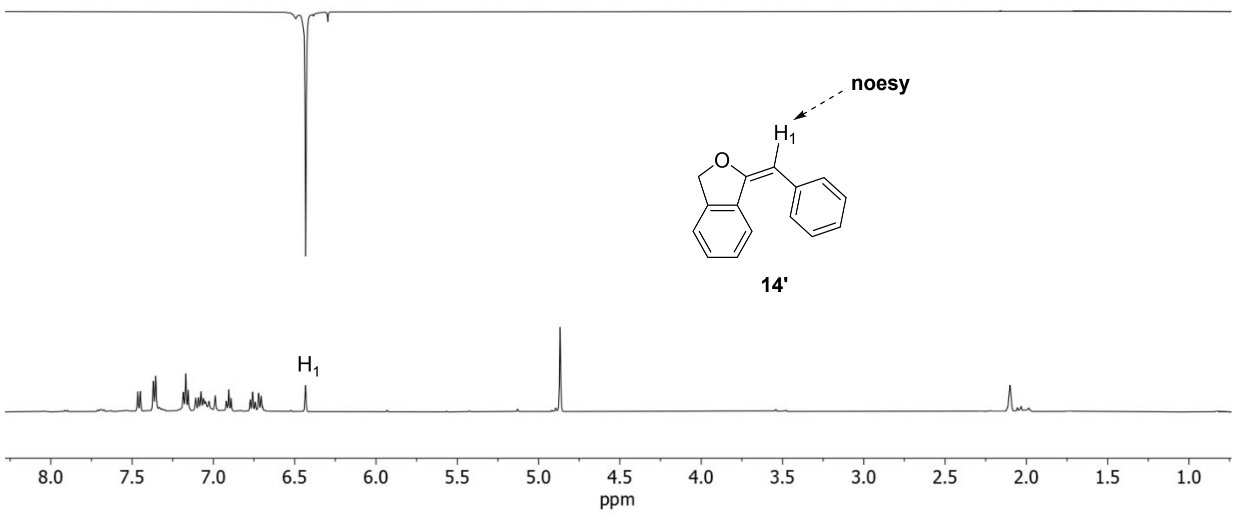

Figure S17. ^{1}H NMR monitoring of the hydroalkoxylation of **6** to **7** from t=0 to t= 2.5h mediated by catalyst **1** in toluene-d^8 at 90 ºC.

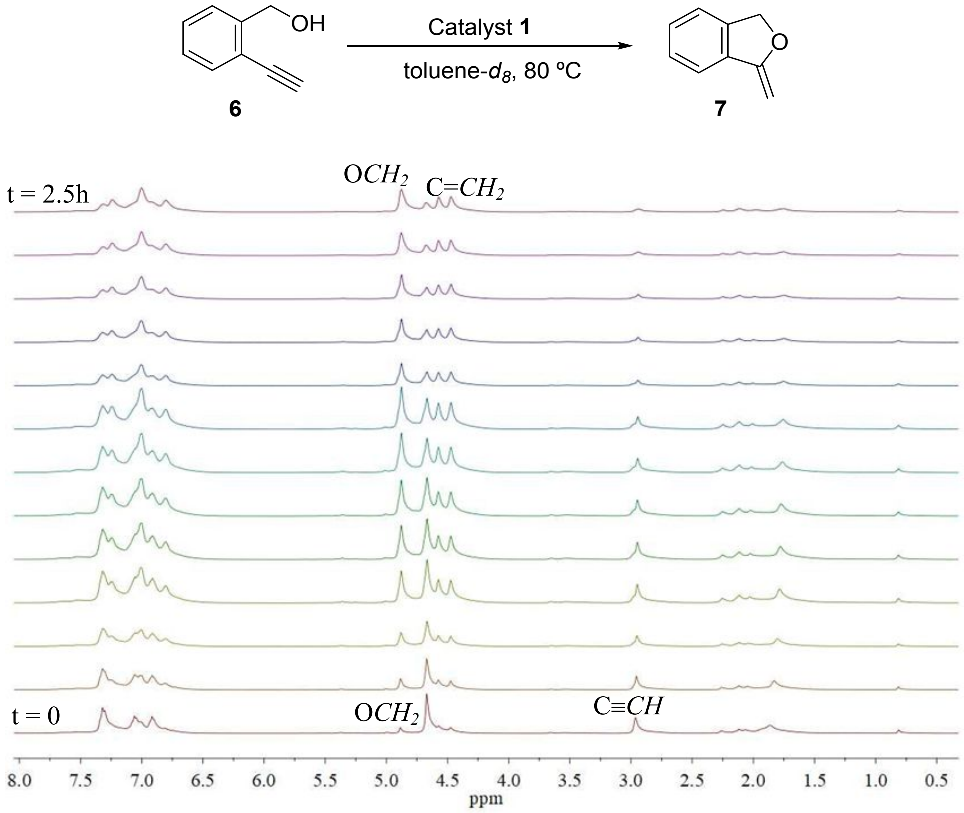

Figure S18. Plot of [**6**] versus reaction time for the hydroalkoxylation of **6** catalyzed by catalyst **1** at different concentrations of **1**. Conditions: [**6**] = 0.6 M, [**1**] = 0.007–0.035 M.

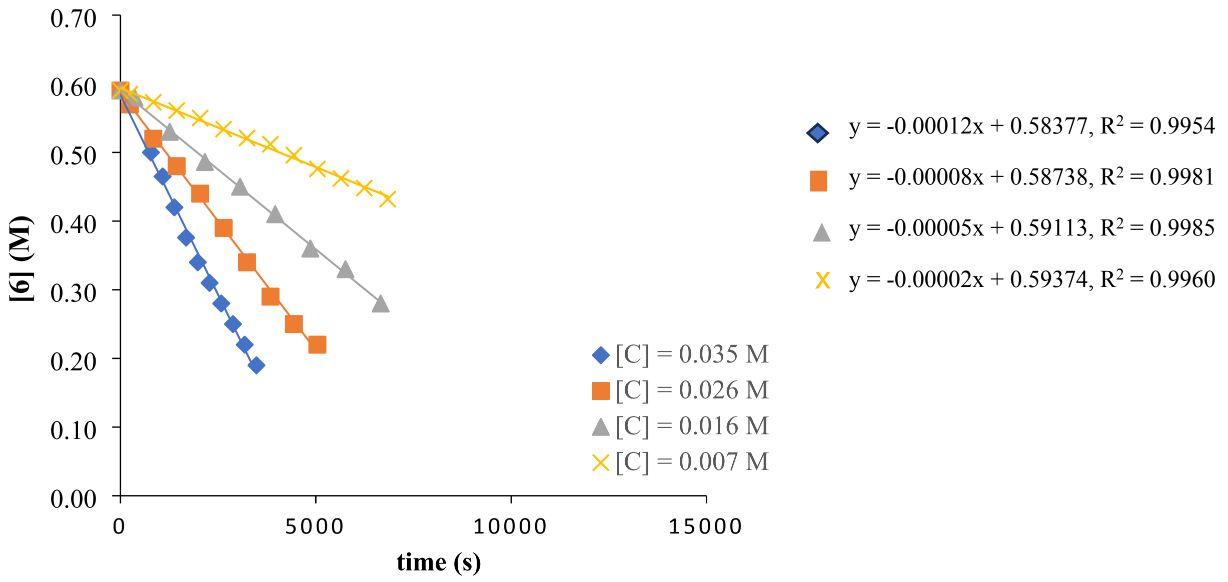

Figure S19. Arrhenius plot for the cyclization of **6** catalyzed by **1** over the temperature range 60-90 ºC in toluene-d^8.

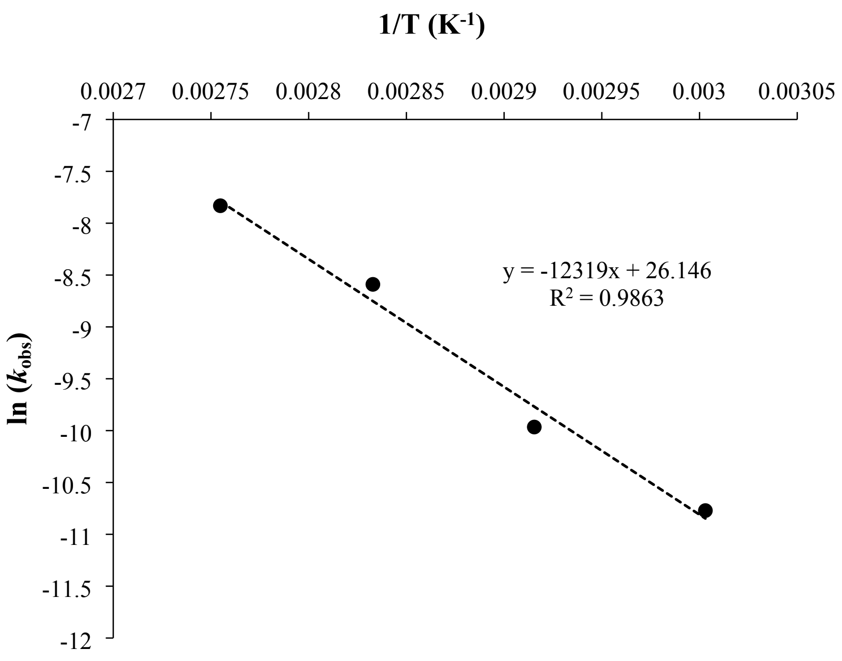

Figure S20. Eyring plot for the cyclization of **6** catalyzed by **1** over the temperature range 60-90 ºC in toluene-d^8.

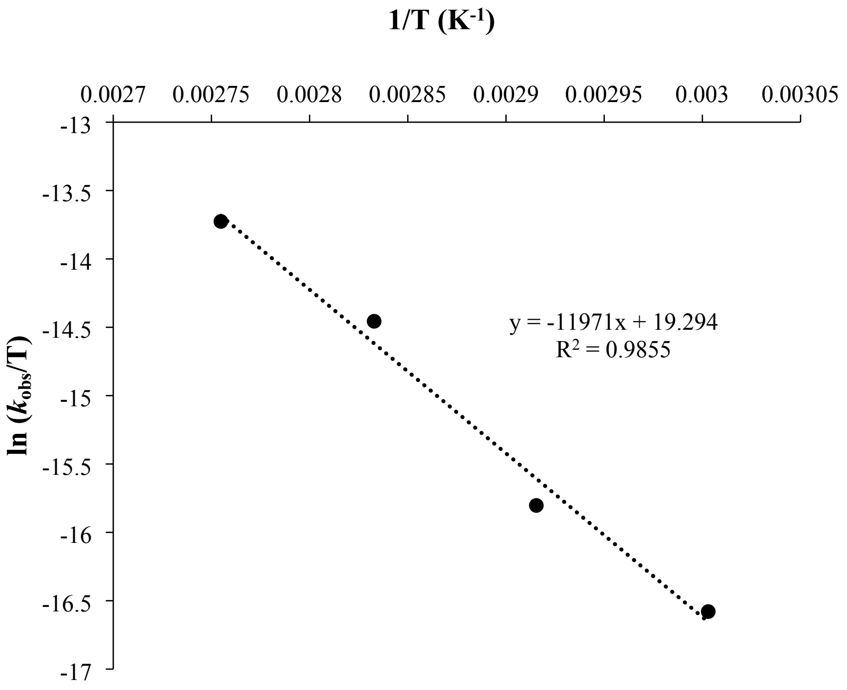

Table S3. Crystal data and structure refinement for compounds **3** and **4** (major isomer).

	**3**	**4** (major isomer)
Empirical formula	$C_{22}H_{29}N_5OZn$	$C_{20}H_{26}N_5SZn$ $\cdot 0.75(C_7H_8)$
Formula weight	447.9	502.99
Temperature (K)	100(2)	100(2)
Wavelength (Å)	0.71073	0.71073
Crystal system	Orthorhombic	Triclinic
Space group	$P2_12_12_1$	$P\bar{1}$
a(Å)	8.5772(8)	8.6029(19)
b(Å)	18.0191(14)	12.086(3)
c(Å)	28.590(3)	14.727(3)
α(°)	90	85.789(3)
β(°)	90	84.635(3)
γ(°)	90	81.683(3)
Volume($Å^3$)	4418.7(7)	1505.8(6)
Z	8	2
Density (calculated) (g/cm^3)	1.337	1.109
Absorption coefficient (mm^{-1})	1.134	0.903
F(000)	1872.0	529.0
Index ranges	$-9 \leq h \leq 9$ $-20 \leq k \leq 20$ $-33 \leq l \leq 32$	$-10 \leq h \leq 9$ $-14 \leq k \leq 14$ $-17 \leq l \leq 16$
Reflections collected	80603	9991
Independent reflections	7122 [R(int) = 0.0899]	5203 [R(int) = 0.1327]
Data / restraints / parameters	7122 / 0 / 535	5203 / 144 / 312
Goodness-of-fit on F^2	1.091	0.973
Flack parameter	0.027(15)	
Final R indices [$I>2\sigma(I)$]	R1 = 0.0364, wR2 = 0.0833	R1 = 0.0894, wR2 = 0.2463
Largest diff. peak / hole, e.$Å^{-3}$	0.45 and -0.38	1.26 and -0.54

Table S4. Selected bond distances (Å) and angles (°) for compounds **3** and **4** (major isomer).

**3**			
**Bond distances (Å)**		**Angles (º)**	
Zn(1)-N(1)	2.143(4)	N(5)-Zn(1)-N(1)	91.31(17)
Zn(1)-N(3)	2.109(4)	N(3)-Zn(1)-N(1)	84.15(16)
Zn(1)-N(5)	2.002(4)	N(5)-Zn(1)-N(3)	88.81(16)
Zn(1)-C(1)	1.981(5)	N(1)-Zn(1)-C(1)	115.70(19)
C(1)-C(2)	1.525(8)	N(3)-Zn(1)-C(1)	120.8(2)
C(14)-N(5)	1.321(6)	C(1)-Zn(1)-N(5)	140.3(2)
O(1)-C(14)	1.248(6)	N(5)-C(14)-O(1)	128.3(5)
**4** (major isomer)			
**Bond distances (Å)**		**Angles (º)**	
Zn(1)-S(1)	2.370(2)	C(19)-Zn(1)-N(3)	124.7(4)
Zn(1)-N(1)	2.081(7)	C(19)-Zn(1)-N(1)	123.4(4)
Zn(1)-N(3)	2.072(7)	N(3)-Zn(1)-N(1)	85.6(3)
Zn(1)-C(19)	1.981(10)	C(19)-Zn(1)-S(1)	126.5(3)
C(19)-C(20)	1.420(16)	N(3)-Zn(1)-S(1)	91.6(2)
S(1)-C(12)	1.726(9)	N(1)-Zn(1)-S(1)	93.9(2)
N(5)-C(12)	1.278(10)	N(5)-C(12)-S(1)	128.6(7)

3. COMPUTATIONAL DETAILS

Density Functional Theory (DFT) calculations reported in this paper were performed with the Gaussian 16 package.[6] Electron correlation was partially taken into account using the hybrid functional usually denoted as B3LYP[7] in conjunction with the D3 dispersion correction suggested by Grimme et al.[8] using the double-ζ quality def2-SVP[9] basis set for all atoms. All species were characterized by frequency calculations and have positive definite Hessian matrices. The energetic diagrams are based on electronic energies corrected with zero-point vibrational energies. Solvent effects (solvent = toluene) were taken into account using the Polarizable Continuum Model (PCM)[10] during the geometry optimizations. This level is therefore denoted PCM-(toluene)-B3LYP-D3/def2-SVP.

Input Coordinates.

[Zn(Et)(*κ*^{3}-bpzpam)] (1)

ZPE at 298 K: -2904.243115 Ha

Gibbs Energy at 298 K: -2904.304243 Ha

Z	X	Y	Z
30	0.449682000	0.976450000	0.650480000
6	1.193720000	2.511641000	1.702996000
1	0.324901000	3.128855000	2.007904000
1	1.610988000	2.120330000	2.650674000
6	2.239798000	3.411518000	1.029864000
1	3.169507000	2.859761000	0.811662000
1	2.528591000	4.281341000	1.650533000
1	1.881698000	3.813755000	0.065833000
6	-1.215688000	0.424848000	3.943983000
1	-0.489714000	1.188821000	3.633295000
1	-0.734427000	-0.228269000	4.689908000
1	-2.063710000	0.922369000	4.439749000
6	-1.676246000	-0.366393000	2.762310000
6	-2.688096000	-1.354969000	2.681501000
1	-3.323108000	-1.718965000	3.485730000
6	-2.706141000	-1.780166000	1.360378000
6	-3.545977000	-2.807157000	0.675156000
1	-4.156430000	-2.364601000	-0.129166000
1	-4.228455000	-3.271429000	1.398862000
1	-2.926203000	-3.602144000	0.230123000
6	-0.953525000	3.673014000	-1.500828000
1	-1.788057000	4.390181000	-1.519245000
1	-0.253929000	3.957671000	-2.303863000
1	-0.427021000	3.763558000	-0.540405000
6	-1.443647000	2.273728000	-1.691083000
6	-2.361972000	1.760331000	-2.639404000
1	-2.892908000	2.310863000	-3.412431000
6	-2.449780000	0.397281000	-2.390451000

6	-3.246047000	-0.669259000	-3.067623000
1	-2.595376000	-1.452565000	-3.488786000
1	-3.827885000	-0.231706000	-3.889207000
1	-3.952682000	-1.155135000	-2.374857000
6	-1.326677000	-1.108615000	-0.672769000
1	-1.918922000	-1.881955000	-1.164549000
6	0.160016000	-1.579529000	-0.834397000
7	-1.114549000	-0.206701000	1.565517000
7	-1.739983000	-1.058581000	0.721258000
7	-1.001125000	1.282773000	-0.919468000
7	-1.611222000	0.152474000	-1.341834000
7	1.076118000	-0.723314000	-0.360401000
8	0.297193000	-2.676584000	-1.383234000
6	2.456160000	-1.006144000	-0.448506000
6	3.348954000	0.086721000	-0.456646000
6	3.008755000	-2.305540000	-0.495459000
6	4.730227000	-0.100469000	-0.502709000
6	4.394141000	-2.484901000	-0.538117000
6	5.266896000	-1.391968000	-0.542655000
1	2.941273000	1.099047000	-0.433484000
1	2.345257000	-3.166782000	-0.507451000
1	5.390138000	0.771676000	-0.509935000
1	4.795932000	-3.501983000	-0.569257000
1	6.348637000	-1.543922000	-0.578911000

2-ethynylbenzyl alcohol (6)

ZPE at 298 K: -422.485672 Ha

Gibbs Energy at 298 K: -422.52016 Ha

Z	*X*	*Y*	*Z*
6	-2.473484000	-0.557715000	-0.016055000
6	-1.304522000	-1.296324000	0.195420000
6	-2.396794000	0.826102000	-0.195316000
1	-1.365011000	-2.380062000	0.327832000
1	-3.303888000	1.411769000	-0.363447000
6	-0.051032000	-0.678541000	0.239014000
6	-1.156172000	1.463831000	-0.159205000
1	-1.087971000	2.545136000	-0.295863000
6	0.024683000	0.725980000	0.055404000
1	-3.441365000	-1.064190000	-0.045467000
6	1.205852000	-1.489274000	0.440695000
1	1.804180000	-1.027662000	1.249359000
1	0.930779000	-2.508335000	0.777661000
8	1.932787000	-1.523594000	-0.776791000
1	2.856057000	-1.717866000	-0.571375000
6	1.284256000	1.408391000	0.103897000
6	2.344418000	1.998786000	0.159122000
1	3.281693000	2.522547000	0.197777000

INT-A

ZPE at 298 K: -3247.070315 Ha

Gibbs Energy at 298 K: -3247.13732 Ha

Z	X	Y	Z
30	0.262599000	-0.299334000	-0.590382000
6	-0.993698000	-1.809964000	2.544954000
1	-1.657681000	-0.993529000	2.216516000
1	-1.245100000	-2.081057000	3.579405000
1	-1.207747000	-2.672684000	1.894640000
6	0.444763000	-1.412231000	2.441375000
6	1.449770000	-1.381730000	3.438559000
1	1.347671000	-1.664433000	4.483499000
6	2.600559000	-0.907856000	2.819791000
6	3.962418000	-0.628224000	3.364872000
1	4.739605000	-1.213220000	2.846082000
1	3.997189000	-0.890041000	4.430342000
1	4.221504000	0.438516000	3.265725000
6	1.295171000	-2.435883000	-3.411417000
1	1.341886000	-3.529000000	-3.532280000
1	1.467416000	-1.985420000	-4.402390000
1	0.285357000	-2.158941000	-3.075028000
6	2.317423000	-1.964384000	-2.428761000
6	3.705084000	-2.237423000	-2.362617000
1	4.289067000	-2.858071000	-3.037896000
6	4.184011000	-1.546840000	-1.257335000
6	5.560576000	-1.451700000	-0.687449000
1	5.918807000	-0.409845000	-0.668993000
1	6.256214000	-2.040024000	-1.299675000
1	5.601848000	-1.842659000	0.342517000
6	3.038532000	-0.053829000	0.459294000
1	4.056356000	0.079563000	0.829760000
6	2.524588000	1.399754000	0.149523000
7	0.961307000	-0.989499000	1.287379000
7	2.262742000	-0.694121000	1.515974000
7	1.981332000	-1.152269000	-1.427968000
7	3.108908000	-0.900399000	-0.720987000
7	1.243110000	1.465476000	-0.244543000
8	3.346134000	2.296807000	0.348880000
6	0.583625000	2.701291000	-0.415630000
6	-0.817881000	2.720668000	-0.242387000
6	1.222969000	3.911229000	-0.761394000
6	-1.553211000	3.894004000	-0.409090000
6	0.477092000	5.081933000	-0.927820000
6	-0.910480000	5.088146000	-0.754487000
1	-1.325033000	1.793729000	0.033409000
1	2.303036000	3.926186000	-0.886040000
1	-2.637920000	3.874319000	-0.269024000
1	0.995347000	6.006248000	-1.199131000
1	-1.483340000	6.009352000	-0.886700000
6	-6.903223000	-1.440714000	0.043022000
6	-6.216826000	-0.294456000	0.458130000

6	-6.225158000	-2.428876000	-0.676807000
1	-6.746720000	0.479315000	1.021103000
1	-6.752132000	-3.327649000	-1.007663000
6	-4.861612000	-0.111302000	0.165510000
6	-4.871050000	-2.269971000	-0.974528000
1	-4.335564000	-3.039392000	-1.535089000
6	-4.169651000	-1.119628000	-0.559751000
1	-7.962908000	-1.560677000	0.281817000
6	-4.140431000	1.139529000	0.604424000
1	-3.581105000	1.542984000	-0.259645000
1	-4.883329000	1.900749000	0.910367000
8	-3.251355000	0.845160000	1.678770000
6	-2.777556000	-0.960128000	-0.840626000
6	-1.575359000	-0.753812000	-0.985068000
1	-2.643377000	1.590019000	1.779908000

C_2H_6

ZPE at 298 K: -79.699403 Ha

Gibbs Energy at 298 K: -79.720819 Ha

Z	*X*	*Y*	*Z*
6	0.000000000	0.000000000	0.764450000
1	0.000000000	1.026041000	1.168072000
1	-0.888577000	-0.513020000	1.168072000
1	0.888577000	-0.513020000	1.168072000
6	0.000000000	0.000000000	-0.764450000
1	0.888577000	0.513020000	-1.168072000
1	0.000000000	-1.026041000	-1.168072000
1	-0.888577000	0.513020000	-1.168072000

TS1-A

ZPE at 298 K: -3246.992278 Ha

Gibbs Energy at 298 K: -3247.059456 Ha

Z	*X*	*Y*	*Z*
6	7.181389000	0.239035000	-0.107071000
6	6.603481000	-1.030717000	-0.025143000
6	6.375232000	1.386588000	-0.079419000
1	7.234233000	-1.924476000	-0.049761000
1	6.828212000	2.378612000	-0.146382000
6	5.217056000	-1.176968000	0.079619000
6	4.991869000	1.262171000	0.033852000
1	4.353746000	2.148630000	0.061902000
6	4.413697000	-0.021549000	0.105646000
1	8.266465000	0.339244000	-0.194813000
6	4.470684000	-2.507942000	0.185648000
1	4.926724000	-3.201201000	-0.566087000
1	4.753642000	-2.951459000	1.178785000
8	3.136870000	-2.308012000	0.012271000
1	2.073965000	-1.206163000	0.548063000
6	2.986105000	-0.150569000	0.238647000

6	1.755670000	-0.012121000	0.480418000
30	-0.185501000	0.173573000	0.253391000
6	1.098315000	-1.910738000	-2.578827000
1	1.449882000	-0.933452000	-2.948372000
1	1.285650000	-2.657368000	-3.362955000
1	1.715796000	-2.161139000	-1.697163000
6	-0.363172000	-1.849763000	-2.268797000
6	-1.438448000	-2.520073000	-2.901542000
1	-1.375731000	-3.216634000	-3.734060000
6	-2.596932000	-2.105720000	-2.257735000
6	-4.026811000	-2.466851000	-2.489934000
1	-4.470380000	-2.961626000	-1.610270000
1	-4.100415000	-3.160397000	-3.337511000
1	-4.635553000	-1.578091000	-2.721898000
6	-0.218754000	-1.485234000	3.592159000
1	0.599768000	-0.948525000	3.091755000
1	0.106141000	-2.523040000	3.767922000
1	-0.396841000	-1.022093000	4.575127000
6	-1.455659000	-1.451048000	2.754600000
6	-2.750559000	-1.949440000	3.035297000
1	-3.083129000	-2.450003000	3.941396000
6	-3.526016000	-1.666834000	1.918550000
6	-4.966329000	-1.940656000	1.636833000
1	-5.520185000	-1.011084000	1.429026000
1	-5.427753000	-2.425777000	2.506635000
1	-5.092660000	-2.610648000	0.770776000
6	-2.986182000	-0.520491000	-0.298391000
1	-4.038044000	-0.721345000	-0.508230000
6	-2.855703000	1.044154000	-0.404293000
7	-0.848510000	-1.070304000	-1.300145000
7	-2.195963000	-1.224931000	-1.296945000
7	-1.452117000	-0.895856000	1.543506000
7	-2.701172000	-1.029789000	1.038397000
7	-1.639142000	1.541891000	-0.115262000
8	-3.894035000	1.620156000	-0.732850000
6	-1.356908000	2.925844000	-0.142733000
6	-0.135083000	3.356066000	0.422960000
6	-2.196896000	3.912660000	-0.708726000
6	0.236145000	4.699992000	0.422895000
6	-1.813893000	5.256876000	-0.705470000
6	-0.601432000	5.666516000	-0.143705000
1	0.537198000	2.623902000	0.878232000
1	-3.147512000	3.614329000	-1.141152000
1	1.189301000	4.991964000	0.872523000
1	-2.483558000	5.996585000	-1.153792000
1	-0.313483000	6.720710000	-0.145676000

INT-B

ZPE at 298 K: -3247.104731 Ha

Gibbs Energy at 298 K: -3247.171886 Ha

Z	*X*	*Y*	*Z*
6	-4.884186000	-0.329411000	-2.009070000

6	-5.397941000	-0.999719000	-0.891700000
6	-3.536261000	0.060650000	-2.047681000
1	-6.448372000	-1.301533000	-0.858996000
1	-3.150328000	0.591432000	-2.921466000
6	-4.539632000	-1.268233000	0.173027000
6	-2.680568000	-0.212793000	-0.977637000
1	-1.637646000	0.102214000	-1.021614000
6	-3.192470000	-0.884800000	0.139488000
1	-5.537947000	-0.104488000	-2.855633000
6	-4.791571000	-1.955754000	1.486781000
1	-5.550968000	-1.433558000	2.099017000
1	-5.128991000	-3.002119000	1.360371000
8	-3.534948000	-1.935392000	2.161435000
1	-1.118618000	-1.620494000	2.819767000
6	-2.553105000	-1.312575000	1.397203000
6	-1.280464000	-1.185186000	1.822828000
30	0.210647000	-0.362972000	0.833559000
6	2.233613000	-1.190193000	3.818164000
1	2.537701000	-0.592585000	4.691118000
1	2.494497000	-2.240174000	4.028027000
1	1.142692000	-1.120973000	3.705752000
6	2.915278000	-0.707683000	2.579015000
6	4.290606000	-0.471470000	2.340238000
1	5.113216000	-0.592535000	3.040908000
6	4.385681000	-0.039357000	1.024154000
6	5.570691000	0.376432000	0.216528000
1	5.698980000	-0.256545000	-0.676726000
1	6.481105000	0.290948000	0.823777000
1	5.481082000	1.421790000	-0.120541000
6	-0.711828000	-3.078794000	-1.549393000
1	-0.523573000	-4.150665000	-1.712718000
1	-1.521341000	-2.770341000	-2.230419000
1	-1.062491000	-2.922300000	-0.519576000
6	0.522502000	-2.275266000	-1.800697000
6	1.447075000	-2.350680000	-2.871553000
1	1.425119000	-3.035946000	-3.715572000
6	2.395772000	-1.364765000	-2.635204000
6	3.600042000	-0.957117000	-3.418230000
1	3.536930000	0.097035000	-3.733222000
1	3.684189000	-1.577953000	-4.319589000
1	4.527497000	-1.080312000	-2.834932000
6	2.634239000	0.364866000	-0.779031000
1	3.503534000	0.675654000	-1.360373000
6	1.705556000	1.630727000	-0.750980000
7	2.221784000	-0.432398000	1.475177000
7	3.111314000	-0.033245000	0.537115000
7	0.889850000	-1.300353000	-0.969446000
7	2.020970000	-0.756091000	-1.473121000
7	0.562909000	1.491479000	-0.058774000
8	2.154378000	2.592238000	-1.382901000
6	-0.386559000	2.538033000	0.030971000
6	-1.472801000	2.365567000	0.918921000
6	-0.349137000	3.735261000	-0.723214000
6	-2.472203000	3.328917000	1.046107000
6	-1.357902000	4.693657000	-0.588393000

6	-2.426503000	4.506089000	0.292278000
1	-1.544609000	1.450757000	1.510210000
1	0.476401000	3.904298000	-1.407829000
1	-3.297778000	3.150144000	1.740688000
1	-1.300408000	5.606508000	-1.188869000
1	-3.210041000	5.261980000	0.389213000

INT1-A'

ZPE at 298 K: -3247.077987 Ha

Gibbs Energy at 298 K: -3247.144013 Ha

Z	X	Y	Z
30	0.138575000	-0.565910000	0.482856000
6	1.326903000	-0.544855000	3.979095000
1	0.418825000	-0.965673000	3.519672000
1	1.034228000	0.195264000	4.740639000
1	1.873036000	-1.350531000	4.496048000
6	2.178100000	0.087482000	2.924862000
6	3.395004000	0.808312000	3.027566000
1	3.952563000	1.040929000	3.931739000
6	3.739328000	1.173710000	1.732581000
6	4.898293000	1.957938000	1.211642000
1	5.506550000	1.368668000	0.505783000
1	5.547099000	2.259715000	2.044104000
1	4.564614000	2.869356000	0.689595000
6	0.917554000	-3.685791000	-1.375506000
1	1.493375000	-4.602779000	-1.179447000
1	0.340767000	-3.842151000	-2.300874000
1	0.202381000	-3.529410000	-0.556505000
6	1.824516000	-2.504983000	-1.497780000
6	3.019584000	-2.353755000	-2.241034000
1	3.498741000	-3.092669000	-2.878782000
6	3.469826000	-1.063288000	-1.992905000
6	4.677380000	-0.339156000	-2.489246000
1	4.403007000	0.586983000	-3.019103000
1	5.233012000	-0.980076000	-3.185972000
1	5.356875000	-0.068375000	-1.664262000
6	2.576904000	0.810241000	-0.513629000
1	3.439534000	1.351725000	-0.905735000
6	1.336828000	1.700609000	-0.895305000
7	1.814539000	0.024408000	1.647182000
7	2.756098000	0.674687000	0.926982000
7	1.561425000	-1.369302000	-0.851822000
7	2.557314000	-0.501153000	-1.149204000
7	0.166431000	1.261375000	-0.411960000
8	1.604897000	2.703222000	-1.561693000
6	-1.028500000	2.004557000	-0.534438000
6	-1.278603000	2.959343000	-1.543555000
6	-2.046291000	1.756168000	0.411775000
6	-2.504575000	3.628745000	-1.592628000
6	-3.263925000	2.432202000	0.355809000
6	-3.504478000	3.376438000	-0.647725000
1	-0.504749000	3.173536000	-2.276613000

1	-1.886031000	1.001103000	1.186317000
1	-2.677713000	4.361205000	-2.386654000
1	-4.035286000	2.202405000	1.094885000
1	-4.460317000	3.904561000	-0.695699000
6	-5.541752000	-0.733482000	1.125203000
6	-4.397807000	-1.425169000	1.535195000
6	-5.624904000	-0.237301000	-0.179746000
1	-4.335163000	-1.811899000	2.556464000
1	-6.512945000	0.307780000	-0.508743000
6	-3.320020000	-1.640877000	0.668053000
6	-4.561553000	-0.427692000	-1.059743000
1	-4.604086000	-0.023526000	-2.072920000
6	-3.405404000	-1.119448000	-0.647109000
1	-6.368490000	-0.582093000	1.824313000
6	-2.068536000	-2.354815000	1.159324000
1	-2.366510000	-3.000425000	2.011421000
1	-1.737228000	-3.049146000	0.355087000
8	-1.062722000	-1.485556000	1.566830000
6	-2.313154000	-1.249994000	-1.565163000
6	-1.384424000	-1.310683000	-2.346919000
1	-0.559148000	-1.345335000	-3.031805000

TS1-A'

ZPE at 298 K: -3247.045269 Ha

Gibbs Energy at 298 K: -3247.110751 Ha

Z	*X*	*Y*	*Z*
30	-0.133774000	0.350341000	0.259876000
6	-0.107225000	-1.239900000	3.730740000
1	-0.737241000	-0.437284000	3.324264000
1	0.324789000	-0.920906000	4.691831000
1	-0.747859000	-2.114449000	3.931960000
6	0.972273000	-1.584540000	2.755126000
6	2.112610000	-2.408084000	2.935999000
1	2.407052000	-2.941394000	3.836899000
6	2.796121000	-2.390935000	1.729138000
6	4.072504000	-3.048643000	1.318568000
1	3.923249000	-3.738948000	0.471967000
1	4.476805000	-3.629044000	2.158122000
1	4.831165000	-2.307260000	1.019440000
6	-2.092074000	-1.340534000	-2.430373000
1	-2.483897000	-1.825437000	-3.334448000
1	-2.251068000	-0.255036000	-2.507611000
1	-2.683106000	-1.686650000	-1.568035000
6	-0.641163000	-1.654862000	-2.252517000
6	0.178306000	-2.524697000	-3.007900000
1	-0.112954000	-3.116934000	-3.871945000
6	1.441506000	-2.466211000	-2.432585000
6	2.715619000	-3.149176000	-2.806617000
1	3.507776000	-2.420322000	-3.041893000
1	2.553117000	-3.775061000	-3.693695000
1	3.084819000	-3.799707000	-1.996932000
6	2.372440000	-1.166203000	-0.457603000

1	3.298272000	-1.661356000	-0.754318000
6	2.696288000	0.366467000	-0.564165000
7	0.965938000	-1.100479000	1.514506000
7	2.064246000	-1.591827000	0.897479000
7	0.082779000	-1.098497000	-1.278628000
7	1.337852000	-1.595868000	-1.389125000
7	1.722673000	1.182310000	-0.135171000
8	3.818018000	0.623738000	-1.010593000
6	1.890157000	2.582450000	-0.106262000
6	2.774260000	3.302949000	-0.939926000
6	1.096588000	3.318655000	0.800589000
6	2.846795000	4.696676000	-0.860541000
6	1.177426000	4.708837000	0.875427000
6	2.055089000	5.412306000	0.042921000
1	3.407863000	2.760840000	-1.637410000
1	0.403453000	2.784371000	1.454519000
1	3.538608000	5.229757000	-1.519412000
1	0.548148000	5.245105000	1.591209000
1	2.121134000	6.501875000	0.099089000
6	-6.327065000	-0.550615000	0.035215000
6	-5.247652000	-1.081071000	0.749691000
6	-6.161442000	0.612232000	-0.730201000
1	-5.375599000	-1.986227000	1.350745000
1	-7.007108000	1.027176000	-1.284258000
6	-4.003521000	-0.449764000	0.704110000
6	-4.918376000	1.244119000	-0.787671000
1	-4.778722000	2.145588000	-1.388855000
6	-3.834076000	0.715653000	-0.063746000
1	-7.302630000	-1.041788000	0.076617000
6	-2.738060000	-0.908371000	1.409199000
1	-2.972566000	-1.161835000	2.464886000
1	-2.374442000	-1.852096000	0.940092000
8	-1.831180000	0.132506000	1.304981000
6	-2.508798000	1.291170000	-0.149682000
6	-1.529813000	1.910905000	-0.640738000
1	-1.227264000	2.654499000	-1.364801000

INT-B’

ZPE at 298 K: -3247.103482 Ha

Gibbs Energy at 298 K: -3247.17019 Ha

Z	*X*	*Y*	*Z*
30	0.068523000	0.371997000	-0.021879000
6	-0.945505000	-1.391946000	3.069788000
1	-1.663466000	-1.445662000	2.237393000
1	-1.054090000	-0.400140000	3.538493000
1	-1.194147000	-2.154533000	3.820838000
6	0.443567000	-1.587675000	2.552066000
6	1.516161000	-2.342844000	3.084385000
1	1.519531000	-2.930159000	3.999517000
6	2.577246000	-2.184106000	2.201249000
6	3.964795000	-2.735477000	2.232801000
1	4.164211000	-3.392647000	1.370208000

1	4.107008000	-3.328328000	3.145743000
1	4.718870000	-1.932092000	2.225546000
6	-0.953147000	-1.489737000	-3.034435000
1	-1.206238000	-2.266690000	-3.768781000
1	-1.046397000	-0.506700000	-3.523447000
1	-1.677383000	-1.516017000	-2.205840000
6	0.428637000	-1.692869000	-2.500625000
6	1.459321000	-2.553947000	-2.948012000
1	1.431175000	-3.228147000	-3.800683000
6	2.526340000	-2.366428000	-2.077864000
6	3.879693000	-2.996007000	-2.040948000
1	4.678789000	-2.239638000	-2.098750000
1	3.994223000	-3.679246000	-2.892546000
1	4.032567000	-3.579516000	-1.118100000
6	2.797865000	-0.914410000	0.000958000
1	3.805631000	-1.333357000	0.009949000
6	3.007555000	0.639178000	-0.054160000
7	0.835004000	-1.001874000	1.421919000
7	2.120656000	-1.364785000	1.210878000
7	0.851446000	-1.016676000	-1.433914000
7	2.115739000	-1.425923000	-1.179638000
7	1.879187000	1.359783000	0.014801000
8	4.184115000	1.000157000	-0.142940000
6	1.904763000	2.770968000	0.003163000
6	2.917905000	3.548051000	-0.601417000
6	0.827001000	3.452575000	0.609688000
6	2.836510000	4.943183000	-0.600518000
6	0.756040000	4.845731000	0.608894000
6	1.761582000	5.605409000	0.001122000
1	3.768491000	3.049905000	-1.060422000
1	0.039137000	2.870712000	1.093905000
1	3.632128000	5.520936000	-1.080165000
1	-0.092194000	5.340381000	1.090603000
1	1.708862000	6.697126000	-0.001590000
6	-7.082367000	0.206844000	-0.067979000
6	-6.343507000	-0.981982000	-0.008810000
6	-6.431751000	1.450478000	-0.118854000
1	-6.851132000	-1.949596000	0.030546000
1	-7.024771000	2.367493000	-0.165028000
6	-4.952505000	-0.898043000	-0.001991000
6	-5.036242000	1.528176000	-0.111295000
1	-4.527767000	2.494534000	-0.150912000
6	-4.299191000	0.340014000	-0.052235000
1	-8.174552000	0.166958000	-0.074859000
6	-3.901795000	-1.973991000	0.054031000
1	-3.963874000	-2.581424000	0.976324000
1	-3.953965000	-2.665794000	-0.807163000
8	-2.655555000	-1.274454000	0.027314000
6	-2.846373000	0.113501000	-0.030932000
6	-1.807246000	0.962714000	-0.057698000
1	-2.101142000	2.018262000	-0.109960000

INT-C’

ZPE at 298 K: -3669.614354 Ha

Gibbs Energy at 298 K: -3669.695431 Ha

Z	X	Y	Z
30	-0.585396000	0.247707000	-0.229154000
6	-3.718141000	0.034036000	-2.315403000
1	-3.764198000	-0.605876000	-1.421347000
1	-3.046073000	-0.461317000	-3.034384000
1	-4.717007000	0.107189000	-2.767446000
6	-3.201119000	1.388077000	-1.944523000
6	-3.701930000	2.671854000	-2.275269000
1	-4.568817000	2.904723000	-2.889255000
6	-2.858438000	3.585516000	-1.656187000
6	-2.879074000	5.078908000	-1.636667000
1	-3.025938000	5.473834000	-0.617506000
1	-3.704781000	5.446346000	-2.260038000
1	-1.940213000	5.502307000	-2.028496000
6	-1.417765000	-0.058888000	3.342363000
1	-1.891469000	-0.750815000	2.629366000
1	-1.997296000	-0.043495000	4.276192000
1	-0.407130000	-0.436845000	3.561693000
6	-1.336970000	1.307479000	2.743330000
6	-1.501693000	2.574016000	3.355077000
1	-1.726332000	2.777695000	4.399397000
6	-1.317138000	3.515612000	2.350667000
6	-1.362614000	5.007821000	2.386302000
1	-0.411332000	5.447764000	2.046227000
1	-1.549435000	5.349333000	3.412815000
1	-2.166391000	5.407858000	1.746250000
6	-0.834463000	3.285006000	-0.140236000
1	-0.857560000	4.375766000	-0.112953000
6	0.594855000	2.927370000	-0.674321000
7	-2.117185000	1.516836000	-1.183615000
7	-1.913682000	2.841150000	-1.010485000
7	-1.062054000	1.473326000	1.451299000
7	-1.053542000	2.804862000	1.216974000
7	0.798864000	1.622406000	-0.895066000
8	1.348153000	3.892188000	-0.835611000
6	2.018617000	1.155745000	-1.429557000
6	3.274585000	1.749024000	-1.178334000
6	1.984589000	-0.007381000	-2.224947000
6	4.442153000	1.175877000	-1.686778000
6	3.154347000	-0.566344000	-2.739389000
6	4.395071000	0.017963000	-2.469692000
1	3.324333000	2.654701000	-0.577702000
1	1.017821000	-0.475234000	-2.426299000
1	5.405441000	1.638740000	-1.455429000
1	3.096181000	-1.476485000	-3.342256000
1	5.314485000	-0.429786000	-2.853971000
6	-3.470477000	-6.325901000	0.043666000
6	-4.133708000	-5.242262000	0.634294000
6	-2.241829000	-6.140663000	-0.611102000
1	-5.089908000	-5.388073000	1.144248000
1	-1.738861000	-6.997914000	-1.066007000
6	-3.545147000	-3.981458000	0.554935000
6	-1.656607000	-4.873678000	-0.687196000

1	-0.700485000	-4.727150000	-1.195848000
6	-2.320579000	-3.791829000	-0.097996000
1	-3.912326000	-7.324339000	0.092404000
6	-3.998608000	-2.646728000	1.082918000
1	-4.971839000	-2.330594000	0.664011000
1	-4.089464000	-2.635785000	2.185523000
8	-2.984472000	-1.725354000	0.677857000
6	-1.952589000	-2.369844000	-0.017656000
6	-0.887835000	-1.693276000	-0.476519000
1	-0.176854000	-2.330337000	-1.019191000
1	0.935052000	-1.431436000	1.677482000
8	1.181347000	-0.511515000	1.507777000
6	2.571744000	-0.320045000	1.656026000
1	2.778764000	0.695518000	1.287006000
1	2.863451000	-0.331546000	2.724927000
6	3.435131000	-1.309032000	0.901226000
6	2.879940000	-2.350806000	0.155039000
6	4.846042000	-1.183317000	0.951873000
6	3.690748000	-3.272307000	-0.514153000
6	5.655577000	-2.111666000	0.270002000
6	5.081408000	-3.154861000	-0.456051000
1	1.795284000	-2.431208000	0.072349000
1	3.230507000	-4.076520000	-1.093726000
1	6.740935000	-1.999297000	0.313891000
1	5.719795000	-3.869078000	-0.981624000
6	5.447657000	-0.108683000	1.683037000
6	5.944287000	0.811550000	2.302064000
1	6.386334000	1.626227000	2.845322000

TS2-C’

ZPE at 298 K: -3669.591896 Ha

Gibbs Energy at 298 K: -3669.671068 Ha

Z	*X*	*Y*	*Z*
30	0.260225000	-0.254776000	0.068213000
6	3.482891000	0.231867000	-2.058923000
1	4.060447000	0.559913000	-1.179789000
1	2.684301000	0.971056000	-2.207446000
1	4.153041000	0.244846000	-2.929529000
6	2.913568000	-1.135534000	-1.853282000
6	3.361857000	-2.365087000	-2.393624000
1	4.197303000	-2.520910000	-3.072095000
6	2.511312000	-3.339552000	-1.890623000
6	2.476535000	-4.816011000	-2.113900000
1	2.621141000	-5.375461000	-1.174644000
1	3.280164000	-5.104839000	-2.803888000
1	1.517949000	-5.136307000	-2.553064000
6	0.839669000	-0.559571000	3.689371000
1	1.883070000	-0.219525000	3.792942000
1	0.439122000	-0.747286000	4.696129000
1	0.271474000	0.243119000	3.196952000
6	0.771662000	-1.797936000	2.856192000
6	0.832541000	-3.157914000	3.247486000

1	0.918996000	-3.548764000	4.258524000
6	0.744880000	-3.904473000	2.079632000
6	0.728542000	-5.381696000	1.860833000
1	-0.203165000	-5.703910000	1.368416000
1	0.801804000	-5.900188000	2.825702000
1	1.572938000	-5.712909000	1.233951000
6	0.490341000	-3.234156000	-0.362494000
1	0.497426000	-4.314085000	-0.517912000
6	-0.901828000	-2.765803000	-0.917399000
7	1.854068000	-1.347157000	-1.072352000
7	1.618988000	-2.680478000	-1.096207000
7	0.651498000	-1.729128000	1.530001000
7	0.643056000	-2.999738000	1.064602000
7	-1.079508000	-1.438625000	-0.933465000
8	-1.647480000	-3.676217000	-1.288622000
6	-2.250507000	-0.854117000	-1.463520000
6	-3.524915000	-1.459052000	-1.441355000
6	-2.142605000	0.442621000	-2.005216000
6	-4.639332000	-0.772164000	-1.928608000
6	-3.259091000	1.119880000	-2.493738000
6	-4.519581000	0.517404000	-2.456436000
1	-3.630850000	-2.463037000	-1.036689000
1	-1.159891000	0.918577000	-2.032954000
1	-5.620846000	-1.252059000	-1.882355000
1	-3.145062000	2.133225000	-2.886472000
1	-5.399787000	1.051915000	-2.821368000
6	5.404672000	4.533203000	-0.451717000
6	5.295472000	3.746455000	0.701882000
6	4.364456000	4.577673000	-1.395411000
1	6.107339000	3.716091000	1.433050000
1	4.472513000	5.199389000	-2.287434000
6	4.128346000	3.007425000	0.886648000
6	3.196152000	3.836304000	-1.204011000
1	2.384487000	3.866124000	-1.934537000
6	3.090114000	3.050082000	-0.052007000
1	6.310414000	5.120834000	-0.620808000
6	3.707618000	2.079782000	1.991275000
1	4.386246000	1.216646000	2.111429000
1	3.625937000	2.587097000	2.969239000
8	2.416446000	1.605994000	1.595158000
6	2.013147000	2.161346000	0.404617000
6	0.820572000	1.839993000	-0.155436000
1	0.632896000	2.343778000	-1.111296000
1	-0.252934000	1.589689000	0.683264000
8	-0.972598000	0.894751000	1.410450000
6	-2.357708000	0.836497000	1.574453000
1	-2.747177000	-0.120022000	1.176218000
1	-2.615493000	0.830430000	2.656207000
6	-3.149270000	1.956973000	0.926049000
6	-2.527889000	3.092620000	0.399005000
6	-4.562903000	1.865783000	0.851556000
6	-3.268267000	4.110134000	-0.209927000
6	-5.302346000	2.893400000	0.233986000
6	-4.659795000	4.009795000	-0.299405000
1	-1.442277000	3.175777000	0.455235000

1	-2.753687000	4.983857000	-0.618979000
1	-6.388847000	2.799259000	0.173843000
1	-5.243488000	4.798520000	-0.780558000
6	-5.250227000	0.733235000	1.397393000
6	-5.834986000	-0.223349000	1.866162000
1	-6.348478000	-1.074245000	2.273367000

1-methylene-1,3-dihydroisobenzofuran, (7)

ZPE at 298 K: -422.539992 Ha

Gibbs Energy at 298 K: -422.572128 Ha

Z	*X*	*Y*	*Z*
6	2.591838000	0.149768000	0.000056000
6	1.667907000	1.202152000	0.000026000
6	2.158423000	-1.186215000	0.000021000
1	2.008808000	2.240745000	0.000021000
1	2.896223000	-1.992338000	0.000004000
6	0.309600000	0.889502000	-0.000014000
6	0.795490000	-1.495073000	-0.000037000
1	0.457659000	-2.533976000	-0.000073000
6	-0.125032000	-0.441769000	-0.000021000
1	3.662344000	0.369605000	0.000080000
6	-0.907037000	1.773076000	-0.000193000
1	-0.964259000	2.422262000	0.892392000
1	-0.964304000	2.421427000	-0.893411000
8	-2.017816000	0.869326000	0.000287000
1	-3.531662000	-1.302942000	0.000005000
6	-1.595509000	-0.436677000	0.000058000
6	-2.453621000	-1.469425000	-0.000072000
1	-2.074636000	-2.491422000	-0.000261000

4. References

(1) Seo, S. Y.; Yu, X.; Marks, T. J. Intramolecular Hydroalkoxylation/Cyclization of Alkynyl Alcohols Mediated by Lanthanide Catalysts. Scope and Reaction Mechanism. *J. Am. Chem. Soc.* **2009**, *131*, 263–276.

(2) Hartak, H.; Weibel, J-M.; Pale, P. A mild access to γ- or δ-alkylidene lactones through gold catalysis. *Tetrahedron Lett.* **2006**, *35*, 6273.

(3) SAINT v8.37, Bruker-AXS (**2016**), APEX3 v2016.1.0. Madison, Wisconsin, USA.

(4) Krause, L.; Herbst-Irmer, R.; Sheldrick, G. M.; Stalke, D. Comparison of silver and molybdenum microfocus X-ray sources for single-crystal structure determination. *J. Appl. Crystallogr*. **2015**, *48*, 3.

(5) (a) Dolomanov, O. V.; Bourhis, L. J.; Gildea, R. J.; Howard, J. A. K.; Puschmann, H. OLEX2: a complete structure solution, refinement and analysis program. *J. Appl. Crystallogr*. **2009**, *42*, 339. (b) G. M. Sheldrick, SHELX-2014, Program for Crystal.

(6) Frisch, M.J.; Trucks, G.W.; Schlegel, H.B.; Scuseria, G.E.; Robb, M.A.; Cheeseman, J.R.; Scalmani, G.; Barone, V.; Petersson, G.A.; Nakatsuji, H.; Li, X.; Caricato, M.; Marenich, A.V.; Bloino, J.; Janesko, B.G.; Gomperts, R.; Mennucci, B.; Hratchian, H.P.; Ortiz, J.V.; Izmaylov, A.F.; Sonnenberg, J.L.; Williams-Young, D.; Ding, F.; Lipparini, F.; Egidi, F.; Goings, J.; Peng, B.; Petrone, A.; Henderson, T.; Ranasinghe, D.; Zakrzewski, V.G.; Gao, J.; Rega, N.; Zheng, G.; Liang, W.; Hada, M.; Ehara, M.; Toyota, K.; Fukuda, R.; Hasegawa, J.; Ishida, M.; Nakajima, T.; Honda, Y.; Kitao, O.; Nakai, H.; Vreven, T.; Throssell, K.; Montgomery, J.; Peralta, J.A., J.E.; Ogliaro, F.; Bearpark, M.J.; Heyd, J.J.; Brothers, E.N.; Kudin, K.N.; Staroverov, V.N.; Keith, T.A.; Kobayashi, R.; Normand, J.; Raghavachari, K.; Rendell, A.P.; Burant, J.C.; Iyengar, S.S.; Tomasi, J.; Cossi, M.; Millam, J.M.; Klene, M.; Adamo, C.; Cammi, R.; Ochterski, J.W.; Martin, R.L.; Morokuma, K.; Farkas, O.; Foresman, J.B.; Fox, D. J. Gaussian 16, Revision B.01, **2016**.

(7) (a) Becke, A. D. Density-functional exchange-energy approximation with correct asymptotic behavior. *Phys. Rev. A* **1988**, *38*, 3098; (b) Perdew, J. P. Density-functional approximation for the correlation energy of the inhomogeneous electron gas. *Phys. Rev. B* **1986**, *33*, 8822.

(8) Grimme, S.; Antony, J.; Ehrlich, S.; Krieg, H.; A consistent and accurate ab initio parametrization of density functional dispersion correction (DFT-D) for the 94 elements H-Pu. *J. Chem. Phys*. **2010**, *132*, 154104.

(9) Eichkorn, K.; Treutler, O.; Öhm, H.; Häser, M.; Ahlrichs, R. Auxiliary basis sets to approximate Coulomb potentials. *Chem. Phys. Lett*. **1995**, *242*, 652.

(10) (a) Miertuš, S.; Scrocco E.; Tomasi, J. Electrostatic interaction of a solute with a continuum. A direct utilizaion of AB initio molecular potentials for the prevision of solvent effects. *Chem. Phys*. **1981**, *55*, 117; (b) Pascual-Ahuir, J. L.; Silla, E.; Tuñón, I. GEPOL: An improved description of molecular surfaces. III. A new algorithm for the computation of a solvent-excluding surface. *J. Comput. Chem*. **1994**, *15*, 1127; (c) Barone, V.; Cossi, M. Quantum Calculation of Molecular Energies and Energy Gradients in Solution by a Conductor Solvent Model. *J. Phys. Chem. A* **1998**, *102*, 1995.
